# Pentaketides and 5-*p*-Hydroxyphenyl-2-pyridone Derivative from the Culture Extract of a Marine Sponge-Associated Fungus *Hamigera avellanea* KUFA0732

**DOI:** 10.3390/md21060344

**Published:** 2023-06-02

**Authors:** Rotchana Klaram, Tida Dethoup, Fátima P. Machado, Luís Gales, Decha Kumla, Salar Hafez Ghoran, Emília Sousa, Sharad Mistry, Artur M. S. Silva, Anake Kijjoa

**Affiliations:** 1 ICBAS-Instituto de Ciências Biomédicas Abel Salazar, Rua de Jorge Viterbo Ferreira 228, 4050-313 Porto, Portugal; mmayrotchana1993@gmail.com; 2 Department of Plant Pathology, Faculty of Agriculture, Kasetsart University, Bangkok 10240, Thailand; 3 Interdisciplinary Centre of Marine Environmental Research (CIIMAR), Terminal de Cruzeiros do Porto de Leixões, Av. General Norton de Matos s/n, 4450-208 Matosinhos, Portugal; maria.mfpm@hotmail.com; 4 Instituto de Biologia Molecular e Celular (i3S-IBMC), Universidade do Porto, Rua de Jorge Viterbo Ferreira 228, 4050-313 Porto, Portugal; 5 Faculty of Pharmaceutical Sciences, Burapha University, 169 Long Had Bangsaen Rd, Chonburi 20131, Thailand; decha1987@hotmail.com; 6 HEJ Research Institute of Chemistry, International Center for Chemical and Biological Sciences, University of Karachi, Karachi 75270, Pakistan; s_hafezghoran@yahoo.com; 7 Laboratório de Química Orgânica e Farmacêutica, Departamento de Ciências Químicas, Faculdade de Farmácia, Universidade do Porto, Rua de Jorge Viterbo Ferreira 228, 4050-313 Porto, Portugal; esousa@ff.up.pt; 8 Department of Chemistry, University of Leicester, University Road, Leicester LE 7 RH, UK; scm11@leicester.ac.uk; 9 Departamento de Química & QOPNA, Universidade de Aveiro, 3810-193 Aveiro, Portugal; artur.silva@ua.pt

**Keywords:** *Hamigera avellanea*, Aspergillaceae, marine sponge-associated fungus, pentaketides, dihydrochromone, 5-*p*-hydroxy-2-pyridone, anti-plant pathogenic fungal activity

## Abstract

Five undescribed pentaketide derivatives, (*R*)-6,8-dihydroxy-4,5-dimethyl-3-methylidene-3,4-dihydro-1*H*-2-benzopyran-1-one (**1**), [(3*S*,4*R*)-3,8-dihydroxy-6-methoxy-4,5-dimethyl-1-oxo-3,4-dihydro-1*H*-isochromen-3-yl]methyl acetate (**2**), (*R*)-5, 7-dimethoxy-3-((*S)*-(1-hydroxyethyl)-3,4-dimethylisobenzofuran-1(3*H*)-one (**4b**), (*S*)-7-hydroxy-3-((*S*)-1-hydroxyethyl)-5-methoxy-3,4-dimethylisobenzofuran 1(3*H*)-one (**5**), and a *p*-hydroxyphenyl-2-pyridone derivative, avellaneanone (**6**), were isolated together with the previously reported (*R*)-3-acetyl-7-hydroxy-5-methoxy-3,4-dimethylisobenzofuran-1(3*H*)-one (**3**), (*R*)-7-hydroxy-3-((*S*)-1-hydroxyethyl)-5-methoxy-3,4-dimethylisobenzofuran-1(3*H*)-one (**4a**) and isosclerone (**7**), from the ethyl acetate extract of a culture of a marine sponge-derived fungus, *Hamigera avellanea* KUFA0732. The structures of the undescribed compounds were elucidated using 1D and 2D NMR, as well as high-resolution mass spectral analyses. The absolute configurations of the stereogenic carbons in **1**, **4b**, **5**, and **6** were established by X-ray crystallographic analysis. The absolute configurations of C-3 and C-4 in **2** were determined by ROESY correlations and on the basis of their common biosynthetic origin with **1**. The crude fungal extract and the isolated compounds **1**, **3**, **4b**, **5**, **6,** and **7** were assayed for their growth inhibitory activity against various plant pathogenic fungi viz. *Alternaria brassicicola*, *Bipolaris oryzae*, *Colletotrichum capsici, C. gloeosporiodes*, *Curvularia oryzae*, *Fusarium semitectum*, *Lasiodiplodia theobromae*, *Phytophthora palmivora*, *Pyricularia oryzae*, *Rhizoctonia oryzae* and *Sclerotium rolfsii*.

## 1. Introduction

The fungi of the genus *Hamigera* (Family Aspergillaceae, Order Eurotiales) are widespread and common soil fungi, despite one species being associated with beetles [1]. Although many species of Eurotiales are rich sources of secondary metabolites due to the high number of secondary metabolite gene clusters present in their genomes, only a few chemical classes of secondary metabolites have been reported from the genus *Hamigera* [2].

Yamazaki et al. isolated two new cyclic pentapeptides, avellanins A and B, from *Hamigera avellanea* Stolk and Samson [3] while Breinholt et al. described the isolation of (*Z*, *Z*)-*N,N′*-[1-[(4-hydroxyphenyl)methylene]-2-[(4-methoxyphenyl)methylene]-1,2-ethanediyl]bis-formamide from an ethanol extract of the mycelium of *H. avellanea* (CBS 501.94) [4]. Breinholt’s group later noticed the inhibitory activity of the culture filtrates of the same fungus on the growth of the rice blast fungus, *Pyricularia oryzae*, so they used bioassay-guided fractionation to isolate two polyketides, hamigerone, and dihydrohamigerone. Hamigerone and dihydrohamigerone were tested for the in vitro growth inhibitory activity against plant pathogenic fungi, *P. oryzae* and *Venturia inaequalis*, and the results showed that a growth inhibitory activity of hamigerone against both pathogenic fungi was comparable to the commercial fungicides Prochloraz^®^ and Bitertanol ^®^ whereas dihydrohamigerone exhibited only marginal activity. However, when hamigerone was tested for its ability to protect rice plants against subsequent infection with *P. oryzae*, it was found to be far inferior to commercial fungicides [5]. Isaka et al. have described the isolation of two novel cyclopropyl diketones, hamavellones A and B, and two new 14-membered macrolactones, hamigeromycins A and B, together with a resorcyclic lactone, 89-250904-F1 (radicicol analog A), a spiro-heterocyclic γ-lactam alkaloid, pseurotin A, and anthraquinone pigments emodin, ϖ-hydroxyemodin, and emodin bianthrones from the ethyl acetate (EtOAc) extract of the culture broth and the mycelium of *H. avellanea* BCC 17816, isolated from a soil sample collected in Chiangmai, Thailand [6]. The Isaka group later isolated from the same fungus, hamigeromycins A and C–G [7].

On the other hand, in an attempt to use biological control as an alternative to commercial synthetic fungicides used in conventional farming, a member of our group (T. Dethoup) has evaluated the antagonistic activity of crude extracts of several marine-derived fungi against many plant pathogens that are causative agents of diseases of the economic crops [8], especially those that cause rice brown spot, rice dirty panicle [9] and sheath blight diseases [10]. 

Due to the in vitro inhibitory activity of hamigerone, isolated from *H. avellanea* (CBS 501.94), against the rice blast fungus, *P. oryzae*, we have examined the growth inhibitory activities of the crude EtOAc extract of a marine-derived *H. avellanea* KUFA0732, isolated from the marine sponge *Mycale* sp., which was collected from the coral reef at Samaesan Island in Chonburi province, Thailand, as well as some of the isolated compounds against various plant pathogenic fungi, viz. *Bipolaris oryzae* (brown spot of rice), *Curvularia oryzae* (leaf spot of rice), *Fusarium semitectum* (dirty particle of rice), *P. oryzae* (rice blast), *Rhizoctonia oryzae* (sheath blight of rice), *Alternaria brassicicola* (black spot of Chinese kale), *Colletotrichum capsici* (anthracnose of chili), *C. gloeosporiodes* (anthracnose of mango), *Lasiodiplodia theobromae* (fruit rot of mangosteen), *Phytophthora palmivora* (root and stem rot of durian), and *Sclerotium rolfsii* (stem rot of bean).

**Figure 1:**
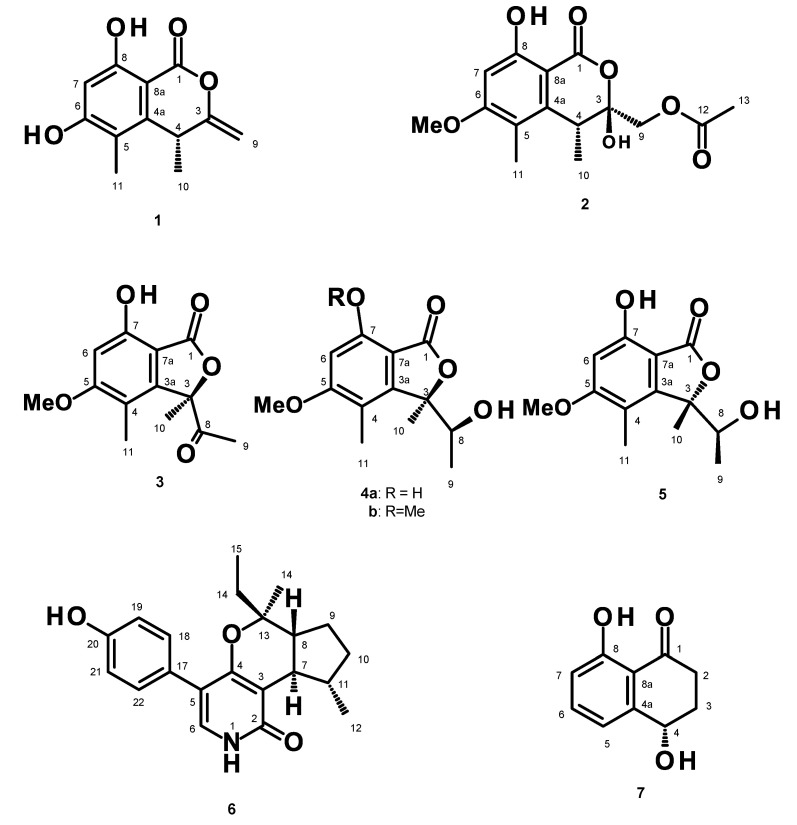
Structures of **1**–**7**.

Fractionation of the EtOAc extract of the culture *H. avellanea* KUFA0732 using silica gel column chromatography, followed by purification using preparative TLC, a Sephadex LH-20 column, and crystallization, led to the isolation of the undescribed (*R*)-6,8-dihydroxy-4,5-dimethyl-3-methylidene-3,4-dihydro-1*H*-2-benzopyran-1-one (**1**), [(3*S*,4*R*)-3,8-dihydroxy-6-methoxy-4,5-dimethyl-1-oxo-3,4-dihydro-1*H*-isochromen-3-yl]methyl acetate (**2**), (*R*)-5,7-dimethoxy-3-((*S)*-(1-hydroxyethyl)-3,4-dimethylisobenzofuran-1(3*H*)-one (**4b**), (*S*)-7-hydroxy-3-((*S*)-1-hydroxyethyl)-5-methoxy-3,4-dimethylisobenzofuran 1(3*H*)-one (**5**), and avellaneanone (**6**), together with the previously reported (*R*)-3-acetyl-7-hydroxy-5-methoxy-3,4-dimethylisobenzofuran-1(3*H*)-one (**3**) [11,12,13], (*R*)-7-hydroxy-3-((*S*)-1-hydroxyethyl)-5-methoxy-3,4-dimethylisobenzofuran-1(3*H*)-one (**4a**) [13] and isosclerone (**7**) [14,15,16] (Figure 1). The structures of the undescribed compounds were established based on an extensive analysis of the 1D and 2D NMR spectra as well as HRMS data. In the case of **1**, **4b**, **5,** and **6**, the absolute configurations of their stereogenic carbons were established by X-ray analysis, while the absolute configurations of the stereogenic carbons in **2** were determined by ROESY correlations and comparison of its ^1^H NMR data with those of **1**.

## 2. Results and Discussions

The structures of (*R*)-3-acetyl-7-hydroxy-5-methoxy-3,4-dimethylisobenzofuran-1(3*H*)-one (**3**) [11,12,13], (*R*)-7-hydroxy-3-((*S*)-1-hydroxyethyl)-5-methoxy-3,4-dimethylisobenzofuran-1(3*H*)-one (**4a**) [13] and isosclerone (**7**) [14,15,16] were elucidated by analysis of their 1D and 2D NMR spectra (Figures S14–S23 and S42–S46, Tables S1, S2 and S7) and by comparison of their NMR spectral data and the sign of their optical rotations with those reported in the literature.

**Figure 2:**
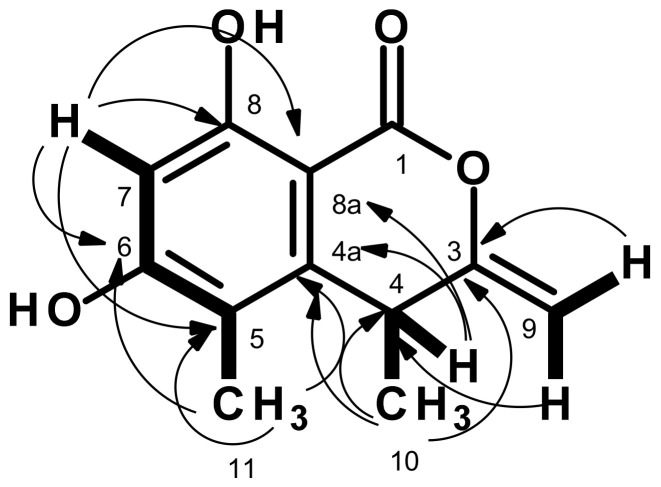
Key COSY (bold line) and HMBC (arrow) correlations in **1**.

Compound **1** was isolated as white crystals (mp = 217–218 °C), and its molecular formula C_12_H_12_O_4_ was established based on (+)-HRESIMS *m/z* 221.0814 [M + H]^+^ (calculated for C_12_H_13_O_4_, 221.0814) (Figure S6), requiring seven degrees of unsaturation. The ^13^C NMR spectrum (Table 1, Figure S2) exhibited twelve carbon signals which, in combination with DEPT and HSQC spectra (Figure S4), can be classified as one conjugated lactone carbonyl (δ_C_ 166.3), six non-protonated sp^2^ (δ_C_ 164.1, 161.8, 157.4, 143.6, 113.2, 97.7), one protonated sp^2^ (δ_C_ 101.0), one methylene sp^2^ (δ_C_ 96.2), one methine sp^3^ (δ_C_ 34.2) and two methyl (δ_C_ 22.3 and 10.2) carbons. The ^1^H NMR spectrum (Table 1, Figure S1) displayed a broad singlet of a hydrogen-bonded hydroxyl proton at δ_H_ 10.70, one aromatic singlet at δ_H_ 6.33, two doublets of the olefinic protons at δ_H_ 4.75 (*J* = 1.6 Hz) and 4.73 (*J* = 1.6 Hz), one methine quartet at δ_H_ 4.02 (*J* = 7.2 Hz), one methyl singlet at δ_H_ 2.02 and one methyl doublet at δ_H_ 1.27 (*J* = 7.0 Hz). The type and chemical shift values of the carbon atoms revealed that **1** is a 3, 4, 5, 6, 8-pentasubstituted chromone. That the hydroxyl group at δ_H_ 10.70 was on C-8, the methyl group at δ_H_ 2.02, s (δ_C_ 22.3, Me-11) was on C-5, and another hydroxyl group was on C-6 was evidenced by the chemical shift value of the hydrogen-bonded OH-8 as well as by the HMBC correlations (Table 1, Figure 2 and Figure S5) from H_3_-11 to C-5 (δ_C_ 113.2), C-4a (δ_C_ 143.6), 164.1 (C-6), and H-7 (δ_H_ 6.33, s) to C-5, C-6, C-8 (δ_C_ 161.8) and C-8a (δ_C_ 97.7). The COSY correlation from the methyl doublet at δ_H_ 1.27 (*J* = 7.0 Hz, Me-10) to the quartet at δ_H_ 4.02 (*J* = 7.2 Hz/δ_H_ 34.2) (Table 1, Figure 2 and Figure S3) revealed that Me-10 was on C-4. This was supported by HMBC correlations from H_3_-10 to C-4, C-4a, and C-3 (δ_C_ 157.4), and H-4 to C-4a, C-5, C-8a, and C-11. That the methylidene group was on C-3 was supported by HMBC correlations from the two doublets of the olefinic protons at δ_H_ 4.73 (*J* = 1.6 Hz/δ_C_ 96.2) and δ_H_ 4.75 (*J* = 1.6 Hz/δ_H_ 96.2) to C-3 and C-4 (Table 1, Figure 2 and Figure S5). Taking together the HRMS and 1D and 2D NMR data, a planar structure of **1** was elucidated as 6,8-dihydroxy-4,5-dimethyl-3-methylidene-3,4-dihydro-1*H*-2-benzopyran-1-one.

**Figure 3:**
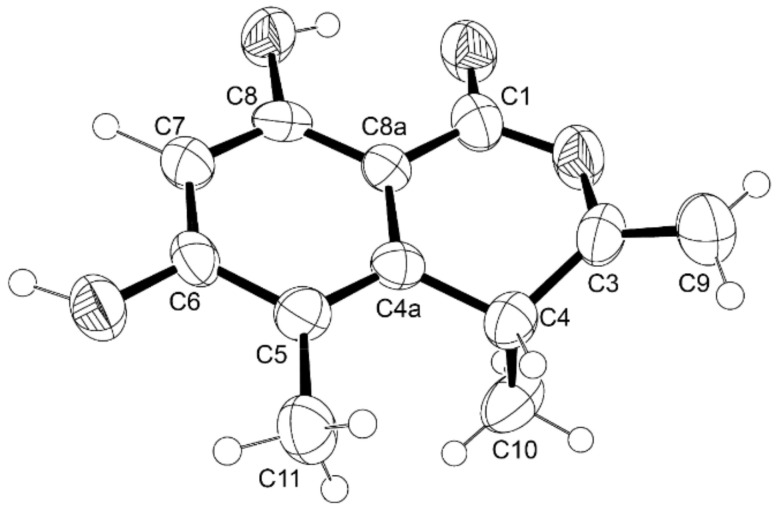
Ortep view of **1**.

Compound **1** has one stereogenic center (C-4), and it is, therefore, necessary to determine the absolute configuration of this carbon. Since **1** could be obtained as a suitable crystal for X-ray analysis using a diffractometer equipped with CuKα radiation, its stereostructure was obtained. The Ortep view of **1**, shown in Figure 3, revealed that the configuration of C-4 is *R*. Therefore, the absolute structure of **1** was elucidated as (*R*)-6,8-dihydroxy-4,5-dimethyl-3-methylidene-3,4-dihydro-1*H*-2-benzopyran-1-one.

A literature search revealed that **1** has never been previously reported; however, a structurally similar compound, but with opposite stereochemistry at C-4, i.e. (*S*)-8-hydroxy-6-methoxy-4,5-dimethyl-3-methyleneisochromen-1-one, was reported by Tayone et al. [17] from the EtOAc extract of the culture broth of *Leptosphaeria* sp. KTC 727, which was collected from woody debris in Rebun Island, Hokkaido, Japan. The absolute configuration at C-4 was determined as *S* by comparison of the calculated and experimental ECD spectra. Later, the same compound was also isolated from the mycelium and culture broth extracts of the fungus *Xylaria* sp. BL321, isolated from the leaves of the mangrove plant *Acanthus ilicifolius* L., was collected in Guangdong, China. The absolute configuration at C-4 was also determined as *S* by comparison of the calculated and experimental ECD spectra [18].

**Figure 4:**
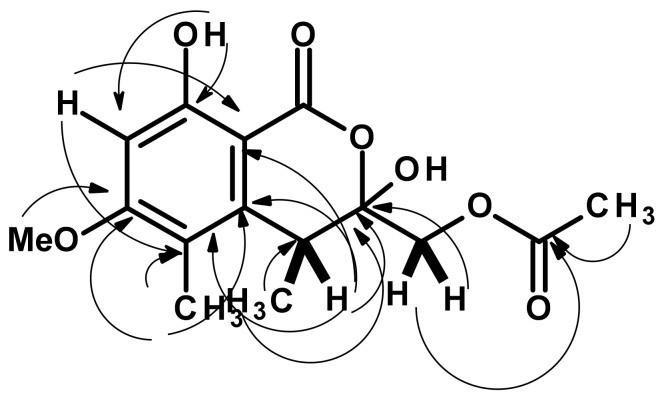
Key COSY (bold line) and HMBC (arrow) correlations in **2**.

Compound **2** was isolated as a pale yellow viscous mass. The molecular formula C_15_H_18_O_7_ of **2** was obtained from the (+)-HRESIMS *m/z* 311.1133 [M + H]+ (calculated for C_15_H_19_O_7_, 311.1131) (Figure S13), indicating seven degrees of unsaturation. The ^13^C NMR spectrum (Table 2, Figure S8) displayed 15 carbon signals which, in conjunction with DEPT and HSQC spectra (Figure S10), can be classified as one ester carbonyl (δ_C_ 170.7), one conjugated ester carbonyl (δ_C_ 168.2), two oxygen-bearing sp^2^ (δ_C_ 164.9 and 163.1), four non-protonated sp^2^ (δ_C_ 141.5, 115.5, 102.2 and 99.0), one pronated sp^2^ (δ_C_ 97.5), one oxymethylene sp^3^ (δ_C_ 65.9), one methoxy (δ_C_ 55.9), one methine sp^3^ (δ_C_ 35.9) and three methyl (δ_C_ 20.8, 16.2, and 10.1) carbons. The ^1^H NMR spectrum (Table 2, Figure S7), in combination with the HSQC spectrum (Figure S10), exhibited a singlet of a hydrogen-bonded hydroxyl proton at δ_H_ 11.14, one aromatic singlet at δ_H_ 6.37 (δ_C_ 97.5), two terminally coupled doublets at δ_H_ 4.25 (*J* = 11.8 Hz/δ_C_ 65.9) and 4.59 (*J* = 11.8 Hz/δ_C_ 65.9), one methoxyl singlet at δ_H_ 3.85 (δ_C_ 55.9), one quartet at δ_H_ 4.33 (*J* = 7.1 Hz/δ_C_ 35.9), two methyl singlets at δ_H_ 2.19 (δ_C_ 20.8) and 2.08 (δ_C_ 10.1) and one methyl doublet at δ_H_ 1.19 (*J* = 7.1 Hz/δ_C_ 16.2). The presence of a 4,5-dimethyl-8-hydroxy-6-methoxy-3,4-dihydro-1*H*-2-benzopyran-2-one was substantiated by HMBC correlations from the singlet at δ_H_ 11.14 (OH-8) to the carbons at δ_C_ 163.1 (C-8) and 97.5 (C-7), the singlet at δ_Hz/_6.37 (H-7) to C-8, the carbons at δ_C_ 115.5 (C-5) and 99.0 (C-8a), a methyl singlet at δ_H_ 2.08 (H_3_-11) to the carbon at δ_C_ 164.9 (C-6), 141.5 (C-4a) and 115.5 (C-5), and the methyl doublet at δ_H_ 1.19 (H_3_-10) to C-4a, the carbons at δ_C_ 102.2 (C-3) and 35.9 (C-4) (Table 2, Figure 4 and Figure S11). The chemical shift value of C-4 is characteristic of a hemiketal carbon, and thus one of its substituents must be a hydroxyl group. Another substituent on C-3 was an acetoxymethyl group, supported by the HMBC correlations from H_2_-9 (δ_H_ 4.25, d, *J* = 11.8 Hz) and 4.59, d, *J* = 11.8 Hz) to C-3 and the carbonyl of the acetyl group at δ_C_ 170.7 (C-12) and the methyl protons of the acetyl group (δ_H_ 2.19) to C-12 (Table 2, Figure 4 and Figure S11). 

Therefore, the planar structure of **2** was elucidated as (3,8-dihydroxy-6-methoxy-4,5-dimethyl-1-oxo-3,4-dihydro-1*H*-isochromen-3-yl)methyl acetate. 

Compound **2** has two stereogenic carbons (C-3 and C-4); therefore, it is necessary to establish their absolute configurations. Since **2** is a viscous mass and could not be obtained as a suitable crystal for X-ray diffraction, the ROESY spectrum was obtained. The ROESY spectrum (Table 2, Figure S12) showed strong correlations from H-4 to H_3_-10 and H_3_-11, confirming that H-4 is β and in the equatorial position while Me-10 is α and in the axial position. Thus, the stereochemistry at C-4 in **2** is the same as in **1**. Moreover, H_3_-10 also showed a strong correlation to H-9a and H-9b, while H-9a only showed a weak correlation to H-4. These ROESY correlations revealed that CH_2_-9 is α and OH-3 is β. Therefore, based on the absolute configuration at C-4 of **1**, the absolute configurations at C-3 and C-4 in **2** are established as 3*S*, 4*R*.

**Figure 5:**
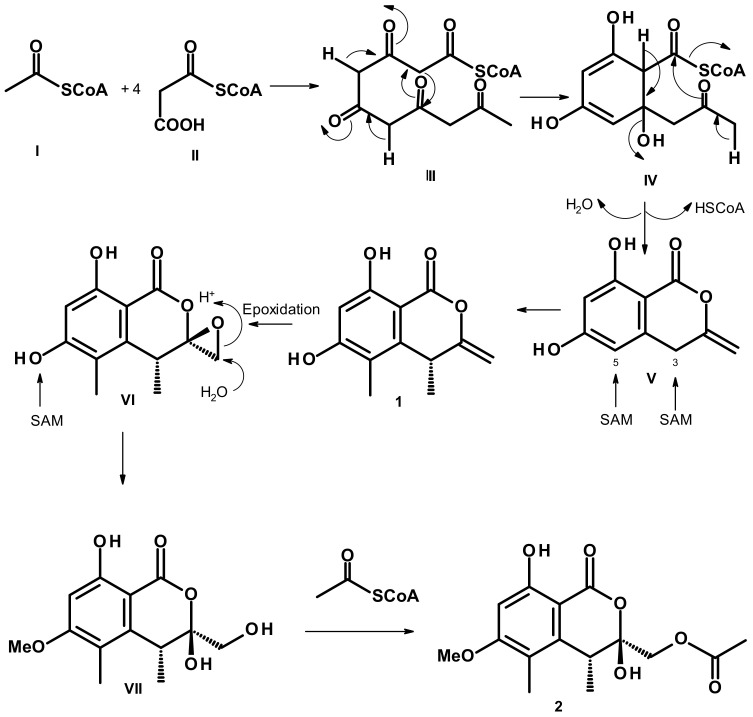
Common biosynthetic pathways of **1** and **2**.

Biosynthetically, **1** and **2** are pentaketides, and **2** could be considered to derive directly from **1** according to the proposed pathways shown in Figure 5. A Claisen condensation of one acetyl CoA (**I**) with four malonyl CoA (**II**) gives a pentaketide (**III**), which undergoes cyclization and is followed by enolization to give **IV**, and further dehydration of **IV** gives rise to **V**. Stereospecific methylation at C-3 and C-5 of **V** by *S*-adenosyl methionine (SAM) gives rise to **1**. Epoxidation of the exocyclic double bond of **1** gives an epoxide in **VI**. Enzymatic hydrolysis of the epoxide and methylation of a phenolic hydroxyl group in **VI** produces **VII**, which undergoes acetylation by acetyl CoA to give **2**. Consequently, the absolute configuration at C-4 in **2** must be the same as that of C-4 in **1**, i.e., 4*R*.

Interestingly Tayone et al. [17] have described the isolation of a mixture of two diastereomers of 3,8-dihydroxy-3-hydroxymethyl-6-methoxy-4,5-dimethylisochroman-1-one, whose structures are similar to **VII**, except for the stereochemistry at C-4 which is opposite to that of **VII**, from the EtOAc extract of the culture broth of *Leptosphaeria* sp. KTC 727. These authors have detected the difference in the ^1^H and ^13^C NMR chemical shift values between the major component (3*R*, 4*S*) and the minor component (3*S*, 4*S*) in the mixture but were not able to isolate them individually. On the contrary, the ^1^H and ^13^C NMR spectra (Figures S7 and S8) of **2** displayed clearly only one set of signals, implying that **2** is not a mixture of two diastereomers.

^1^H and ^13^C NMR data (Table S2, Figures S19 and S20) of **4a** resemble those of (*R*)-7-hydroxy-3-((*R*)-1-hydroxyethyl)-5-methoxy-3,4-dimethylisobenzofuran-1(3*H*)-one, isolated from the culture extract of a marine mangrove-associated fungus, *Xylaria* sp. BL321 [18] as well as (*R*)-7-hydroxy-3-((*S*)-1-hydroxyethyl)-5-methoxy-3,4-dimethylisobenzofuran 1(3*H*)-one, isolated from the culture extract of the fungus, *Leptosphaeria* sp. KTC 727, collected from woody debris at Akaiwa, Rebun island in Hokkaido, Japan [13]. Table S3 compared ^1^H and ^13^C NMR data of **4a** with those of both (*R*)-7-hydroxy-3-((*R*)-1-hydroxyethyl)-5-methoxy-3,4-dimethylisobenzofuran 1(3*H*)-one and (*R*)-7-hydroxy-3-((*S*)-1-hydroxyethyl)-5-methoxy-3,4-dimethylisobenzofuran 1(3*H*)-one. From the ^1^H and ^13^C NMR chemical shift values in Table S3, it is not possible to determine if the absolute configurations at C-3 and C-8 in **4a** are 3*R*, 8*R,* or 3*R*, 8*S*. However, the two stereoisomers differ in the sign of the optical rotations. While (*R*)-7-hydroxy-3-((*R*)-1-hydroxyethyl)-5-methoxy-3,4-dimethylisobenzofuran 1(3*H*)-one is dextrorotatory ([α]^25^_D_ + 355.4, c 0.05, MeOH), (*R*)-7-hydroxy-3-((*S*)-1-hydroxyethyl)-5-methoxy-3,4-dimethylisobenzofuran 1(3*H*)-one is levorotatory ([α]^25^_D_ −27, c 0.02, CDCl_3_) [13]. Since **4a** is levorotatory ([α]^25^_D_ −73, c 0.06, MeOH), we concluded that **4a** is (*R*)-7-hydroxy-3-((*S*)-1-hydroxyethyl)-5-methoxy-3,4-dimethylisobenzofuran 1(3*H*)-one.

Compound **4b** was isolated as white crystals (mp = 174–176 °C), and its molecular formula C_14_H_18_O_5_ was determined based on the (+)-HRESIMS *m/z* 267.1233 [M + H]^+^ (calculated for C_14_H_19_O_5_, 267.1232) (Figure S29), requiring six degrees of unsaturation. The ^1^H and ^13^C NMR spectra (Table 3, Figures S24 and S25) are quite similar to those of **4a**, except for the presence of an extra methoxyl substituent (δ_H_ 3.96, s/δ_C_ 56.1) on the aromatic ring and the lack of a broad signal of the phenolic hydroxyl group around 7 ppm. Thus, the structure of **4b** was deduced as a methoxy derivative of **4a**. The correlations observed in the COSY, HSQC, and HMBC spectra (Table 3, Figures S26–S28) allowed the establishment of the planar structure of **4b** as 5, 7-dimethoxy-3-(1-hydroxyethyl)-3,4-dimethylisobenzofuran 1(3*H*)-one.

**Figure 6:**
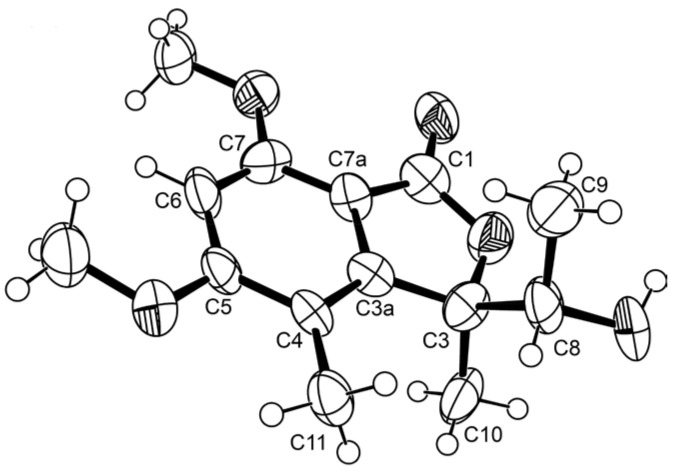
Ortep diagram of **4b**.

Since **4b** is also levorotatory ([α]^25^_D_ −300, c 0.05, MeOH) similar to **4a** ([α]^25^_D_ −73, c 0.06, MeOH), we concluded that the absolute configurations at C-3 and C-8 in **4b** are 3*R* and 8*S* also. After many attempts, we were finally able to obtain **4b** as a suitable crystal and an X-ray analysis was performed. The Ortep view of **4b** (Figure 6) confirmed the stereochemistry of C-3 and C-8 as 3*R* and 8*S*. A literature search revealed that **4b** has never been previously reported.

Compound **5** was isolated as a white crystal (mp = 116–118 °C). The (+)-HRESIMS spectrum showed the [M + H]^+^ peak at *m/z* 253.1077 (calcd for C_13_H_17_O_5_, 253.1076) (Figure S35), revealing the same molecular formula as **4a**, i.e., C_13_H_16_O_5_. Therefore, **5** is an isomer of **4a**. The ^1^H and ^13^C NMR spectra (Table 4, Figures S30 and S31) of **5** resembled those of **4a**; however, the protons of the three methyl groups in **5** showed different chemical shift values from those in **4a**. Analysis of the COSY, HSQC, and HMBC spectra (Table 4, Figures S32–S34) revealed that the planar structure of **5** is the same as that of **4a**.

**Figure 7:**
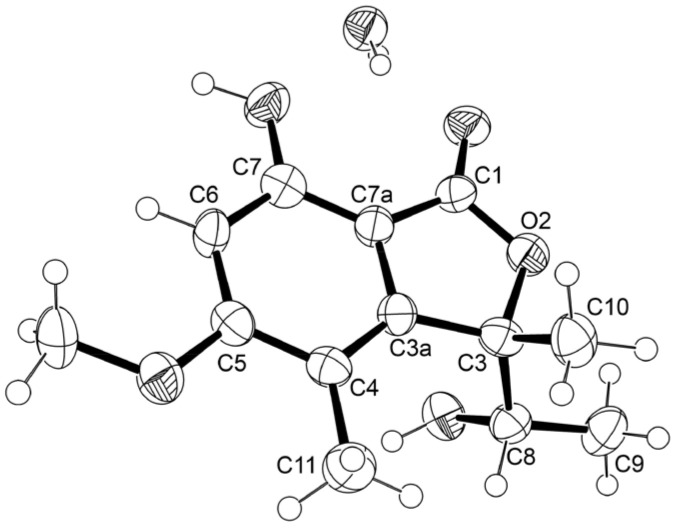
Ortep diagram of **5**.

Curiously, the proton chemical shift value of the doublet of Me-9 (δ_H_ 1.40, *J* = 6.4 Hz) in **5** is higher than that of **4a** (δ_H_ 0.89, *J* = 6.5 Hz) around 0.5 ppm while the proton chemical shift value of its Me-10 singlet (δ_H_ 1.67) was lower than that of **4a** (δ_H_ 1.78) just around 0.1 ppm. The differences in chemical shift values of the methyl protons of Me-9 and Me-10 can be attributed to the anisotropic effect of the benzene ring on both Me-9 and Me-10. As the chemical shift value of Me-10 in **5** is higher than that of its counterpart in **4a**, while Me-10 in **5** is lower than that of its counterpart in **4a**, the absolute configuration at C-3 in **5** must be opposite to that at C-3 in **4a**, i.e., 3*S*. Since **5** could be obtained in a suitable crystal, an X-ray analysis was performed. The Ortep view of **5**, shown in Figure 7, not only confirmed the 3*S* configuration but also determined the configuration at C-8 as 8*S*. Therefore, a complete structure of **5** is (*S*)-7-hydroxy-3-((*S*)-1-hydroxyethyl)-5-methoxy-3,4-dimethylisobenzofuran 1(3*H*)-one, which is the enantiomer of (*R*)-7-hydroxy-3-((*R*)-1-hydroxyethyl)-5-methoxy-3,4-dimethylisobenzofuran 1(3*H*)-one ([α]^25^_D_ +355.4, c 00.05, MeOH) [18]. This was also supported by the fact that **5** is levorotatory ([α]^25^_D_ −320, c 0.05, MeOH). From the Ortep view (Figure 7), the methyl protons of Me-10 of the α-axial 1-hydroxyethyl substituent should experience a paramagnetic field of the benzene ring, while the protons of the β-equatorial Me-9 should experience a slight diamagnetic effect of the benzene ring. A literature search revealed that **5** also has never been reported.

**Figure 8:**
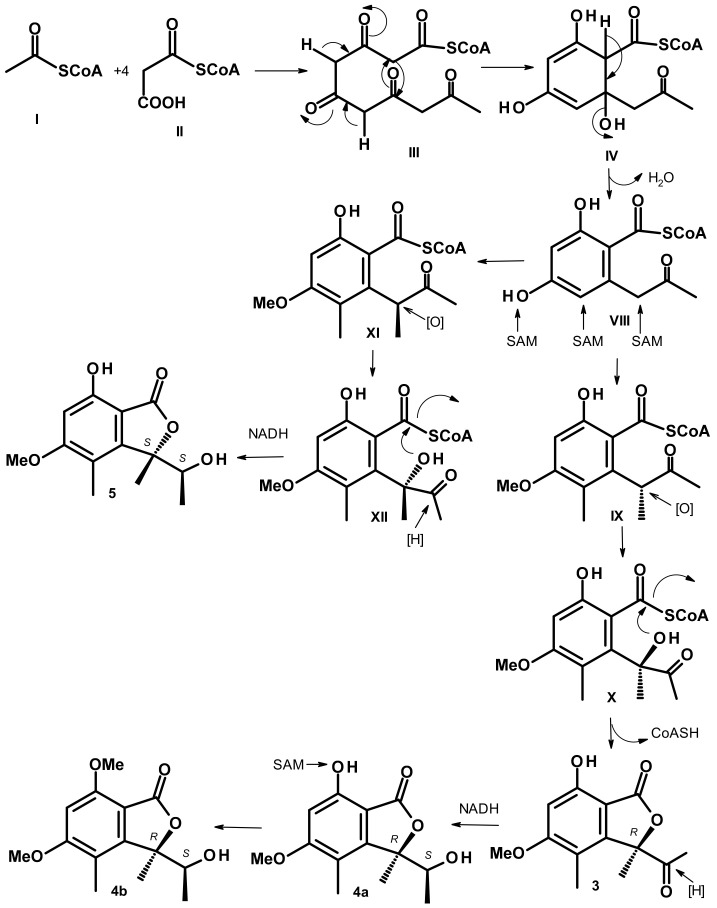
Proposed biosynthetic pathways for **3**, **4a**, **4b,** and **5**.

Analysis of the structures of **3**, **4a**, **4b**, and **5** revealed that they all derived from a cyclization of a linear pentaketide, similar to that of **1** and **2**. Figure 8 depicts proposed biosynthetic pathways for **3**, **4a**, **4b**, and **5**.

The intermediate **VIII**, derived from a cyclization and aromatization of a linear pentaketide **III**, through **IV**, undergoes stereospecific methylation at the benzyl carbon and a methylation at the aromatic carbon and the phenolic hydroxyl group to give **IX**. Stereospecific hydroxylation of the benzyl carbon of **IX** gives rise to **X**, which undergoes a lactonization to give **3**. Stereospecific reduction of the carbonyl ketone of the side chain of **3** produces **4a**. Methylation of another phenolic hydroxyl group of **4a** gives rise to **4b**.

The route leading to the formation of **5** also begins with intermediate **VIII**. In this route, the stereospecific methylation of the benzyl carbon to form **XI** occurs on the oppo face to the methylation to form **IX**. Oxidation of the benzyl carbon and lactonization of **XI** gives rise to **XII,** which, after reduction of the ketone carbonyl of the side chain, leads to the formation of **5**.

**Figure 9:**
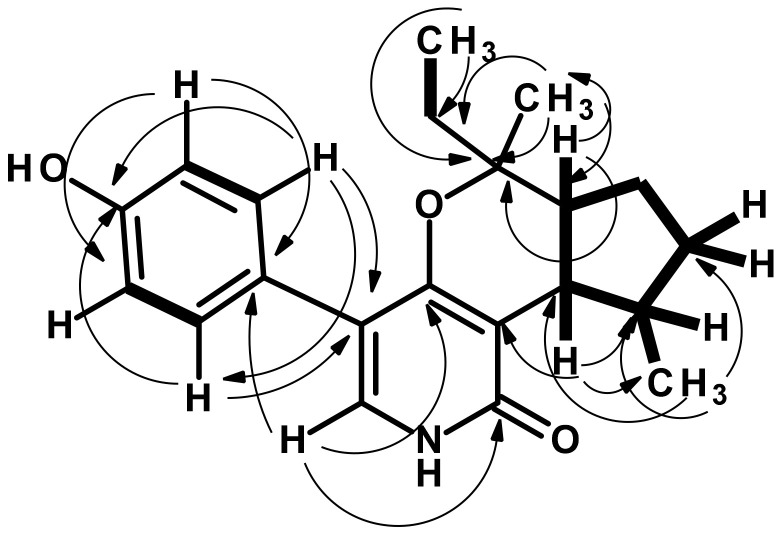
Key COSY (bold lines) and HMBC (arrow) correlations in **6**.

Compound **6** was isolated as colorless crystals (mp = 209–210 °C), and its molecular formula C_21_H_25_NO_3_ was established on the basis of (+)-HRESIMS *m/z* 362.1734 [M + Na]^+^ (calculated for C_21_H_25_NO_3_Na, 362.1732) (Figure S41), requiring ten degrees of unsaturation. The ^13^C NMR spectrum (Table 5, Figure S37) displayed 19 carbon signals which, in combination with DEPT and HSQC spectra (Figure S39), can be categorized as one conjugated amide carbonyl (δ_C_ 162.4), two oxygen-bearing sp^2^ (δ_C_ 160.2 and 156.6), three non-protonated sp^2^ (δ_C_ 110.4, 113.6 and 126.7), five protonated sp^2^ (δ_C_ 115.1 (2C), 130.6 (2C), 131.5), one oxyquaternary sp^3^ (δ_C_ 82.2), three methine sp^3^ (δ_C_ 51.9, 43.8, 35.3), three methylene sp^3^ (δ_C_ 34.5, 33.8, 25.2) and three methyl (δ_C_ 25.1, 18.6, 7.7) carbons. The ^1^H NMR spectrum (Table 5, Figure S36), in conjunction with the HSQC spectrum (Figure S39), showed two broad singlets at δ_H_ 9.38 and 10.93, two doublets of four *ortho*-coupled aromatic protons at δ_H_ 6.73 (*J* = 8.6 Hz, 2H/δ_C_ 115.1) and 7.15 (*J* = 8.6 Hz, 2H/δ_C_ 130.6), one olefinic singlet at δ_H_ 6.98 (δ_C_ 131.5), a double doublet at δ_H_ 2.06 (*J* = 12.0, 8.7 Hz/δ_C_ 43.8), a double of double doublet at δ_H_ 1.71 (*J* = 12.0, 12.0, 7.0 Hz/δ_C_ 51.9), a quartet at δ_H_ 1.55 (*J* = 7.1 Hz/δ_C_ 33.8), one methyl singlet at δ_H_ 1.11 (δ_C_ 18.6), one methyl doublet at δ_H_ 1.47 (*J* = 6.2 Hz/δ_C_ 25.1) and one methyl triplet at δ_H_ 0.85 (*J* = 7.3 Hz/δ_C_ 7.7), in addition to various overlapped multiplets. The presence of a 4-hydroxy phenyl moiety was evidenced by the COSY correlation from the doublet at δ_H_ 7.15 (*J* = 8.6 Hz/H-18, H-22) to the doublet at δ_H_ 6.73 (*J* = 8.6 Hz/H-19, H-21) (Figure S38) as well as the HMBC correlations from H-18 to C-20 (δ_C_ 156.6) and C-22 (δ_C_ 130.6), H-22 to C-18 (δ_C_ 130.6), and C-20, H-19 to C-17 (δ_C_ 126.7), and C-21 (δ_C_ 115.1), and H-21 to C-17, and C-19 (Figure S40). That another portion of **6** consists of a 3,4,5-trisubstituted 2-pyridone was substantiated by the HMBC correlations from H-6 (δ_H_ 6.98, s) to C-2 (δ_C_ 162.4), C-4 (δ_C_ 160.2), and C-5 (δ_C_ 113.6). Since H-18 and H-22 showed HMBC cross peaks to C-5 while H-6 showed cross peaks to C-17, the *p*-hydroxyphenyl group was on C-5 of the 2-pyridone ring. That the last portion of the molecule was 1-ethyl-1,5-dimethyl-1,4a,5,6,7,7a-hexahydrocyclopenta[*c*]pyran was supported by the COSY correlations from H_3_-16 (δ_H_ 0.85, t, *J* = 7.3 Hz/δ_C_ 7.7) to H-15 (δ_H_ 1.55, q, *J* = 7.1 Hz/δ_C_ 33.8), H_3_-12 (δ_H_ 1.47, d, *J* = 6.2 Hz/δ_C_ 25.1) to H-11 (δ_H_ 2.15, m/δ_C_ 35.3), and a coupling system from H_3_-12 through H-11/H-7 (δ_H_ 2.06, dd, *J* = 12.0, 8.7 Hz/δ_C_ 43.8)/H-8 (δ_H_ 1.71, ddd, *J* = 12.0, 12.0, 7.0 Hz/δ_C_ 51.9)/H-9 (δ_H_ 1.56, m/δ_C_ 25.2) and H-10 (δ_H_ 1.39, m and 1.99, m/δ_C_ 34.5) (Table 5, Figure 8 and Figure S40). This was corroborated by HMBC correlations from H_3_-16 to C-13 (δ_C_ 82.2) and C-15, H_3_-14 (δ_H_ 1.11, s/δ_C_ 18.6) to C-8, C-13, C-15, H_3_-12 to C-7, C-10 and C-11, H-7 to C-11 and C-12, H-8 to C-13 and C-14 (Table 5, Figure 8 and Figure S40). That the 3,4-dihydro-2*H*-pyran is fused with the 2-pyridone ring at C-3 and C-4 was supported by the HMBC correlation from H-7 to H-3 and the chemical shift values of C-4 and C-13 (Table 5, Figure 8 and Figure S40). Taking together the molecular formula, the COSY, and HMBC correlations, the planar structure of **6** was elucidated, as shown in Figure 9.

**Figure 10:**
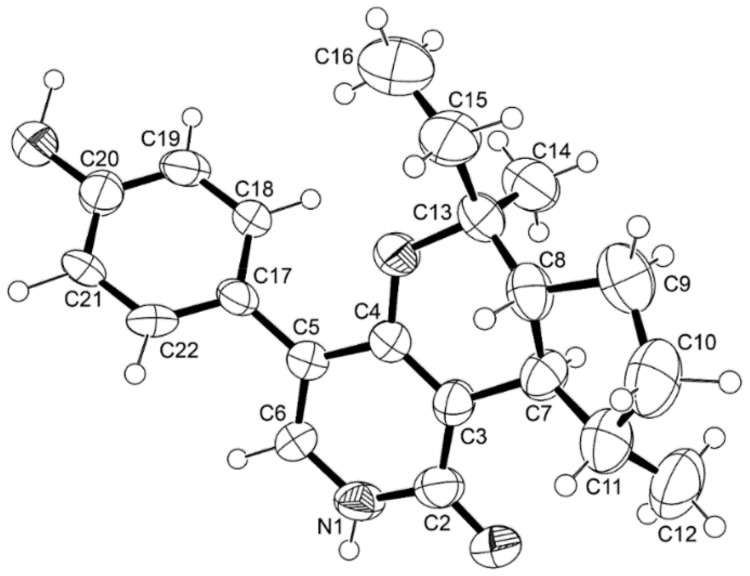
Ortep view of **6**.

Compound **6** possesses four stereogenic centers viz. C-7, C-8, C-11 and C-13. Therefore, it is challenging to determine the absolute configurations of these carbons. Since we were able to obtain a suitable crystal of **6**, its X-ray analysis was carried out using an X-ray diffractometer equipped with CuKα radiation. The Ortep view of **6** is shown in Figure 10.

The Ortep view showed clearly that the absolute configuration at C-7, C-8, C-12, and C-13 are 7*R*, 8*R*, 12*S*, and 13*S*. Since **6** has never been reported, we named it avellaneanone.

5-*p*-Hydroxy phenyl-2-pyridones with alkyl or alkenyl side chains on C-3 have been previously isolated from different fungal strains. Examples of these are tenellin [19] and desmethylbassianin, isolated from the entomopathogenic fungus, *Beauveria bassiana* [20], and aspyridone-A from *Aspergillus nidulans* [21]. Moreover, the alkyl and alkenyl side chains can undergo cyclization to form a tetrahydropyran ring such as sambutoxin, isolated from the wheat culture of *Fusarium sambucinum* which was obtained from a rot potato tuber [22], and a decalin ring system such as illicicolin H from the fermentation of *Cylindrocladium ilicicola* MFC 870 [23]. Additionally, the alkenyl side chain can form a pyran ring of a hexahydro-1*H*-2-benzopyran in trichodin A, isolated from a marine-derived *Trichoderma* sp. MF106, which was obtained from a Greenland Sea (Fram Strait) sample. However, only the relative configurations of the stereogenic carbons of the hexahydro-1*H*-2-benzopyran in trichodin A were established by analysis of NOESY correlations [24].

**Figure 11:**
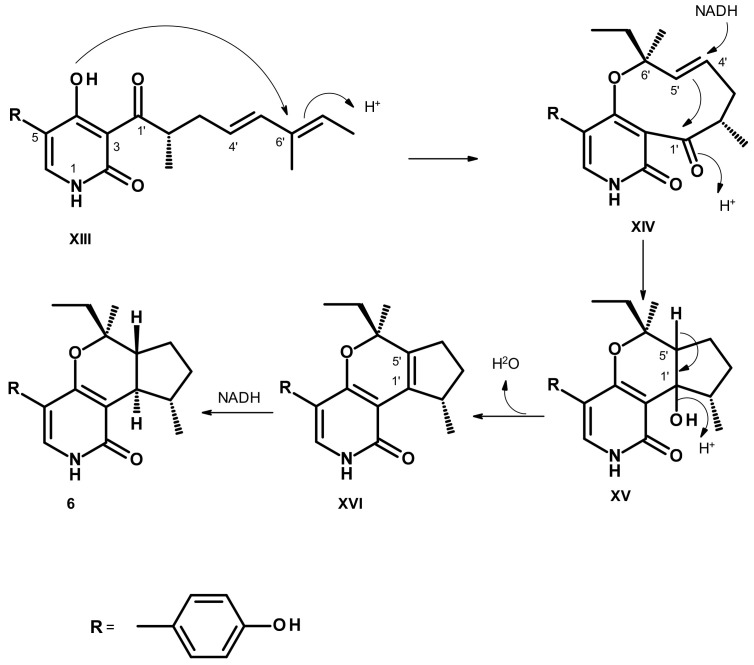
Proposed biosynthetic pathways of **6**.

The 5-*p*-Hydroxyphenyl-2-pyridone derivatives are polyketide synthase (PKS)-non-ribosomal peptide synthetase (NRPS) metabolites, which were reported to be synthesized from ring expansion of tetrameric acid intermediates catalyzed by cytochrome P450 monooxygenases [25]. Analysis of the structure of **6** revealed that the positions of the methyl groups in its doubly methylated pentaketide are different from those of tenellin [26], suggesting that *H. avellanea* KUFA 0732 must possess a different C-methylation domain (CMeT) from other fungi. Thus we propose that the biosynthetic pathways of **6** should start from the intermediate **XIII**, which is derived from a tetramic acid derivative formed by the condensation of tyrosine with a doubly methylated pentaketide. The nucleophilic addition of the hydroxyl group of the 2-pyridone ring to C-6′ of the side chain leads to the formation of the intermediate **XIV**. Reduction of C-4′ in **XIV**, with a concomitant nucleophilic addition of C-5′ to the carbonyl group (C-1′), leads to the formation of a cyclopentane ring in **XV**. Dehydration of **XV** renders a double bond between C-1′ and C-5′ in **XVI**. Stereospecific reduction of the double bond between C-1′ and C-5′ in **XVI** by dehydrogenase gives rise to **6** (Figure 11).

The crude EtOAc extract and the isolated compounds **1**, **3**, **4b**, **5**, **6,** and **7** were assayed for the growth inhibitory activity against various plant pathogenic fungi that cause diseases for economic crops, viz. *Alternaria brassicicola* (black spot of Chinese Kale), *Bipolaris oryzae* (brown spot of rice), *Colletotrichum capsici* (anthracnose of chili)*, C. gloeosporiodes* (anthracnose of mango), *Curvularia oryzae* (leaf spot of rice), *Fusarium semitectum* (dirty panicle of rice), *Lasiodiplodia theobromae* (fruit rot of mangosteen), *Phytophthora palmivora* (root and stem rot of durian), *Pyricularia oryzae* (rice blast), *Rhizoctonia oryzae* (sheath blight of rice) and *Sclerotium rolfsii* (stem rot of bean). The crude extract showed complete mycelial growth inhibition of all plant pathogenic fungi at 10 g/L, except for *F. semitectum*, *A. brassicicola* and *B. oryzae*. However, at a concentration of 1 g/L, the crude extract exhibited significant (*p* < 0.05) inhibition of the mycelial growth of *R. oryzae*, *C. capsici, C. gloeosporiodes,* and *C. oryzae*, causing more than 50% inhibition but displayed less than 50% inhibition for the rest of the pathogens tested (Table 6).

Interestingly, **3**, **5,** and **6** exhibited growth inhibition on *C. oryzae,* a causative agent of leaf spot of rice, while **1**, **2**, **4b,** and **7** were inactive (MIC > 500 µg/mL). Compounds **3** and **5** displayed strong inhibitory activity, with MIC values of 125 µg/mL, which is comparable to the positive control, mancozeb (MIC = 125 µg/Ml), while **6** only showed moderate activity, with MIC value of 250 µg/Ml. All the tested compounds were not active (MIC > 500 µg/Ml) against the rest of the plant pathogens (Table 7).

## 3. Methods and Materials

### 3.1. General Experimental Procedures

The melting points were determined on a Stuart Melting Point Apparatus SMP3 (Bibby Sterilin, Stone, Staffordshire, UK) and are uncorrected. Optical rotations were measured on an ADP410 Polarimeter (Bellingham + Stanley Ltd., Tunbridge Wells, Kent, UK). The ^1^H and ^13^C NMR spectra were recorded at ambient temperature on a Bruker AMC instrument (Bruker Biosciences Corporation, Billerica, MA, USA) operating at 300 and 75 MHz, respectively. High-resolution mass spectra were measured with a Waters Xevo QToF mass spectrometer (Waters Corporations, Milford, MA, USA) coupled to a Waters Aquity UPLC system. A Merck (Darmstadt, Germany) silica gel GF_254_ was used for preparative TLC, and Merck Si gel 60 (0.2–0.5 mm) was used for column chromatography. LiChroprep silica gel and Sephadex LH 20 were used for column chromatography.

### 3.2. Fungal Material

The fungus was isolated from a marine sponge *Mycale* sp., which was collected by scuba diving at a depth of 5-10 m from the coral reef at Samaesan Island (12°34′36.64″ N 100°56′59.69″ E), in the Gulf of Thailand, Chonburi Province, in May 2018. The sponge was washed with 0.01% sodium hypochlorite solution for 1 min, followed by sterilized seawater three times, and then dried on a sterile filter paper under sterile aseptic condition. The sponge was cut into small pieces (ca. 5 × 5 mm) and placed on Petri dish plates containing 15 mL potato dextrose agar (PDA) medium mixed with 300 mg/L of streptomycin sulfate and incubated at 28 °C for 7 days. The hyphal tips emerging from sponge pieces were individually transferred onto a PDA slant and maintained as pure cultures at Kasetsart University Fungal Collection, Department of Plant Pathology, Faculty of Agriculture, Kasetsart University, Bangkok, Thailand. The fungal strain KUFA 0732 was identified as *Hamigera avellanea* based on morphological characteristics such as colony growth rate and growth pattern on standard media, namely Czapek’s agar, Czapek yeast autolysate agar, and malt extract agar. Microscopic characteristics, including the size, shape, and ornamentation of conidiophores and spores, were examined under a light microscope. This identification was confirmed by molecular techniques using internal transcribed spacer (ITS) primers. DNA was extracted from young mycelia following a modified Murray and Thompson method [27]. The universal primer pairs, ITS1 and ITS4, were used for ITS gene amplification [28]. PCR reactions were conducted on Thermal Cycler, and the amplification process consisted of initial denaturation at 95 °C for 5 min, 34 cycles at 95 °C for 1 min (denaturation), at 55 °C for 1 min (annealing), and at 72 °C for 1.5 min (extension), followed by the final extension at 72 °C for 10 min. PCR products were examined by agarose gel electrophoresis (1% agarose with 1 × Tris-Borate-EDTA (TBE) buffer) and visualized under UV light after staining with ethidium bromide. DNA sequencing analyses were performed using the dideoxyribonucleotide chain termination method [29] by Macrogen Inc. (Seoul, Republic of Korea). The DNA sequences were edited using FinchTV software and submitted to the BLAST program for alignment and compared with that of fungal species in the NCBI database (http://www.ncbi.nlm.nih.gov/ accessed on 1 May 2023). Its gene sequences were deposited in GenBank with the accession number OQ520878.

### 3.3. Extraction and Isolation

The fungus was cultured for one week at 28 °C in five Petri dishes (i.d. 90 mm) containing 20 mL of PDA per dish. The mycelial plugs (5 mm in diameter) were transferred to two 500 mL Erlenmeyer flasks containing 200 mL of potato dextrose broth and incubated on a rotary shaker at 120 rpm at 28 °C for one week. Fifty 1000 mL Erlenmeyer flasks, each containing 300 g of cooked rice, were autoclaved at 121 °C for 15 min. After cooling to room temperature, 20 mL of a mycelial suspension of the fungus was inoculated per flask and incubated at 28 °C for 30 days, after which 500 mL of EtOAc was added to each flask of the moldy rice and macerated for 7 days, and then filtered with a Whatman No. 1 filter paper. The EtOAc solutions were combined and concentrated under reduced pressure to yield 184.1 g of a crude EtOAc extract, which was dissolved in 500 mL of CHCl_3_, washed with H_2_O (3 × 500 mL), and dried with anhydrous Na_2_SO_4_, filtered and evaporated under reduced pressure to give 153.5 g of a crude CHCl_3_ extract. The crude CHCl_3_ extract (55.5 g) was applied on a silica gel column (350 g) and eluted with mixtures of petrol-CHCl_3_ and CHCl_3_-Me_2_CO, wherein 250 mL fractions (frs) were collected as follows: frs 1–146 (petrol-CHCl_3_, 1:1), 147–204 (petrol-CHCl_3_, 3:7), 205–234 (petrol-CHCl_3_, 1:9), 235–250 (CHCl_3_), 251–322 (CHCl_3_-Me_2_CO, 9:1), 323–382 (CHCl_3_-Me_2_CO, 7:3). Frs 30–31 were combined (1.43 g) and applied over a Sephadex LH-20 column (10g) and eluted with MeOH, wherein 14 subfractions (sfrs) of 2 mL were collected. Sfrs 1–2 were combined (275.4 mg) and purified by a preparative TLC (silica gel G_254_, CHCl_3_: petrol: Me_2_CO: HCO_2_H, 90:5:5:0.1) to give 12.5 mg of **3**. Frs 39–46 were combined (397 mg) and recrystallized in CHCl_3_ to give 24.8 mg of **1**. The mother liquor of frs 39–46 (360.2 mg) was combined with frs 47–57 (305.0 mg) and purified by preparative TLC (silica gel G_254_, CHCl_3_: Me_2_CO: HCO_2_H, 90:10:0.1) to give additional 93.6 mg of **1**. Frs 62–79 were combined (710.4 mg) and applied over a Sephadex LH-20 column (10 g), and eluted with MeOH, wherein 12 sfrs of 2 mL were collected. Sfrs 2–8 were combined (540.8 mg) and applied over another Sephadex LH-20 column (10 g) and eluted with MeOH, wherein 28 sub-subfractions (ssfrs) of 2 mL were collected. Ssfrs 11–22 were combined (281.4 mg) and purified by preparative TLC (silica gel G_254_, CHCl_3_:Me_2_CO: HCO_2_H, 90:10:0.1) to give 38.5 mg of **7** and 22.4 mg of **2**. Frs 87–107 were combined (1.23 g), and part of it (543.2 mg) was purified by preparative TLC (silica gel G_254_, CHCl_3_: petrol: Me_2_CO: HCO_2_H, 90:5:5:0.1) to give 375.3 mg of a mixture of compounds which was then applied on a Sephadex LH-20 column (10 g) and eluted with MeOH, wherein 16 sfrs were collected. Sfrs 3-16 were combined to give 80 mg of **4a**. Another part of the combined frs 87–109 (670 mg) was purified by a preparative TLC (silica gel G_254_, CHCl_3_: petrol: Me_2_CO: HCO_2_H, 90:5:5:0.1) to give 19.3 mg of **4a**. Frs 117–149 were combined (264.1 mg) and crystallized in CHCl_3_ to give 15 mg of **5**. Frs 150–166 were combined (584.8 mg) and applied on a silica gel column (10 g), and eluted with mixtures of petrol-CHCl_3_, CHCl_3_, CHCl_3_-Me_2_CO, wherein 100 mL sfrs were collected as follows: sfrs 1–12 (petrol-CHCl_3_, 1:1), 13–21 (petrol-CHCl_3_, 3:7), 22–30 (petrol-CHCl_3_, 1:9), 31–73 (CHCl_3_), 74–81 (CHCl_3_-Me_2_CO, 9:1). Sfrs 35-39 were combined (143.9 mg) and purified by a preparative TLC (silica gel G_254_, CHCl_3_: Me_2_CO: HCO_2_H, 90:10:0.1) to give 18 mg of **4b** and 10 mg of **4a**. Sfrs 60–81 were combined (228.2 mg) and purified by a preparative TLC (silica gel G_254_, CHCl_3_: Me_2_CO: HCO_2_H, 90:10:0.1) to give 15.2 mg of **4b** and 5.6 mg of **4a**. Frs 326–349 were combined (1.01 g) and applied on a silica gel column (10 mg) and eluted with mixtures of petrol-CHCl_3_, CHCl_3_, CHCl_3_-Me_2_CO, wherein 100 mL sfrs were collected as follows: Sfrs 1–11 (petrol-CHCl_3_, 1:1), 12–16 (petrol-CHCl_3_, 3:7), 17–26 (CHCl_3_), 27-54 (CHCl_3_-Me_2_CO, 9:1), 55–61 (CHCl_3_-Me_2_CO, 7:3). Sfrs 33–35 were combined (154.8 mg) and crystallized in CHCl_3_ and Me_2_CO to give 64.2 mg of **6**.

#### 3.3.1. (*R*)-6,8-Dihydroxy-4,5-dimethyl-3-methylidene-3,4-dihydro-1*H*-2-benzopyran-1-one (**1**)

White crystal. mp 217–218 °C. [α${]}_{D}^{20}$ −140 (*c* 0.05, MeOH); For ^1^H and ^13^C spectroscopic data (DMSO-*d6*, 300 and 75 MHz), see Table 1; (+)-HRESIMS *m/z* 221.0814 [M + H]^+^ (calcd for C_12_H_13_O_4_, 221.0814).

#### 3.3.2. (3*S*,4*R*)-3,8-Dihydroxy-6-methoxy-4,5-dimethyl-1-oxo-3,4-dihydro-1*H*-2-benzopyran-3-yl)methyl acetate (**2**)

Pale yellow viscous mass. [α${]}_{D}^{20}$ −104 (*c* 0.05, MeOH); For ^1^H and ^13^C spectroscopic data (CDCl_3_, 300 and 75 MHz), see Table 2; (+)-HRESIMS *m/z* 311.1133 [M + H]^+^ (calcd for C_15_H_19_O_7_, 311.1131) and 333.0948 [M + Na]^+^ (calculated for C_15_H_18_O_7_Na, 333.0950).

#### 3.3.3. (*R*)-5, 7-Dimethoxy-3-((*S*)-(1-hydroxyethyl)-3,4-dimethylisobenzofuran 1(3*H*)-one (**4b**)

White crystals. mp 174–176 °C. [α${]}_{D}^{20}$ −300 (*c* 0.05, MeOH); For ^1^H and ^13^C spectroscopic data (CDCl_3_, 300 and 75 MHz), see Table 3; (+)-HRESIMS *m/z* 267.1233 [M + H]^+^ (calcd for C_14_H_19_O_5_, 267.1232).

#### 3.3.4. (*S*)-7-Hydroxy-3-((*S*)-1-hydroxyethyl)-5-methoxy-3,4-dimethylisobenzofuran 1(3*H*)-one (**5**)

White crystal. mp 116–118 °C. [α${]}_{D}^{20}$ −320 (*c* 0.05, MeOH); For ^1^H and ^13^C spectroscopic data (CDCl_3_, 300 and 75 MHz), see Table 4; (+)-HRESIMS *m/z* 253.1077 [M + H]^+^ (calcd for C_13_H_17_O_5_, 253.1076).

#### 3.3.5. Avellaneanone (**6**)

White crystal. mp 219–210 °C. [α${]}_{D}^{20}$+ 240 (*c* = 0.05, MeOH) (*c* 0.05, MeOH); For ^1^H and ^13^C spectroscopic data (DMSO-*d6*, 300 and 75 MHz), see Table 5; (+)-HRESIMS *m/z* 362.1734 [M + Na]^+^ (calcd for C_21_ H_25_ NO_3_Na, 362.1732).

### 3.4. X-ray Crystal Structures

Single crystals were mounted on a cryo-loop using paratone. X-ray CuK_α_ radiation (λ = 1.54184 Å). The structures were resolved by direct methods using SHELXS-97 and refined with SHELXL-97 [30].

Full details of data collection and refinement and tables of atomic coordinates, bond lengths, angles, and torsion angles have been deposited with the Cambridge Crystallographic Data Centre.

#### 3.4.1. X-ray Crystal Structure of **1**

The crystal was orthorhombic, space group P2_1_2_1_2_1_, cell volume 1050.3(2) Å^3^ and unit cell dimensions *a* = 5.2675(7) Å, *b* = 13.5239(16) Å, *c* = 14.7440(14) Å (uncertainties in parentheses). The calculated crystal density is 1.393 g.cm^−3^. Non-hydrogen atoms were refined anisotropically. Hydrogen atoms were directly found from different Fourier maps and refined freely with isotropic displacement parameters or placed at their idealized positions using appropriate HFIX instructions in SHELXL and included in subsequent refinement cycles. The refinement converged to R (all data) = 13.26% and wR2 (all data) = 15.30%. CCDC deposition number 2256003.

#### 3.4.2. X-ray Crystal Structure of **4b**

The crystal was triclinic, space group P1, cell volume 662.30(14) Å^3^ and unit cell dimensions *a* = 7.3973(9) Å, *b* = 8.4697(12) Å, *c* = 11.2567(13) Å and angles α = 107.628(12)°, β = 92.120(11)° and γ = 98.481(11))° (uncertainties in parentheses). The calculated crystal densities were 1.393 g.cm^−3^ and 1.335 g.cm^−3^. Two molecules were found in the asymmetric unit. Non-hydrogen atoms were refined anisotropically. Hydrogens were placed at their idealized positions using appropriate HFIX instructions in SHELXL and included in subsequent refinement cycles. The refinement converged to R (all data) = 19.00% and wR2 (all data) = 47.69%. CCDC deposition number 2260970.

#### 3.4.3. X-ray Crystal Structure of **5**

The crystal was triclinic, space group P-1, cell volume 655.52(16) Å^3^ and unit cell dimensions *a* = 7.8395(11) Å, *b* = 9.2206(12) Å, *c* = 9.8904(14) Å and angles α = 87.687(11)°, β = 85.728(11)° and γ = 66.857(13)° (uncertainties in parentheses). The calculated crystal density is 1.452 g.cm^−3^. One water molecule was found in the asymmetric unit. Non-hydrogen atoms were refined anisotropically. Hydrogens were directly found from different Fourier maps and refined freely with isotropic displacement parameters. The refinement converged to R (all data) = 10.99% and wR2 (all data) = 19.13%. CCDC deposition number 2256016.

#### 3.4.4. X-ray Crystal Structure of **6**

The crystal was monoclinic, space group I2, cell volume 1880.5(3) Å^3^ and unit cell dimensions *a* = 8.9429(8) Å, *b* = 6.4805(6) Å, *c* = 32.453(3) Å and β = 91.065(8)° (uncertainties in parentheses). The calculated crystal density is 1.199 g.cm^−3^. Non-hydrogen atoms were refined anisotropically. Hydrogen atoms were directly found from different Fourier maps and refined freely with isotropic displacement parameters or placed at their idealized positions using appropriate HFIX instructions in SHELXL and included in subsequent refinement cycles. The refinement converged to R (all data) = 7.81% and wR2 (all data) = 12.53%. CCDC deposition number 2256402.

### 3.5. Antifungal Activity Bioassays

#### 3.5.1. Antifungal Activity of the Crude Extract against Plant Pathogenic Fungi

The in vitro growth inhibitory activity of the crude EtOAc extract of *H. avellanea* KUFA0732 against eleven plant pathogenic fungi was evaluated using the poison food technique described previously by Dethoup et al. [9]. The crude EtOAc extract (1 g) was dissolved in 1 mL of DMSO and serially diluted with 9 mL of H_2_O to obtain stock solutions of 100 and 10 g/L. A volume of 1 mL of each stock solution was added to 9 mL of warm PDA, thoroughly mixed by a vortex mixer, and then poured into Petri dishes to give medium plates amended with crude extract with concentrations of 10g/L and 1 g/L, respectively. Each pathogen strain was cultured on a PDA for 7 days at 28 ± 2 °C, and a mycelial plug of 0.5 cm in diameter of each pathogen was placed on the center of the crude extract-containing PDA plates and incubated at room temperature for 14 days. A PDA plate void of the crude extract was used as a negative control. The inhibition levels were calculated using the formula: [(*x* − *y*)/*x*] × 100, where *x* = colony radius of the plant pathogenic fungi in the negative control, and *y* = colony radius of the plant pathogenic fungi in the presence of the crude extract. Each treatment was performed with five replications and repeated three times independently. The mean inhibition levels and standard deviations were calculated from five replications and three repetitions. Data were subjected to analysis of variance, and subsequently, means were compared using Duncan’s multiple range test (*p* ˂ 0.05) in the SPSS version 19 statistical program (IBM Corporation, Somers, NY, USA).

#### 3.5.2. Antifungal Activity of Isolated Compounds against Plant Pathogenic Fungi

The minimum inhibitory concentrations (MICs) for the antifungal activity of the isolated compounds on the plant pathogenic fungi were determined according to CLSI recommendation [31]. Each pathogen strain was cultured on PDA for 14 days at 28 ± 2 °C, and its spores were gently scraped by a sterile loop from the mycelium and then added to the PDA. A mass of 1 mg of each compound was dissolved in 100 µL of 10% DMSO, and then 900 µL of distilled H_2_O was added to prepare a stock solution of 1000 µg/L. Two-fold serial dilutions of the stock solution by potato dextrose broth mixed with a spore suspension of each pathogen at 10^6^ spores/mL provided the tested concentrations at 500, 250, and 125 µg/L. Each treatment was loaded at 200 µL per well into the 96-well U-shaped untreated polystyrene plates with five wells per treatment, and then the plate was incubated for 7 days at 25 °C. The MIC was determined as the lowest concentration of the compound that inhibited visible growth. Mancozeb (OXA-Sigma-Aldrich, St. Louis, MI, USA), at the same concentration, was used as a positive control.

## 4. Conclusions

The crude EtOAc extract of a solid-rice culture of a marine sponge-associated fungus, *Hamigera avellanea* KUFA0732, isolated from the marine sponge *Mycale* sp., which was collected from the Gulf of Thailand, was found to have the potential to inhibit the growth of various plant pathogenic fungi. Chemical study of the crude extract of the culture of this marine-derived fungus led to the isolation of five undescribed specialized metabolites, viz. (*R*)-6,8-dihydroxy-4,5-dimethyl-3-methylidene-3,4-dihydro-1*H*-2-benzopyran-1-one (**1**), (3*S*, 4*R*)-3,8-dihydroxy-6-methoxy-4,5-dimethyl-1-oxo-3,4-dihydro-1*H*-isochromen-3-yl]methyl acetate (**2**), (*R*)-5, 7-dimethoxy-3-((*S)*-(1-hydroxyethyl)-3,4-dimethylisobenzofuran-1(3*H*)-one (**4b**), (*S*)-7-hydroxy-3-((*S*)-1-hydroxyethyl)-5-methoxy-3,4-dimethylisobenzofuran 1(3*H*)-one (**5**), and avellaneanone (**6**), as well as three previously reported (*R*)-3-acetyl-7-hydroxy-5-methoxy-3,4-dimethylisobenzofuran-1(3*H*)-one (**3**), (*R*)-7-hydroxy-3-((*S*)-1-hydroxyethyl)-5-methoxy-3,4-dimethylisobenzofuran-1(3*H*)-one (**4a**) and isosclerone (**7**). The common biosynthetic pathways of the pentaketide derivatives **1** and **2**, as well as of **3**, **4a**, **4b,** and **5,** were determined. The biosynthesis of the PKS-NRPS hybrid product, avellaneanone (**6**), was also proposed based on the previously proposed pathways for other analogs. Finally, it is important to point out that although the crude EtOAc extract of *H. avellanea* KUFA0732 was able to completely inhibit the growth of many plant pathogenic fungi tested, only **3** and **5** displayed strong inhibitory activity (MIC = 125 µg/mL) comparable to the positive control, mancozeb, against *Curvularia oryzae*, while **6** exhibited only moderate activity (MIC = 250 µg/mL). These results give a new perspective on the potential application of marine-derived fungi and their specialized metabolites as biocontrol agents in agriculture. 

## Figures and Tables

**Table 1:** ^1^H and ^13^C NMR data (DMSO-*d_6_*, 300 and 75 MHz), COSY, and HMBC for **1**.

Position	δ_C_, Type	δ_H_, (*J* in Hz)	COSY	HMBC
1	166.3, C			
3	157.4, C			
4	34.2, CH	4.02 q (7.2)	H_3_-10	C-4a, 5, 8a, 11
4a	143.6, C			
5	113.2, C			
6	164.1, C			
7	101.0, CH	6.33,s	H_3_-11	C-5, 6, 8, 8a
8	161.8, C			
8a	97.7, C			
9	96.2, CH_2_	4.75, d (1.6) 4.73, d (1.6)		C-3, 4
10	10.2, CH_3_	1.27, d (7.0)	H-4	C-3, 4, 4a
11	22.3 CH_3_	2.02, s	H-7	C-4a, 5, 6
OH-8	-	10.70, brs		

**Table 2:** ^1^H and ^13^C NMR data (DMSO-*d_6_*, 300 and 75 MHz), COSY, HMBC, and ROESY for **2**.

Position	δ_C_, Type	δ_H_, (*J* in Hz)	COSY	HMBC	ROESY
1	168.2, CO				
3	102.2, C				
4	35.9, CH	4.33, q (7.1)	H_3_-10,	C-3, 4a, 5, 8a, 10	H_3_-10, H-11
4a	141.5, C				
5	115.5, C				
6	164.9, C				
7	97.5, CH	6.37, s		C-3, 5, 8, 8a, 12	OMe-6
8	163.1, C				
8a	99.0, C				
9a b	65.9, CH_2_	4.25, d (11.8) 4.59, d (11.8)	H-9b H-9a	C-12 C-3, 12	H_3_-10 H_3_-10
10	16.2, CH_3_	1.19, d (7.1)	H-4	C-3, 4, 4a	H-9a, 9b
11	10.1, CH_3_	2.08, s		C-3, 4a, 6	H-4
12	170.7, CO				
13	20.8, CH_3_	2.19, s		C-12	
OMe-6	55.9, CH_3_	3.85, s		C-6	H-7
OH-8	-	11.14, s		C-7, 8	

**Table 3:** ^1^H and ^13^C NMR data (DMSO-*d_6_*, 300 and 75 MHz), COSY, and HMBC for **4b**.

Position	δ_C_, Type	δ_H_, (*J* in Hz)	COSY	HMBC
1	168.1, CO			
3	88.7, C			
3a	152.8, C			
4	111.7, C			
5	164.5, C			
6	94.5, CH	6.04, s	OMe-5, H_3_-11	C-1, 4, 5, 7, 7a
7	158.3, C			
7a	105.4, C			
8	70.9, CH	4.20, m	H_3_-9	
9	17.8, CH_3_	0.86, d (6.5)	H-8	C-3, 8
10	21.5, CH_3_	1.74, s		C-3, 3a, 8
11	11.2, CH_3_	2.10, s		C-3a, 4, 5
OMe-5	56.0, CH_3_	3.91, s		C-5
OMe-7	56.1, CH_3_	3.96, s		C-7

**Table 4:** ^1^H and ^13^C NMR data (DMSO-*d_6_*, 300 and 75 MHz), COSY and HMBC for **5**.

Position	δ_C_, Type	δ_H_, (*J* in Hz)	COSY	HMBC
1	171.1, CO			
3	91.6, C			
3a	149.8, C			
4	112.4, C			
5	165.3, C			
6	98.2, CH	6.43, s	H_3_-11, OMe-5	C-1, 4,7, 7a
7	156.3, C			
7a	103.7, C			
8	70.5, CH	4.26, m	H_3_-9	
9	17.9, CH_3_	1.40, d (6.4)		C-3, 8
10	21.1, CH_3_	1.67, s		C-3, 3a, 8
11	11.1, CH_3_	2.17,s	H-6	C-3a, 4, 5
OMe-5	56.3, CH_3_	3.87, s	H-6	C-5
	OH-7	7.67, brs		

**Table 5:** ^1^H and ^13^C NMR data (DMSO-*d_6_*, 300 and 75 MHz), COSY and HMBC for **6**.

Position	δ_C_, Type	δ_H_, (*J* in Hz)	COSY	HMBC
NH-1	-	10.93, brs		
2	162.4 CO			
3	110.4 C			
4	160.2, C			
5	113.6, C			
6	131.5, CH	6.98,s		C-2, 4.5, 17
7	43.8, CH	2.06, dd (12.0, 8.7)	H-8, 11	C-3, 11, 12
8	51.9, CH	1.71, ddd (12.0, 12.0, 7.0)	H-7, 9	C-13, 14
9	25.2 CH_2_	1.56, m	H-8, 10	
10	34.5, CH_2_	1.39, m 1.99, m	H-9, 11	C-9, 11
11	35.3, CH	2.15, m	H_3_-12	
12	25.1, CH_3_	1.47, d (6.2)	H-11	C-7, 10, 11
13	82.2, C	-		
14	18.6, CH_3_	1.11, s		C-8, 13, 15
15	33.8, CH_2_	1.55, q (7.1)	H_3_-16	
16	7.7, CH_3_	0.85, t (7.3)	H-15	C-13, 15
17	126.7, C	-		
18	130.6, CH	7.15, d (8.6)	H-19	H-5, 20, 22
19	115.1, CH	6.73, d (8.6)	H-18	H-17, 20, 21
20	156.6, C	-		
21	115.1, CH	6.73, d (8.6)	H-22	H-17, 19, 20
22	130.6, CH	7.15, d (8.6)	H-21	H-5, 18, 20
23	OH-20	9.38, brs		

**Table 6:** Effects of the crude EtOAc extract of *H. avellanea* KUFA0732 on the growth of plant pathogenic fungi.

Plant Pathogen	% Mycelial Growth Inhibition at Concentrations	
	10 g/L	1 g/L
*Alternaria brassicicola* (black spot of Chinese Kale)	68.67 ± 2.27 ^c^	47.33 ± 1.62 ^d^
*Bipolaris oryzae* (brown spot of rice)	65.21 ± 3.12 ^d^	36.09 ± 1.14 ^f^
*Colletotrichum capsici* (anthracnose of chili)	100 ± 0 ^a^	58.67 ± 2.08 ^c^
*C. gloeosporiodes* (anthracnose of mango)	100 ± 0 ^a^	62.32 ± 3.58 ^b^
*Curvularia oryzae* (leaf spot of rice)	100 ± 0 ^a^	63.67 ± 2.15 ^b^
*Fusarium semitectum* (dirty panicle of rice)	85.34 ± 3.61 ^b^	38.04 ± 1.36 ^e^
*Lasiodiplodia theobromae* (fruit rot of mangosteen)	100 ± 0 ^a^	37.42 ± 1.08 ^e f^
*Phytophthora palmivora* (root and stem rot of durian)	100 ± 0 ^a^	0 ± 0 ^h^
*Pyricularia oryzae* (rice blast)	100 ± 0 ^a^	46.25 ± 2.01 ^d^
*Rhizoctonia oryzae* (sheath blight of rice)	100 ± 0 ^a^	68.34 ± 3.20 ^a^
*Sclerotium rolfsii* (stem rot of bean)	100 ± 0 ^a^	33.47 ± 1.06 ^g^

Means ± standard derivations followed by the same letter in each column are not significantly different at *p* < 0.05 when analyzed using Duncan’s test of one-way ANOVA.

**Table 7:** Effects of **1**, **3**, **4b**, **5**, **6,** and **7** on the growth of plant pathogenic fungi.

Plant Pathogen	Plant Disease	MIC (µg/Ml)						
		1	3	4b	5	6	7	Mancozeb
*Alternaria brassicicola*	black spot of Chinese Kale	>500	>500	>500	>500	>500	>500	125
*Bipolaris oryzae*	brown spot of rice	>500	>500	>500	>500	>500	>500	125
*Colletotrichum capsici*	anthracnose of chili	>500	>500	>500	>500	>500	>500	125
*C. gloeosporiodes*	anthracnose of mango	>500	500	>500	>500	>500	>500	125
*Curvularia oryzae*	leaf spot of rice	>500	125	>500	125	250	>500	125
*Fusarium semitectum*	dirty panicle of rice	>500	>500	>500	>500	>500	>500	125
*Lasiodiplodia theobromae*	fruit rot of mangosteen	>500	>500	>500	>500	>500	>500	250
*Phytophthora palmivora*	root and stem of durian	>500	>500	>500	>500	>500	>500	125
*Pyricularia oryzae*	rice blast	>500	>500	>500	>500	>500	>500	125
*Rhizoctonia oryzae*	sheath blight of rice	>500	>500	>500	>500	>500	>500	250
*Sclerotium rolfsii*	stem rot of bean	>500	>500	>500	>500	>500	>500	250

## Data Availability

The data presented in this study are available on request from the corresponding author.

## Supplementary Materials

The following are available online at https://www.mdpi.com/article/10.3390/md21060344/s1, Figures S1–S5, S7–S12, S14–S28, S30–S34, S36–S40, S42–S46: 1D and 2D NMR spectra of compounds **1**–**7**. Figures S6, S13, S29, S35, S41: HRMS data for compounds **1**, **2**, **4b**, **5**, **6**. Table S1: ^1^H and ^13^C NMR data (CDCl_3_, 300 and 75 MHz), COSY and HMBC for **3**. Table S2: ^1^H and ^13^C NMR (300 and 75 MHz, CDCl_3_), COSY, and HMBC of **4a**. Table S3: Comparison of ^1^H and ^13^C NMR data of **4a** (300 and 75 MHz, CDCl_3_) with those of (*R*)-7-hydroxy-3-((*R*)-1-hydroxyethyl)-5-methoxy-3,4-dimethylisobenzofuran-1(3*H*)-one (400 and 100 MHz, CDCl_3_) and (*R*)-7-hydroxy-3-((*S*)-1-hydroxyethyl)-5-methoxy-3,4-dimethylisobenzofuran-1(3*H*)-one (500 and 125 MHz, CDCl_3_). Table S4: ^1^H and ^13^C NMR data (DMSO-*d_6_*, 300 and 75 MHz), COSY, and HMBC for **7**.
