# Supplementary material for: Pentaketides and 5-p-Hydroxyphenyl-2-pyridone Derivative from the Culture Extract of a Marine Sponge-Associated Fungus Hamigera avellanea KUFA0732
Source: Mar Drugs. 2023 Jun 2;21(6):344. doi: 10.3390/md21060344 (PMC10304554; doi:10.3390/md21060344)
Supplement: Supplementary file 1 [file marinedrugs-21-00344-s001.zip › marinedrugs-2404843-supplementary.pdf]

Supplementary Materials

Pentaketides and 5-*p*-Hydroxyphenyl-2-pyridone Derivative from the Culture Extract of a Marine Sponge-Associated Fungus *Hamigera avellanea* KUFA0732

Figure S1. ^{1}H NMR spectrum of **1** (DMSO$_{d6}$, 300 MHz).

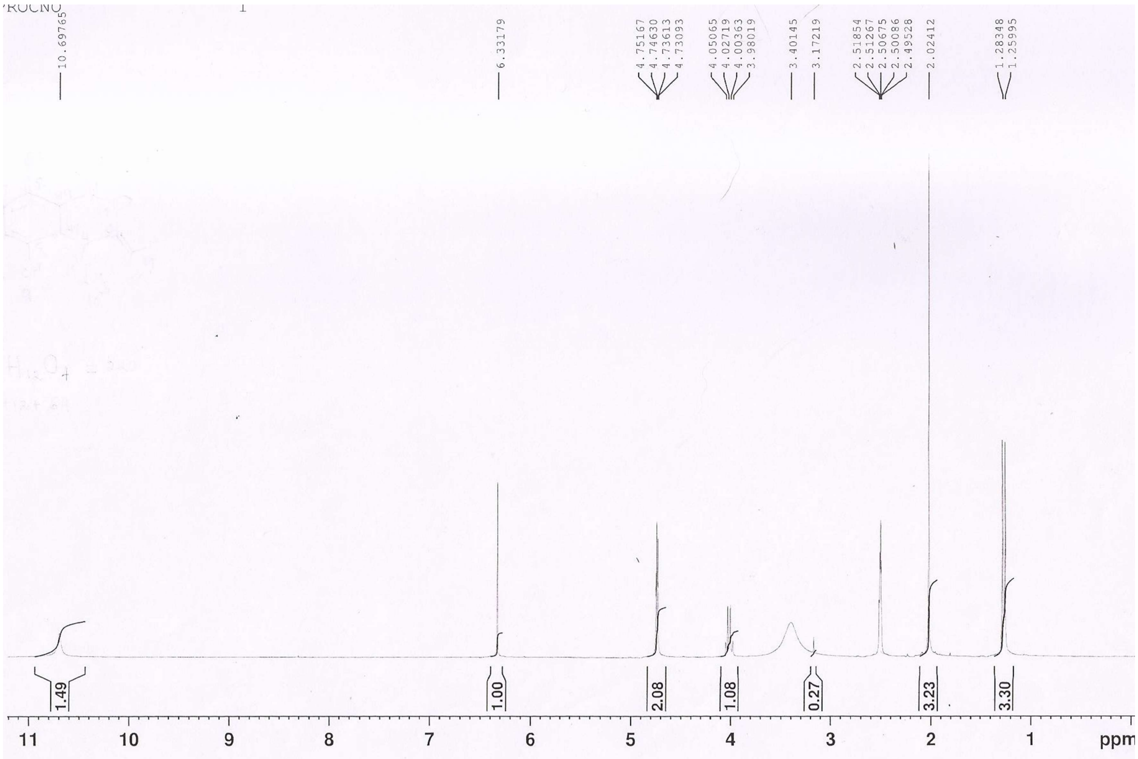

Figure S2. ^{13}C NMR spectrum of **1** (DMSO$_{d6}$, 75 MHz).

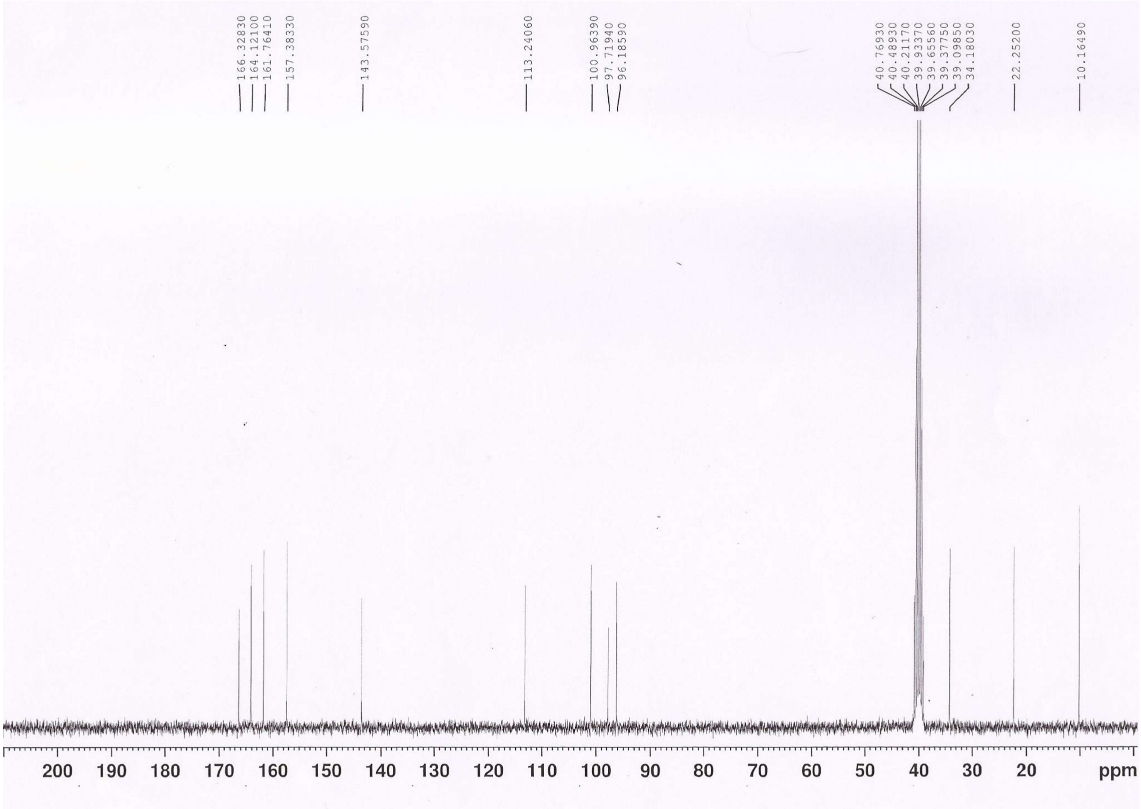

Figure S3. COSY spectrum of **1** (DMSO$_{d6}$, 300 MHz).

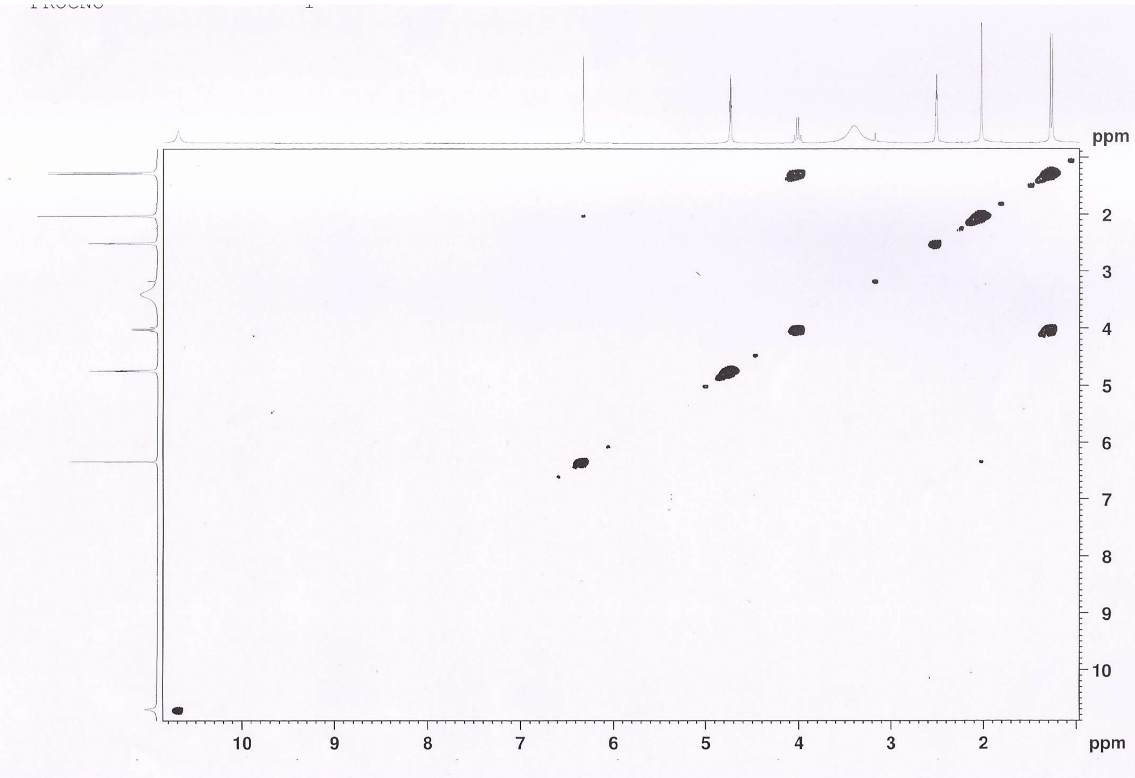

Figure S4. HSQC spectrum of **1** (DMSO$_{d6}$, 300 MHz).

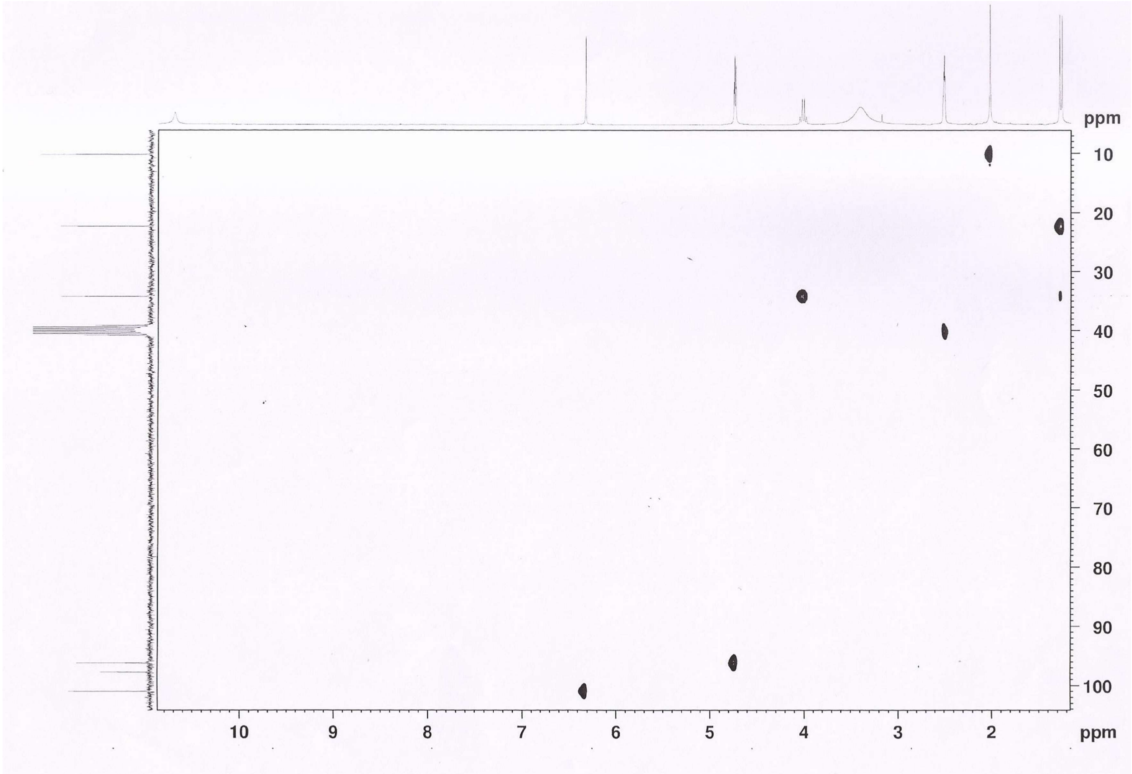

Figure S5. HMBC spectrum of **1** (DMSO$_{d6}$, 300 MHz).

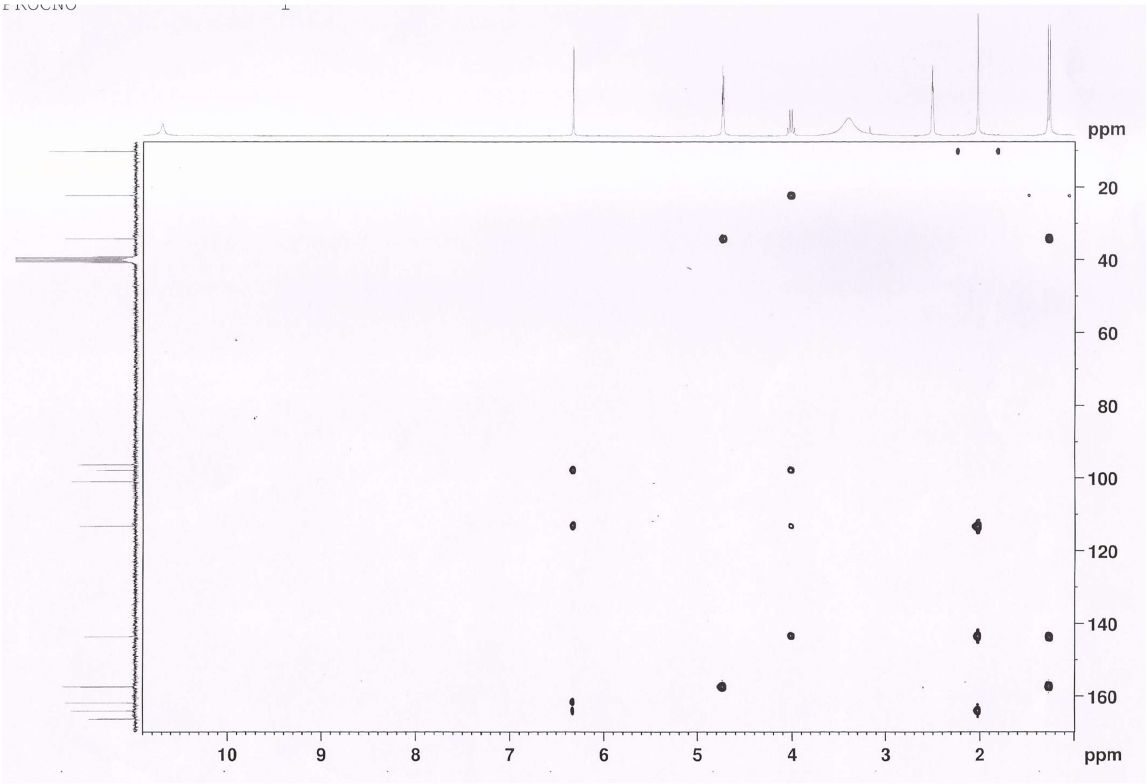

Figure S6. (+)-HRESIMS spectrum of **1**

Elemental Composition Report [MH]$^+$

Single Mass Analysis

Tolerance = 5.0 PPM / DBE: min = -1.5, max = 100.0

Element prediction: Off

Number of isotope peaks used for i-FIT = 3

Monoisotopic Mass, Even Electron Ions

13 formula(e) evaluated with 1 results within limits (up to 100 closest results for each mass)

Elements Used:

C: 12-12 H: 0-150 O: 0-30

Minimum:				-1.5				
Maximum:		5.0	5.0	100.0				
Mass	Calc. Mass	mDa	PPM	DBE	i-FIT	Norm	Conf(%)	Formula
221.0814	221.0814	0.0	0.0	6.5	515.2	n/a	n/a	C12 H13 O4

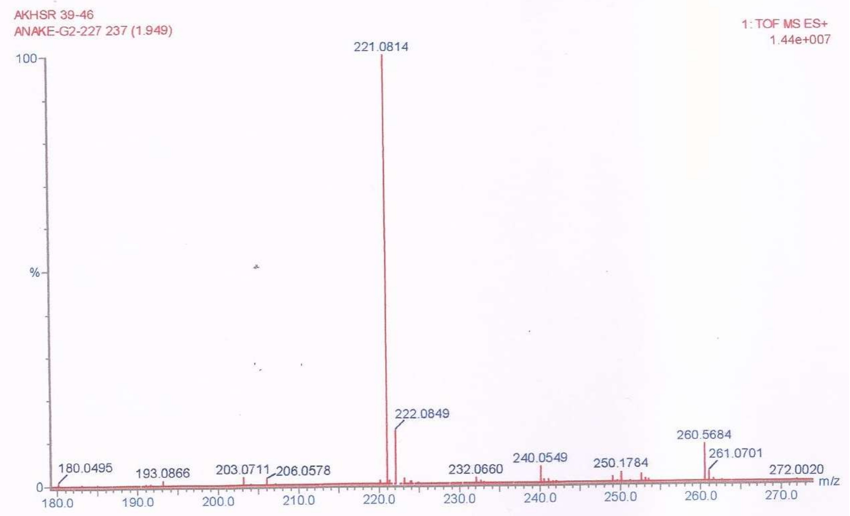

Figure S7. ^{1}H NMR spectrum of **2** ($CDCl_3$, 300 MHz).

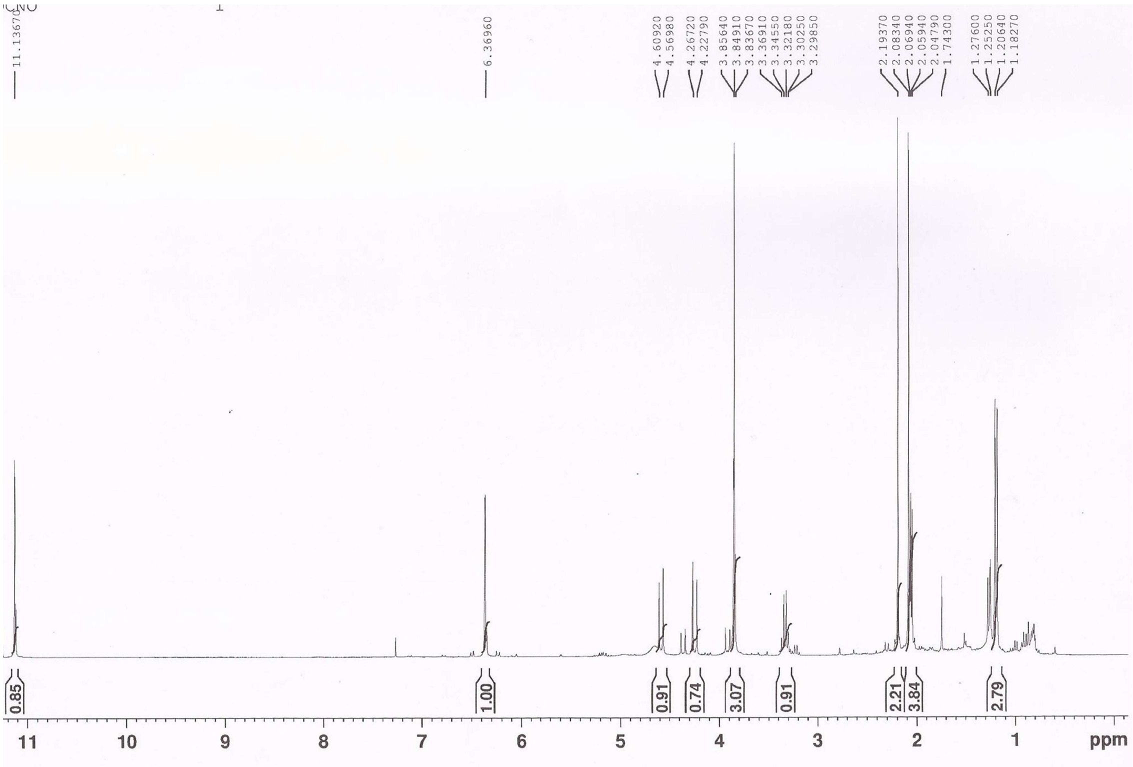

Figure S8. ^{13}C NMR spectrum of **2** ($CDCl_3$, 75 MHz).

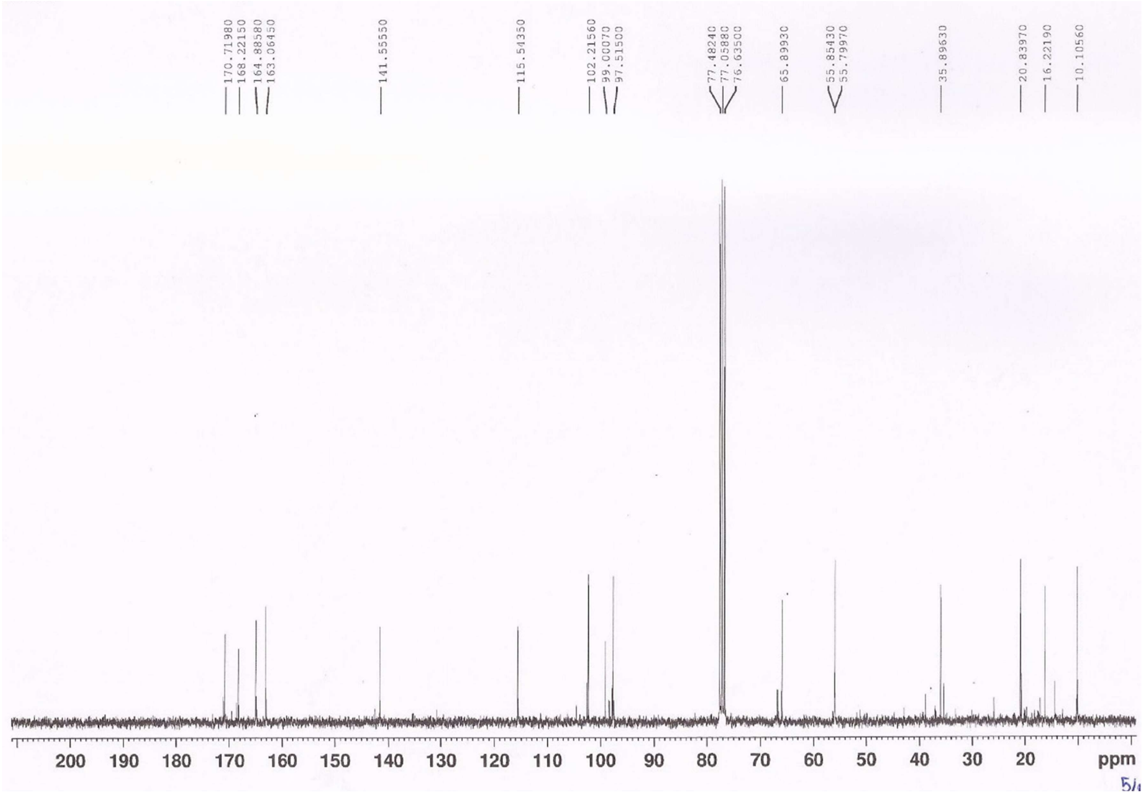

Figure S9. COSY spectrum of **2** ($CDCl_3$, 300 MHz).

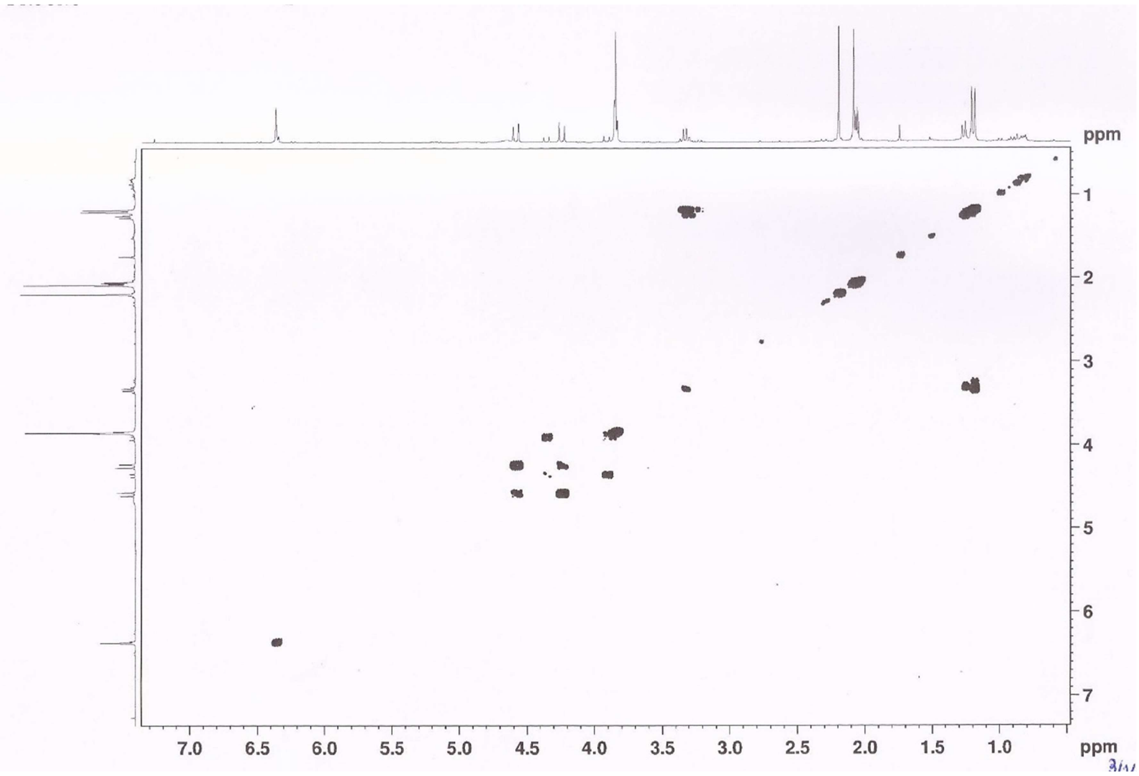

Figure S10. HSQC spectrum of **2** ($CDCl_3$, 300 MHz).

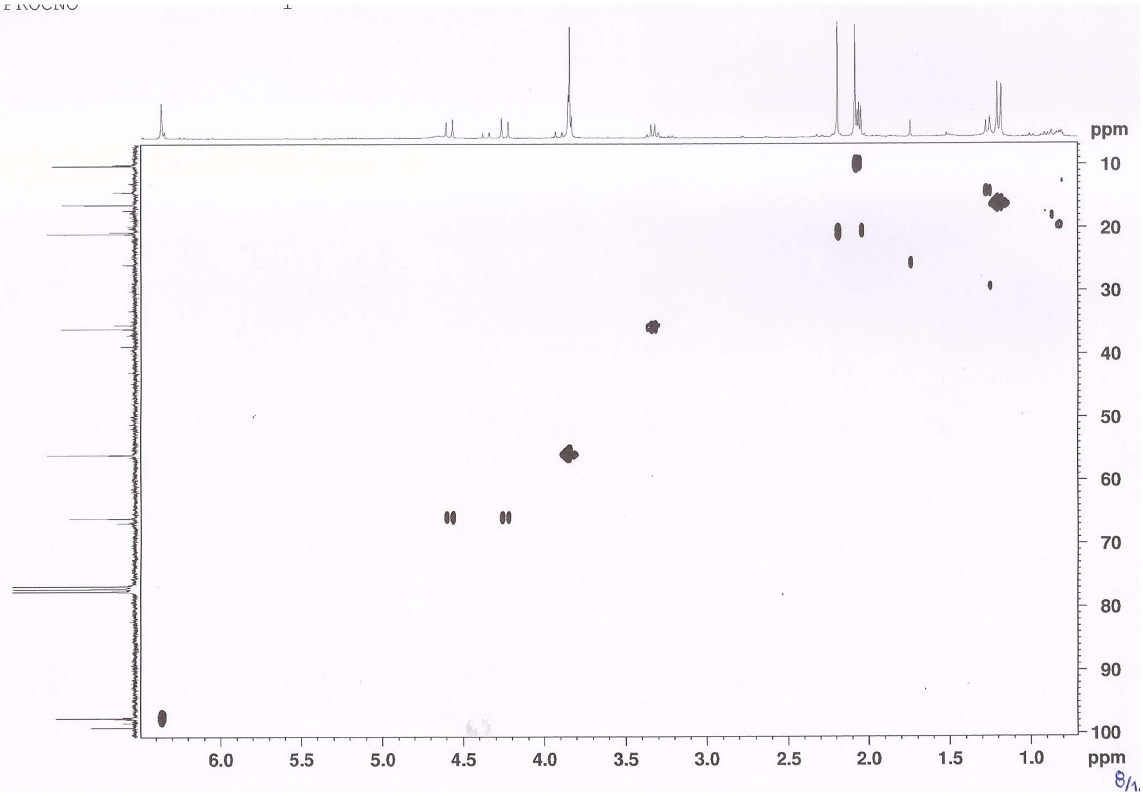

Figure S11. HMBC spectrum of **2** ($CDCl_3$, 300 MHz).

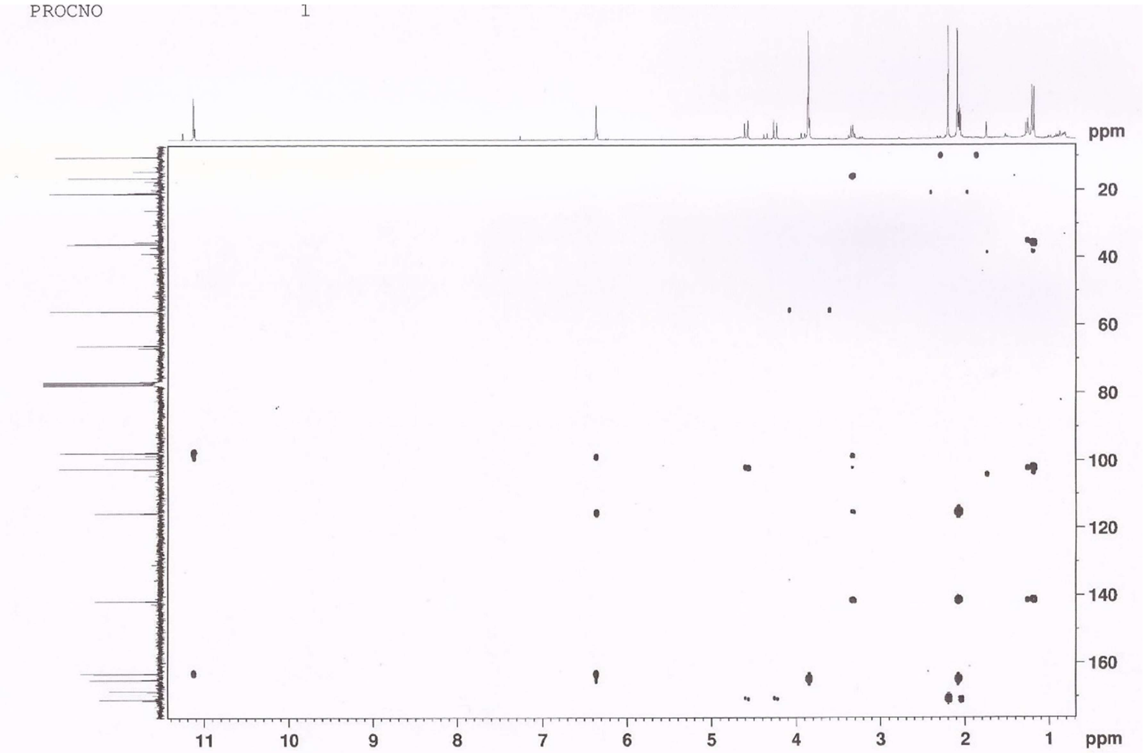

Figure S12. ROESY spectrum of **2** ($CDCl_3$, 300 MHz).

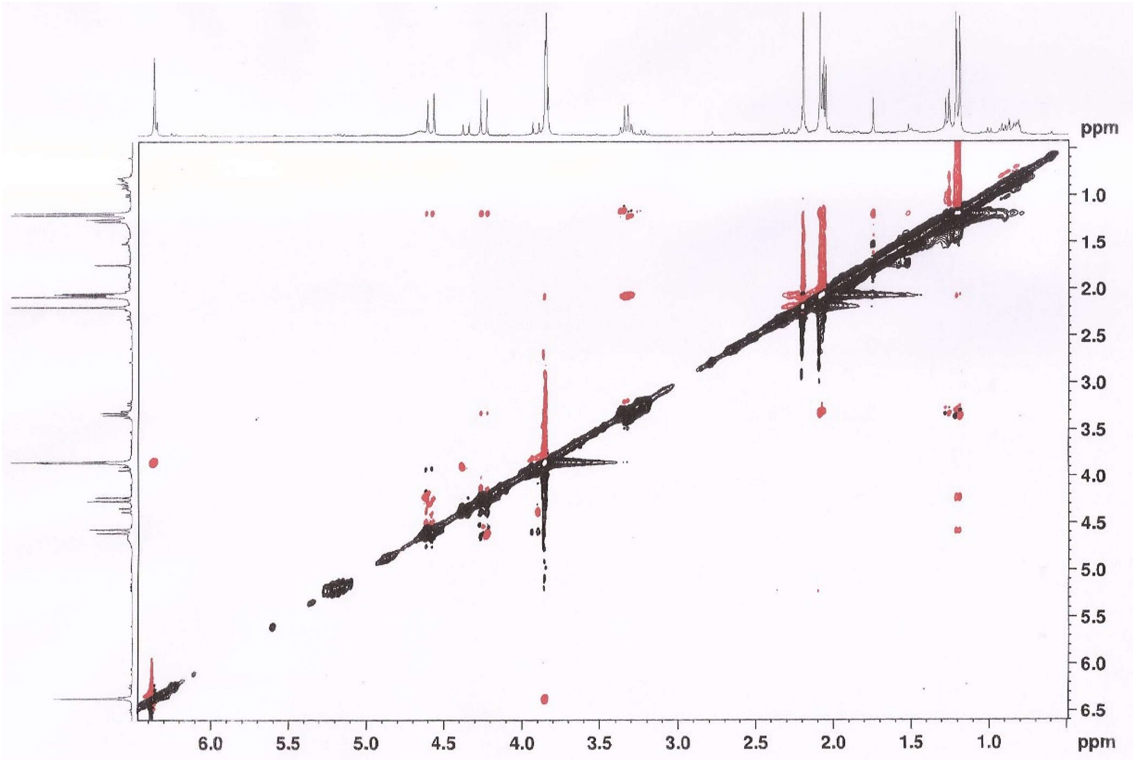

Figure S13. (+)-HRESIMS spectrum of **2.**

Elemental Composition Report [MH]+

Single Mass Analysis

Tolerance = 5.0 PPM / DBE: min = -1.5, max = 100.0

Element prediction: Off

Number of isotope peaks used for i-FIT = 3

Monoisotopic Mass, Even Electron Ions

20 formula(e) evaluated with 1 results within limits (up to 100 closest results for each mass)

Elements Used:

C: 15-15 H: 0-150 O: 0-30

Minimum:				-1.5				
Maximum:		5.0	5.0	100.0				
Mass	Calc. Mass	mDa	PPM	DBE	i-FIT	Norm	Conf(%)	Formula
311.1133	311.1131	0.2	0.6	6.5	592.6	n/a	n/a	C15 H19 O7

Elemental Composition Report [MNa]+

Single Mass Analysis

Tolerance = 5.0 PPM / DBE: min = -1.5, max = 100.0

Element prediction: Off

Number of isotope peaks used for i-FIT = 3

Monoisotopic Mass, Even Electron Ions

41 formula(e) evaluated with 1 results within limits (up to 100 closest results for each mass)

Elements Used:

C: 15-15 H: 0-150 O: 0-30 Na: 0-1

Minimum:				-1.5				
Maximum:		5.0	5.0	100.0				
Mass	Calc. Mass	mDa	PPM	DBE	i-FIT	Norm	Conf(%)	Formula
333.0948	333.0950	-0.2	-0.6	6.5	602.1	n/a	n/a	C15 H18 O7 Na

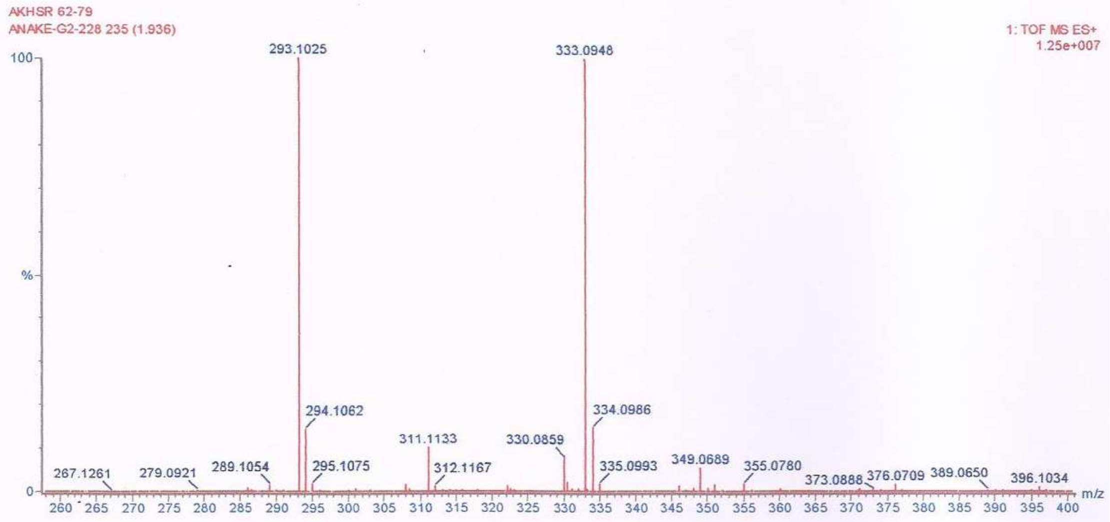

Figure S14. ^{1}H NMR spectrum of **3** ($CDCl_3$, 300 MHz).

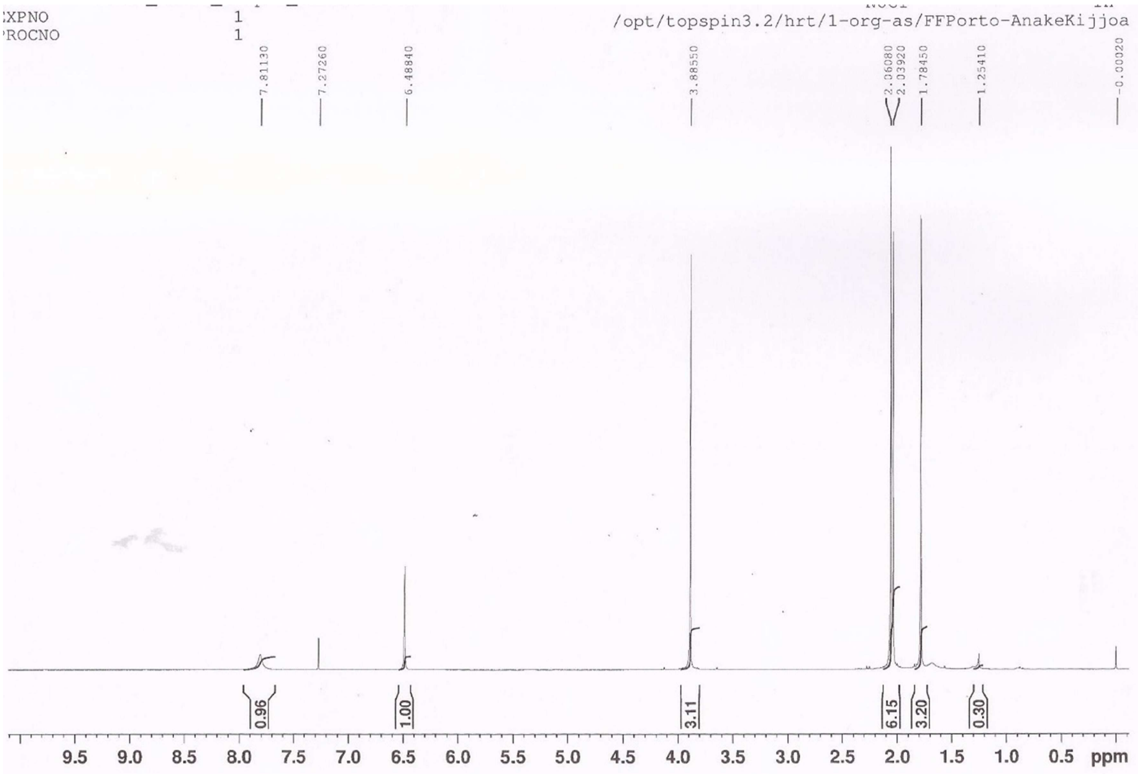

Figure S15. ^{13}C NMR spectrum of **3** ($CDCl_3$, 75 MHz).

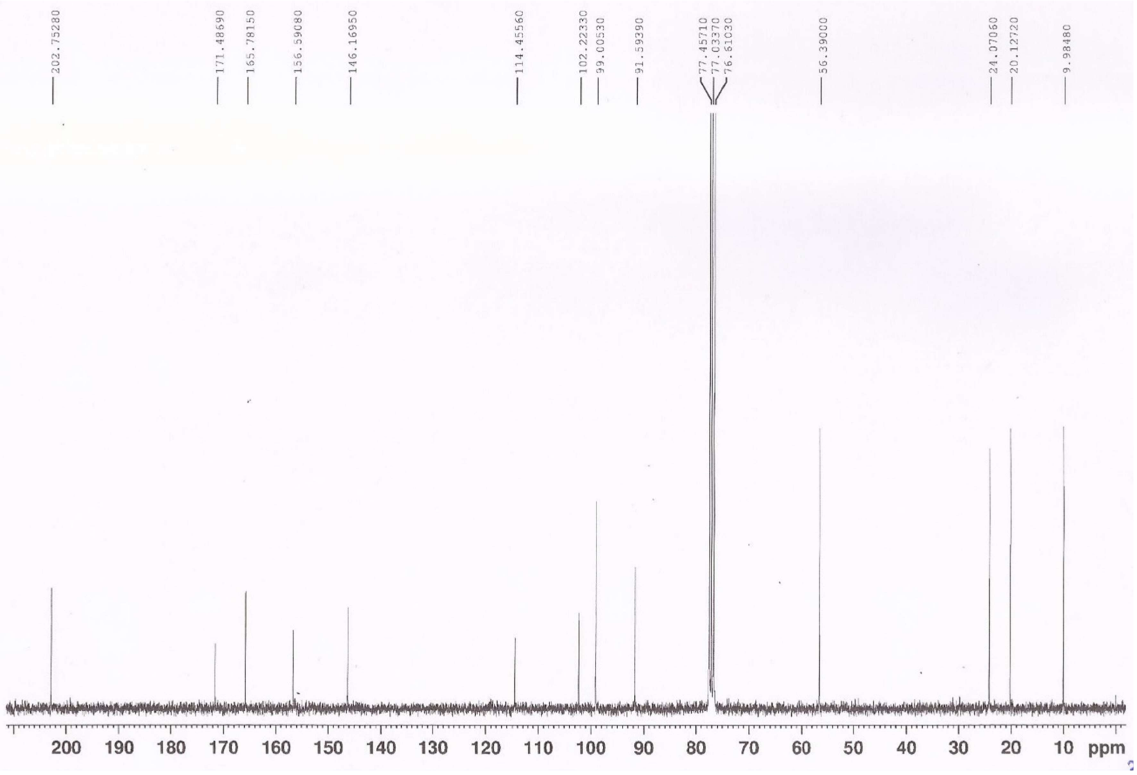

Figure S16. COSY spectrum of **3** ($CDCl_3$, 300 MHz).

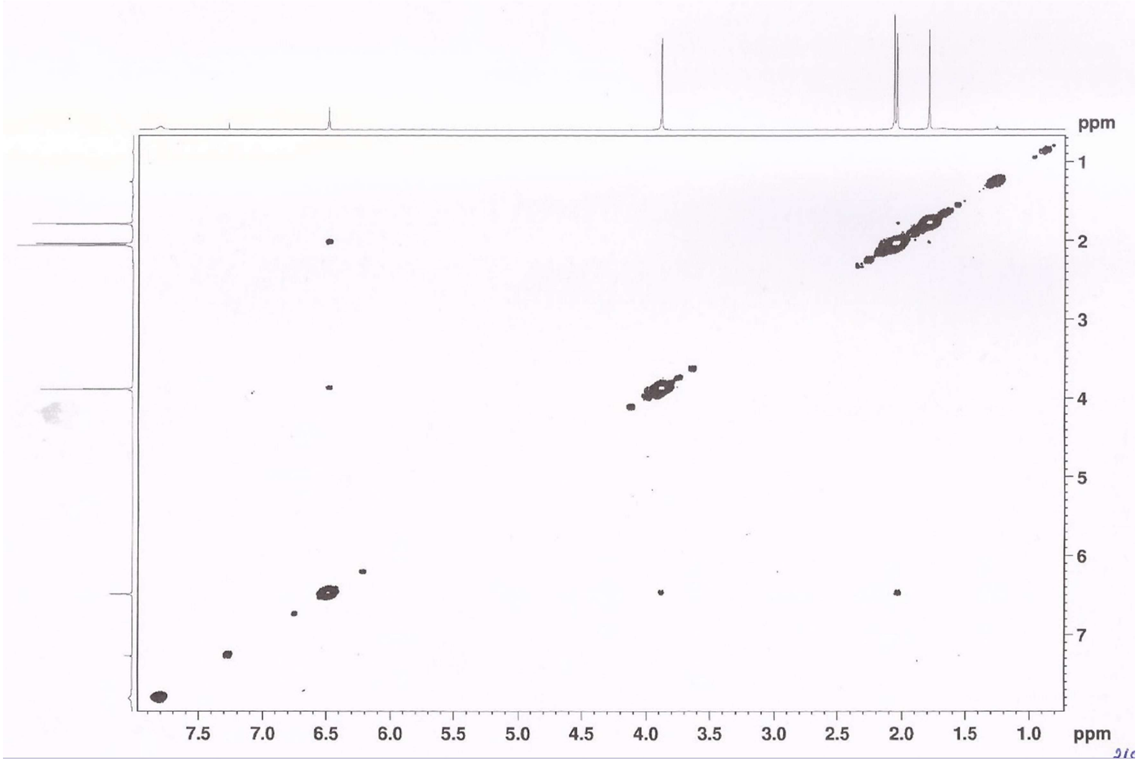

Figure S17. HSQC spectrum of **3** ($CDCl_3$, 300 MHz).

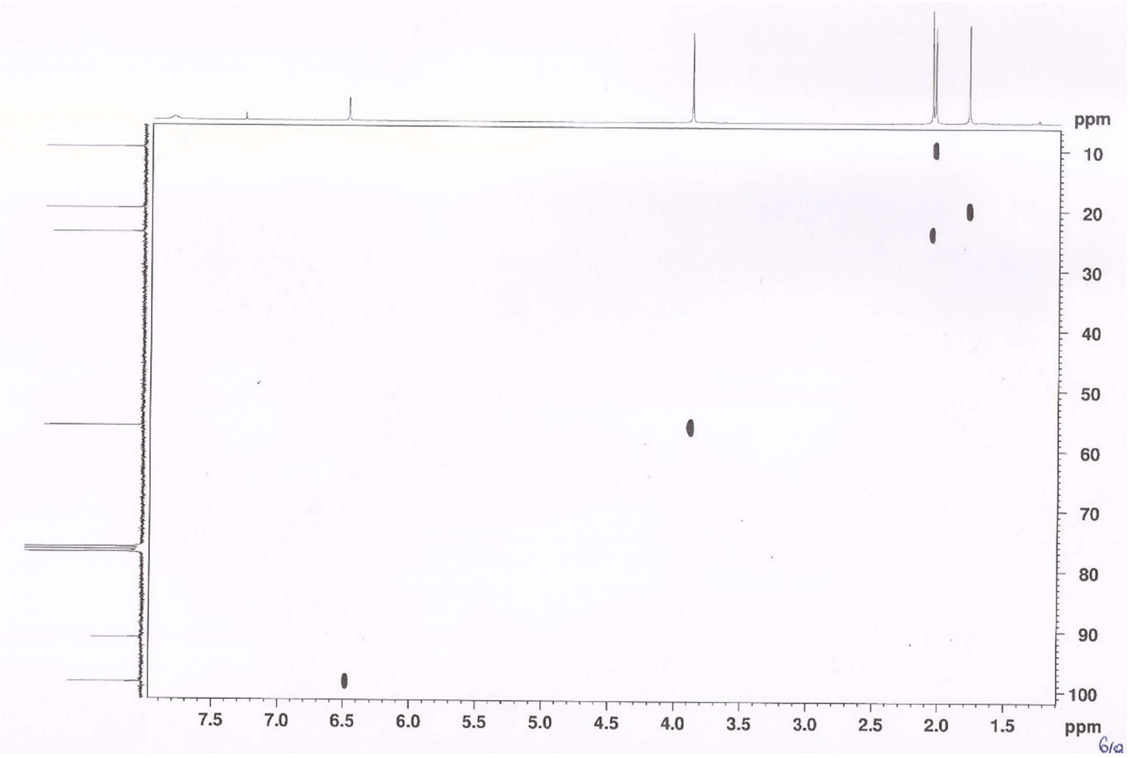

Figure S18. HMBC spectrum of **3** ($CDCl_3$, 300 MHz).

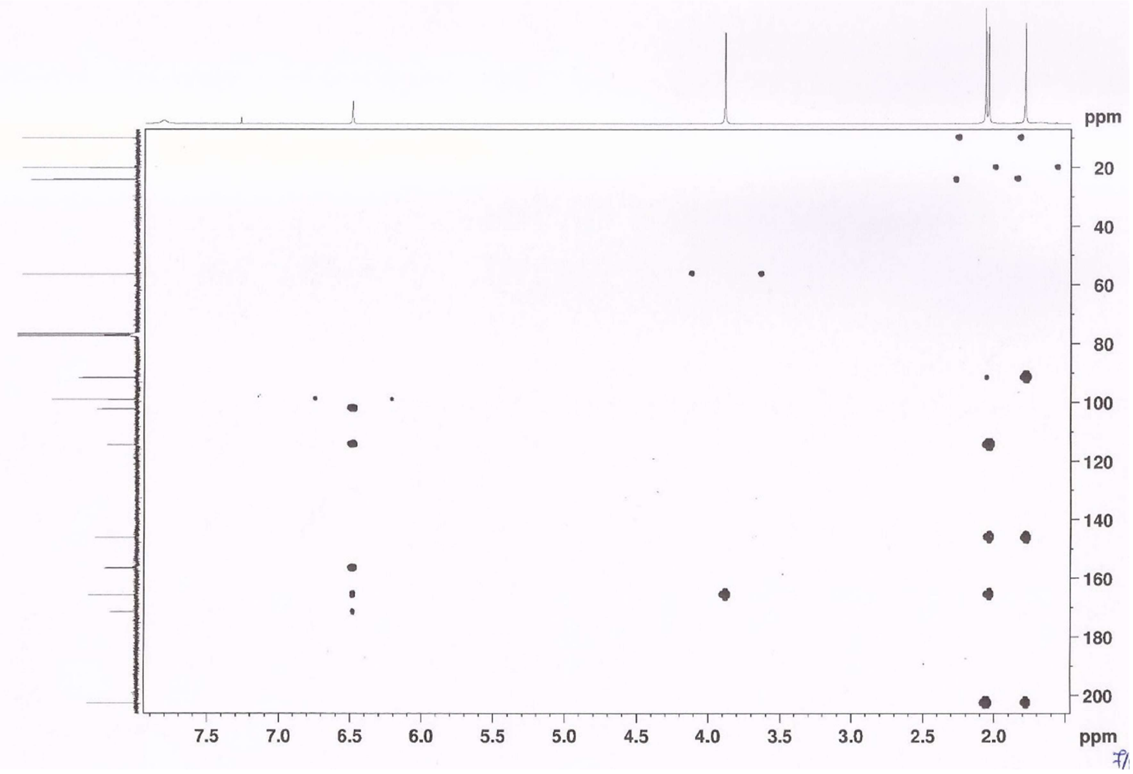

Figure S19. ^{1}H NMR spectrum of **4a** ($CDCl_3$, 300 MHz).

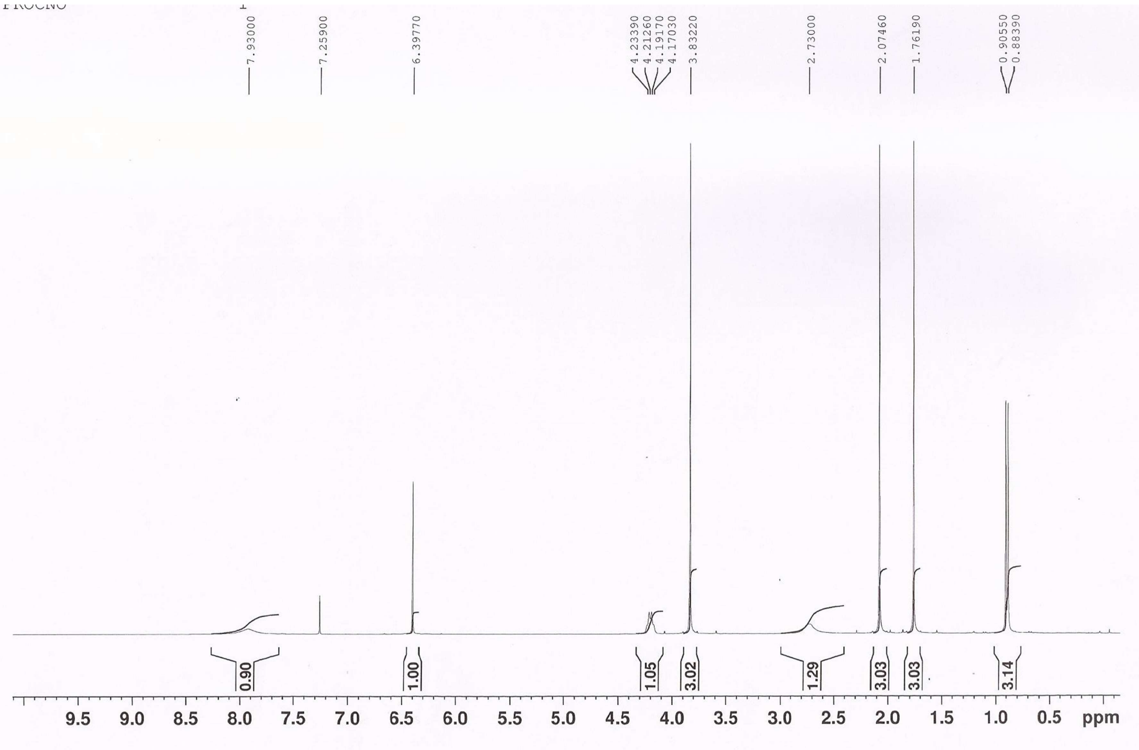

Figure S20. ^{13}C NMR spectrum of **4a** ($CDCl_3$,75 MHz).

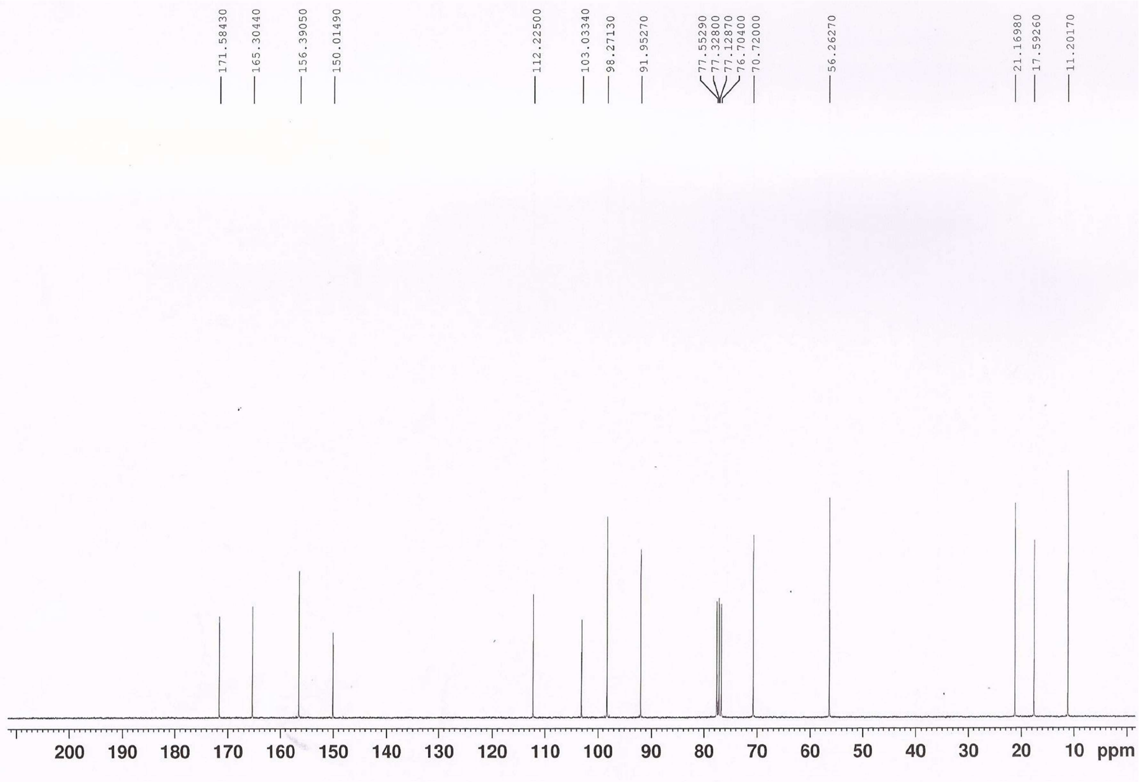

Figure S21. COSY spectrum of **4a** ($CDCl_3$, 300 MHz).

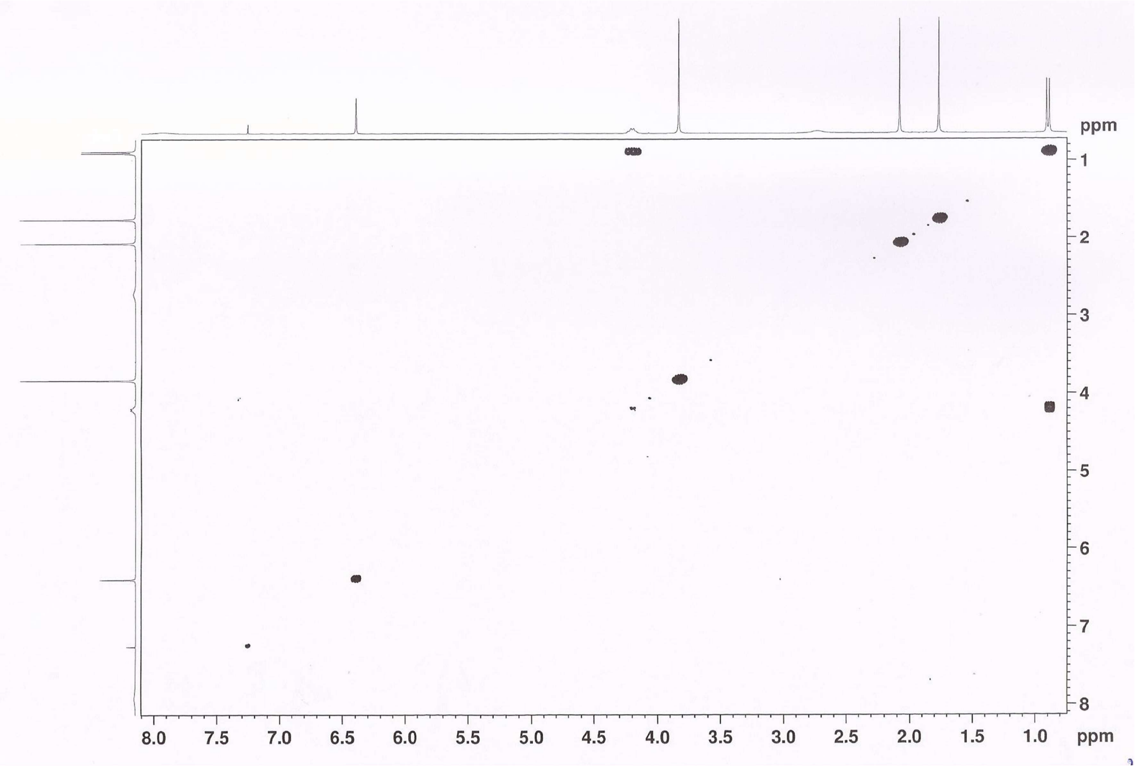

Figure S22. HSQC spectrum of **4a** ($CDCl_3$, 300 MHz).

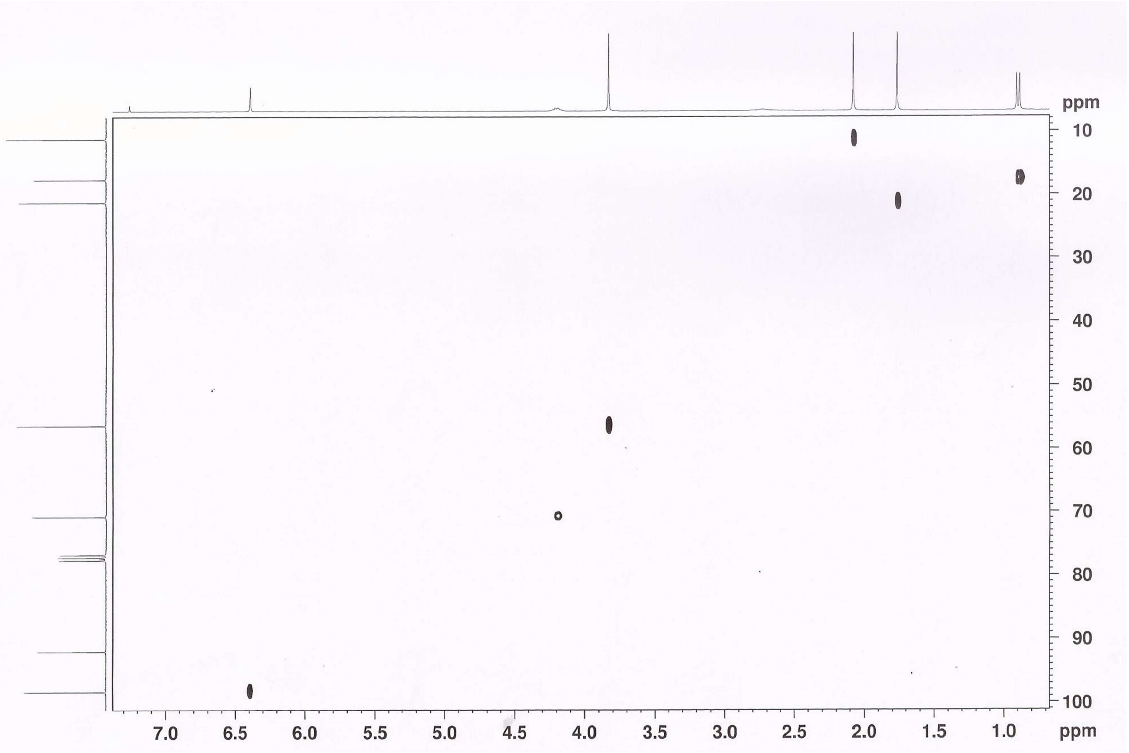

Figure S23. HMBC spectrum of **4a** ($CDCl_3$, 300 MHz).

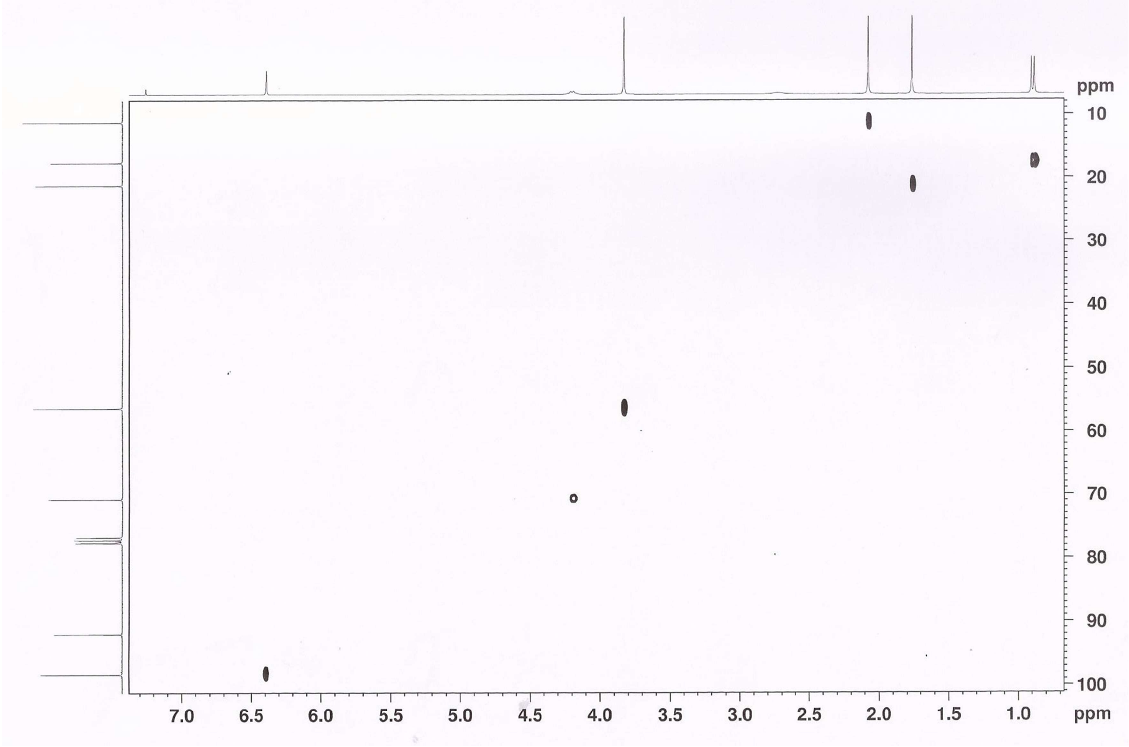

Figure S24. ^{1}H NMR spectrum of **4b** ($CDCl_3$, 300 MHz).

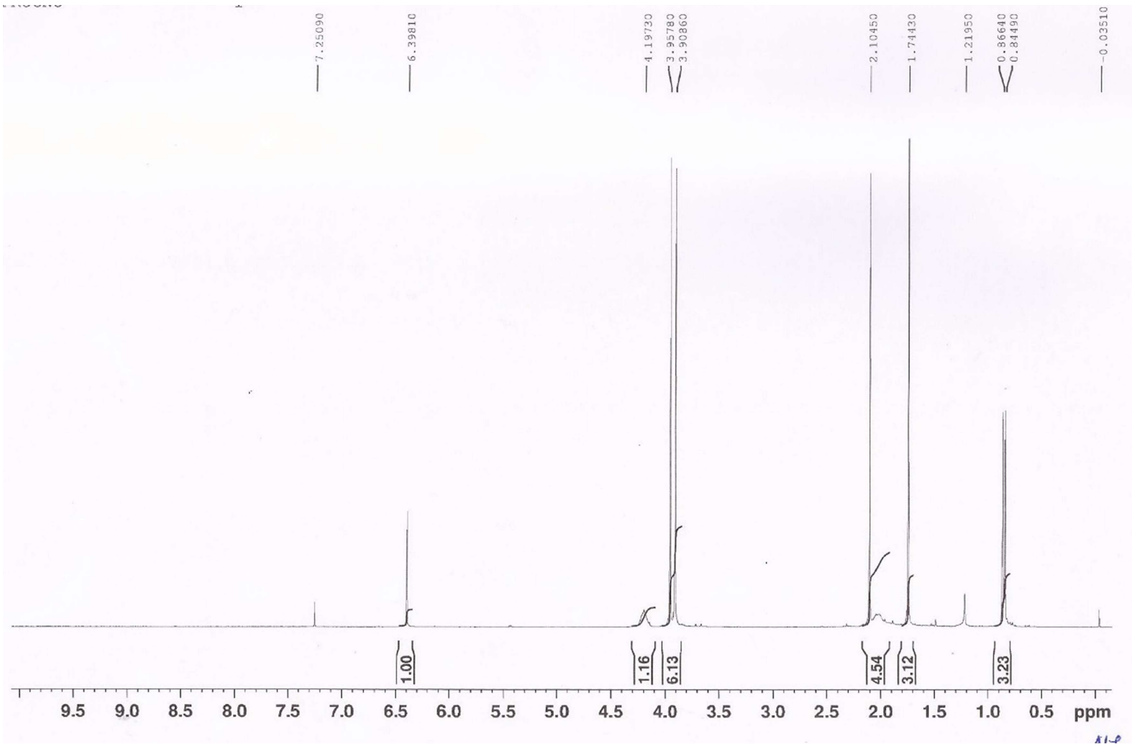

Figure S25. ^{13}C NMR spectrum of **4b** ($CDCl_3$, 75 MHz).

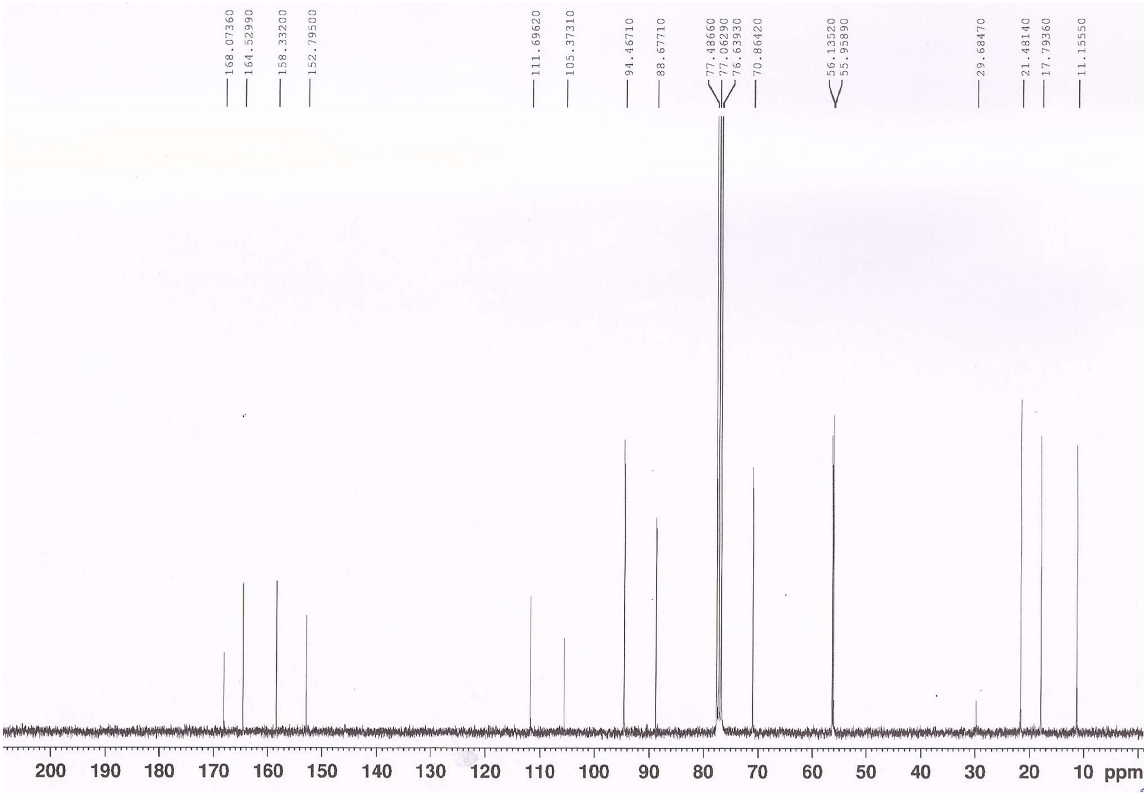

Figure S26. COSY spectrum of **4b** ($CDCl_3$, 300 MHz).

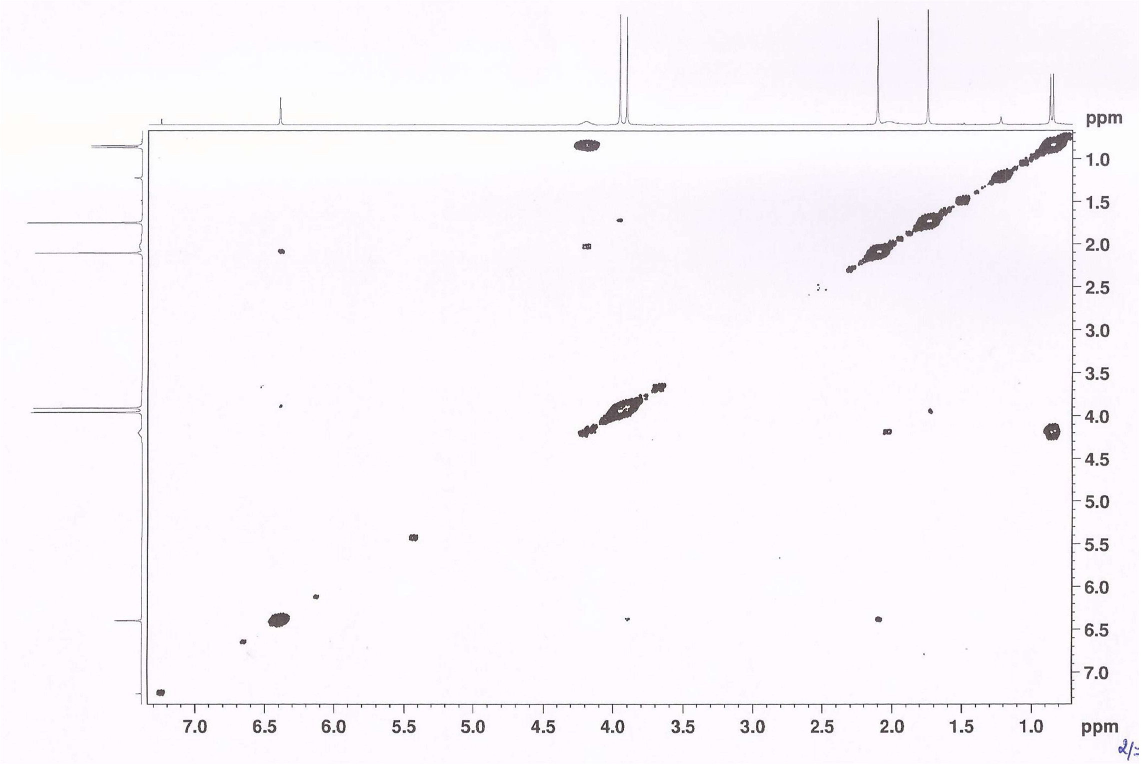

Figure S27. HSQC spectrum of **4b** ($CDCl_3$, 300 MHz).

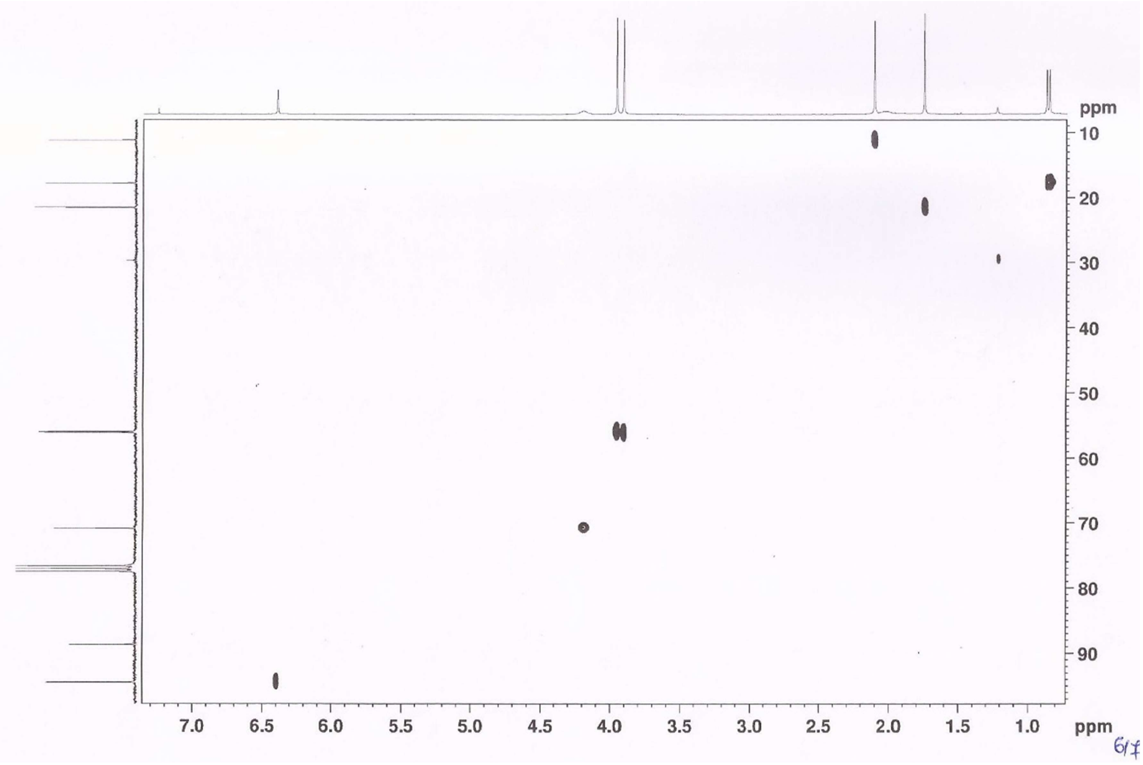

Figure S28. HMBC spectrum of **4b** ($CDCl_3$, 300 MHz).

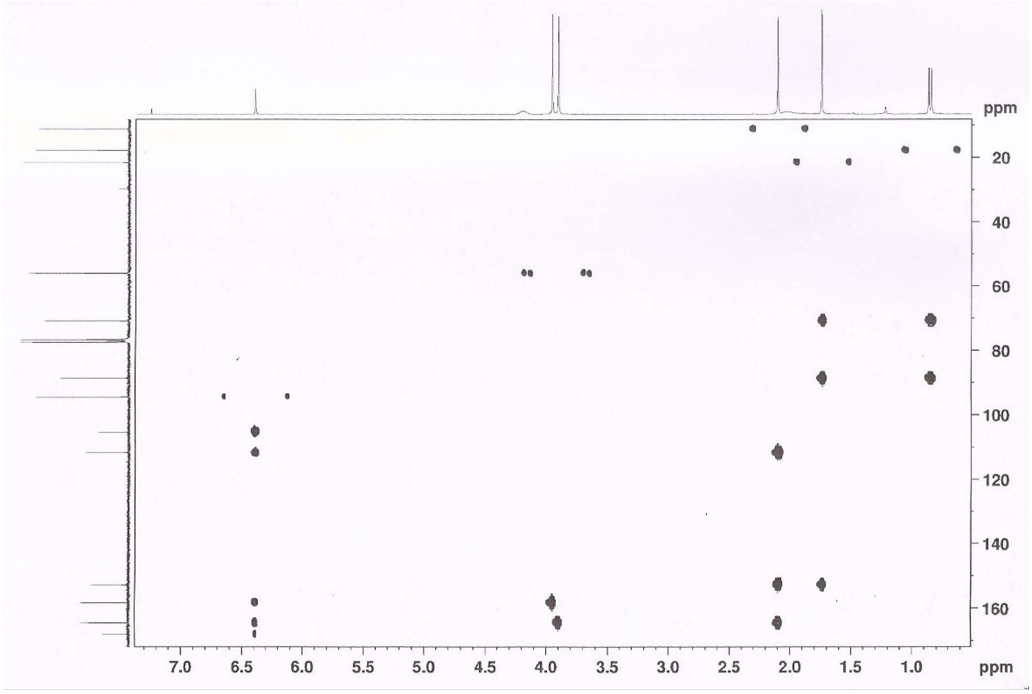

Figure S29. (+)-HRESIMS of **4b**

Elemental Composition Report [MH]+
Single Mass Analysis
Tolerance = 5.0 PPM / DBE: min = -1.5, max = 100.0
Element prediction: Off
Number of isotope peaks used for i-FIT = 3
Monoisotopic Mass, Even Electron Ions
17 formula(e) evaluated with 1 results within limits (up to 100 closest results for each mass)
Elements Used:
C: 14-14 H: 0-150 O: 0-30

Minimum:				-1.5				
Maximum:		5.0	5.0	100.0				
Mass	Calc. Mass	mDa	PPM	DBE	i-FIT	Norm	Conf(%)	Formula
267.1233	267.1232	0.1	0.4	5.5	845.2	n/a	n/a	C14 H19 O5

Elemental Composition Report [MNa]+
Single Mass Analysis
Tolerance = 5.0 PPM / DBE: min = -1.5, max = 100.0
Element prediction: Off
Number of isotope peaks used for i-FIT = 3
Monoisotopic Mass, Even Electron Ions
36 formula(e) evaluated with 1 results within limits (up to 100 closest results for each mass)
Elements Used:
C: 14-14 H: 0-150 O: 0-30 Na: 0-1

Minimum:				-1.5				
Maximum:		5.0	5.0	100.0				
Mass	Calc. Mass	mDa	PPM	DBE	i-FIT	Norm	Conf(%)	Formula
289.1053	289.1052	0.1	0.3	5.5	662.1	n/a	n/a	C14 H18 O5 Na

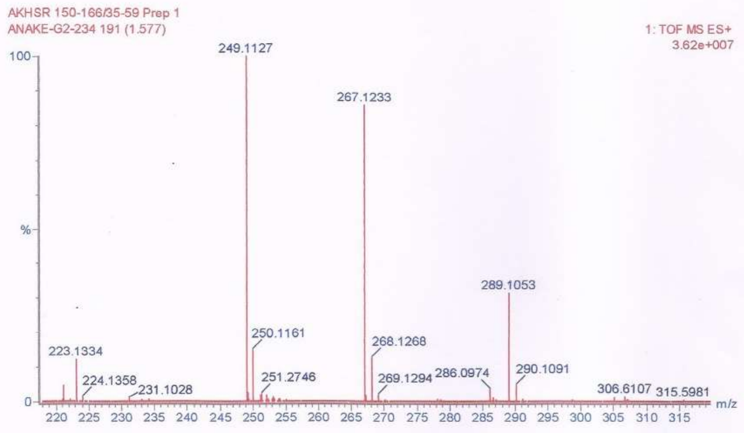

Figure S30. ^{1}H NMR spectrum of **5** ($CDCl_3$, 300 MHz).

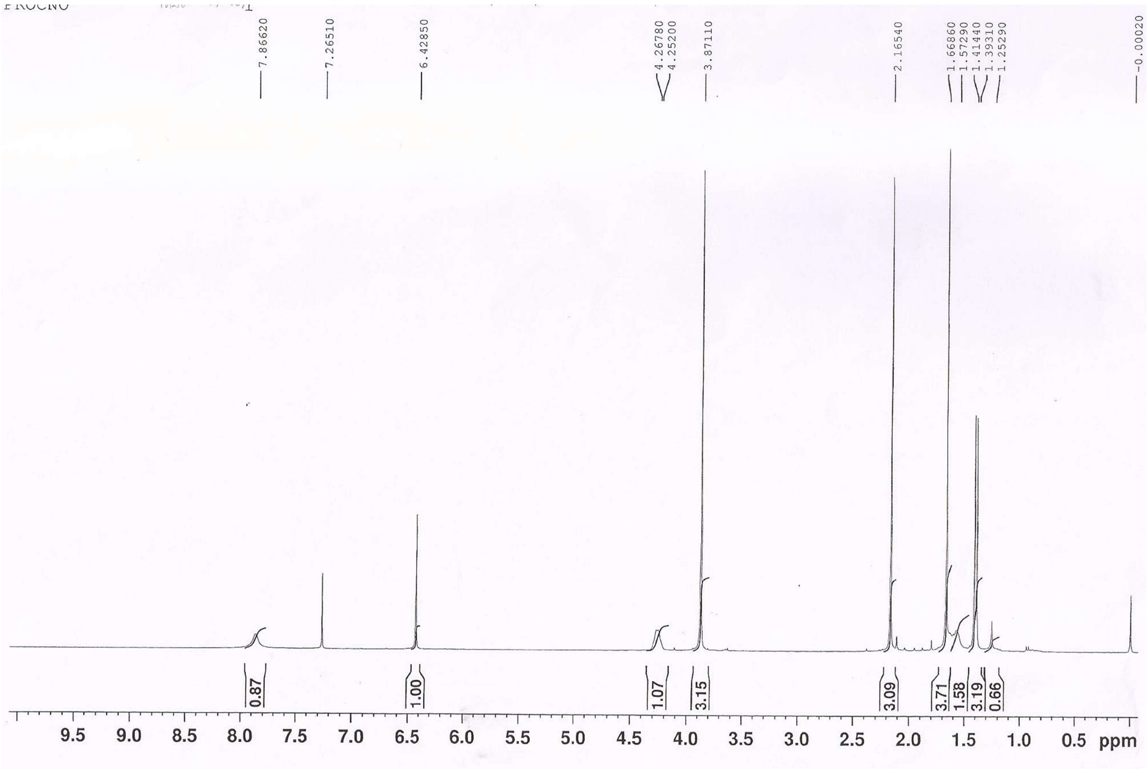

Figure S31. ^{13}C NMR spectrum of **5** ($CDCl_3$,75 MHz).

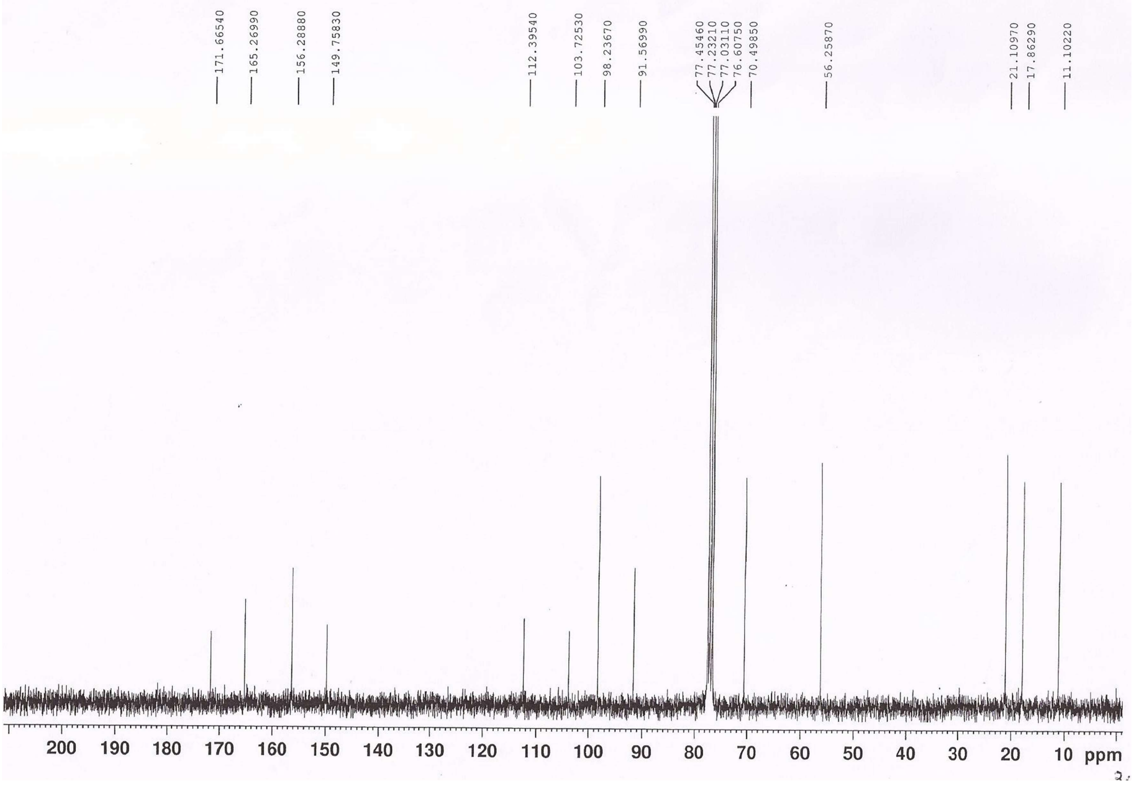

Figure S32. COSY spectrum of **5** ($CDCl_3$, 300 MHz).

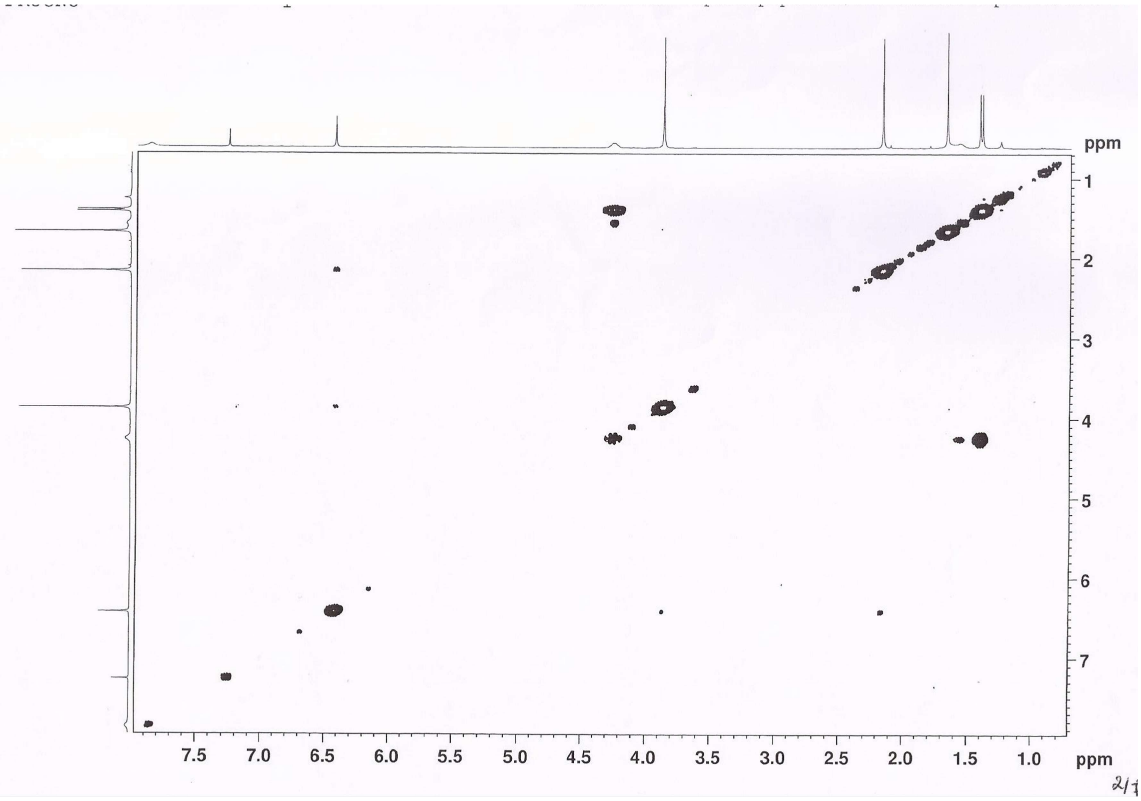

Figure S33. HSQC spectrum of **5** ($CDCl_3$, 300 MHz).

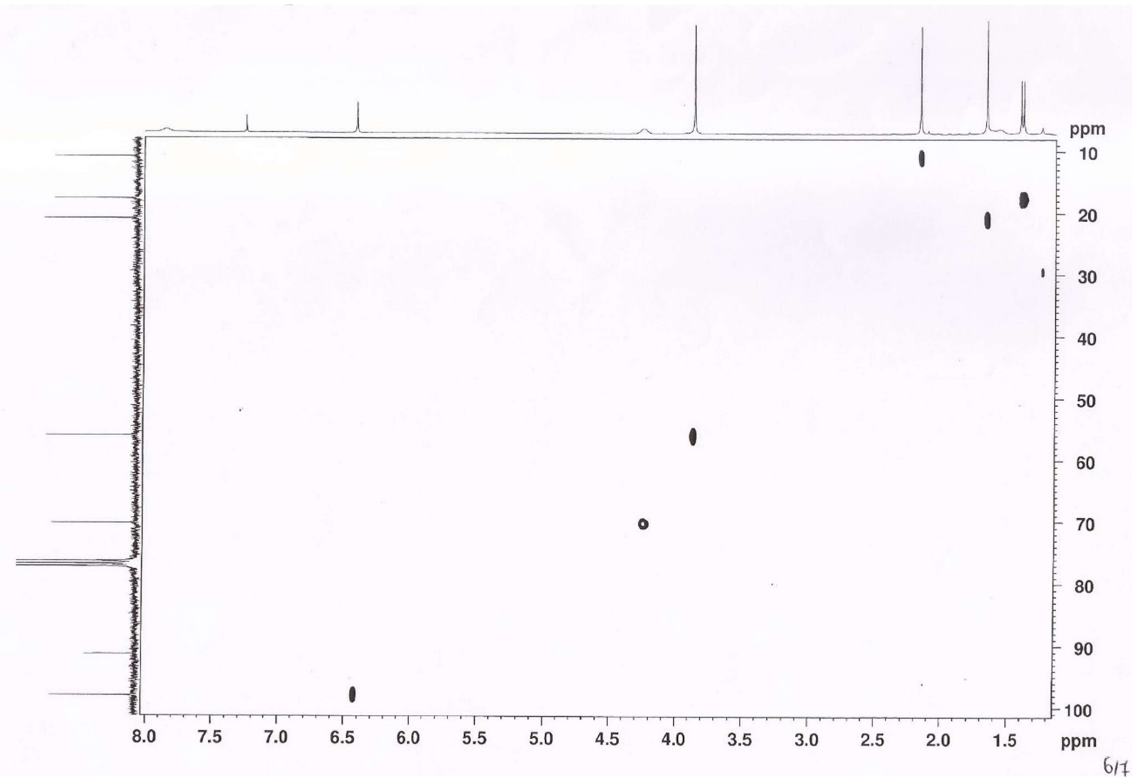

Figure S34. HMBC spectrum of **5** ($CDCl_3$, 300 MHz).

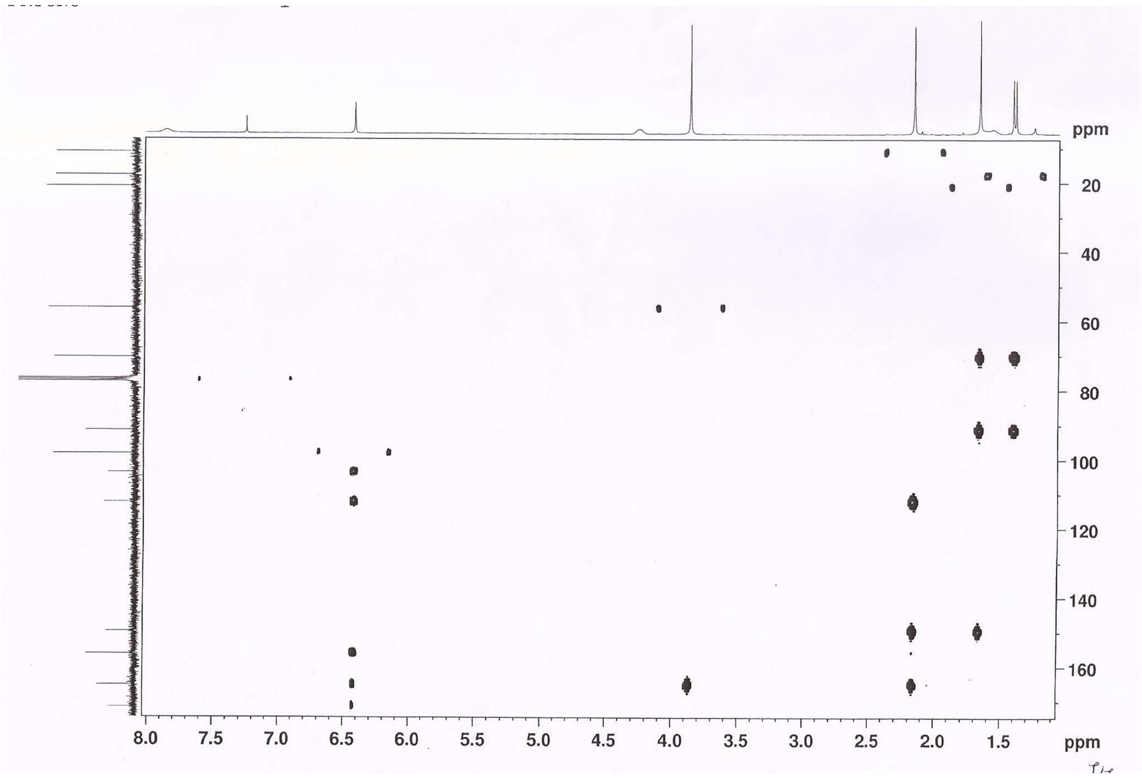

Figure S35. (+)-HRESIMS of **5.**

Elemental Composition Report [MH]+

Single Mass Analysis
Tolerance = 5.0 PPM / DBE: min = -1.5, max = 100.0
Element prediction: Off
Number of isotope peaks used for i-FIT = 3

Monoisotopic Mass, Even Electron Ions
16 formula(e) evaluated with 1 results within limits (up to 100 closest results for each mass)
Elements Used:
C: 13-13 H: 0-150 O: 0-30

Minimum:				-1.5				
Maximum:		5.0	5.0	100.0				
Mass	Calc. Mass	mDa	PPM	DBE	i-FIT	Norm	Conf(%)	Formula
253.1077	253.1076	0.1	0.4	5.5	832.1	n/a	n/a	C13 H17 O5

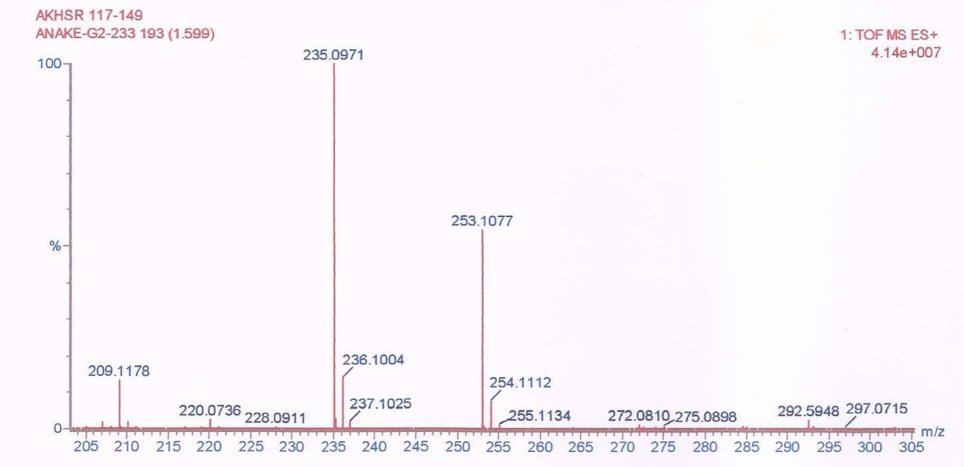

Figure S36. ^{1}H NMR spectrum of **6** (DMSO$_{d6}$, 300 MHz).

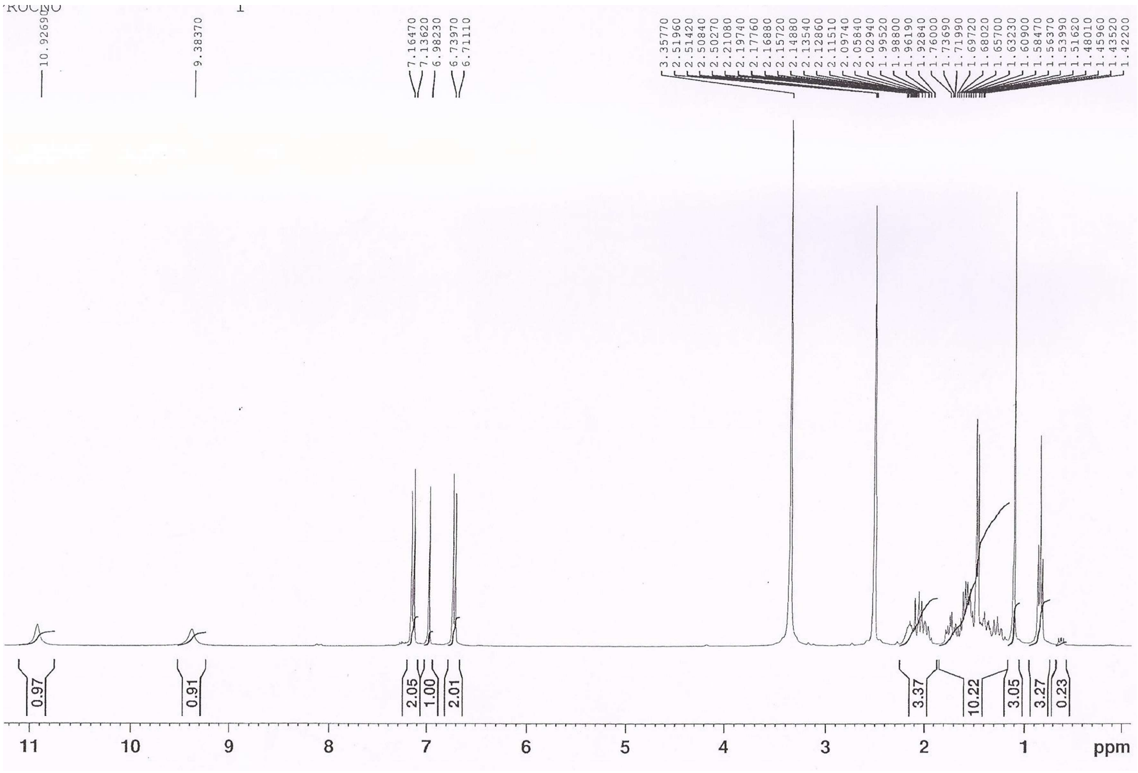

Figure S37. ^{13}C NMR spectrum of **6** (DMSO$_{d6}$, 75 MHz).

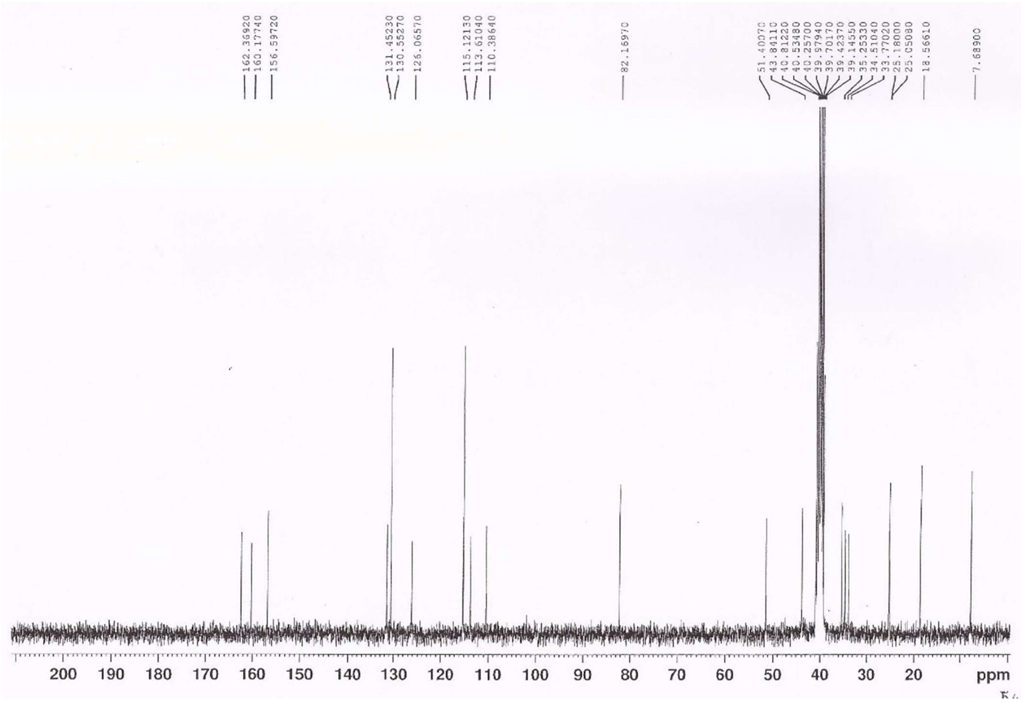

Figure S38. COSY spectrum of **6** (DMSO$_{d6}$, 300 MHz).

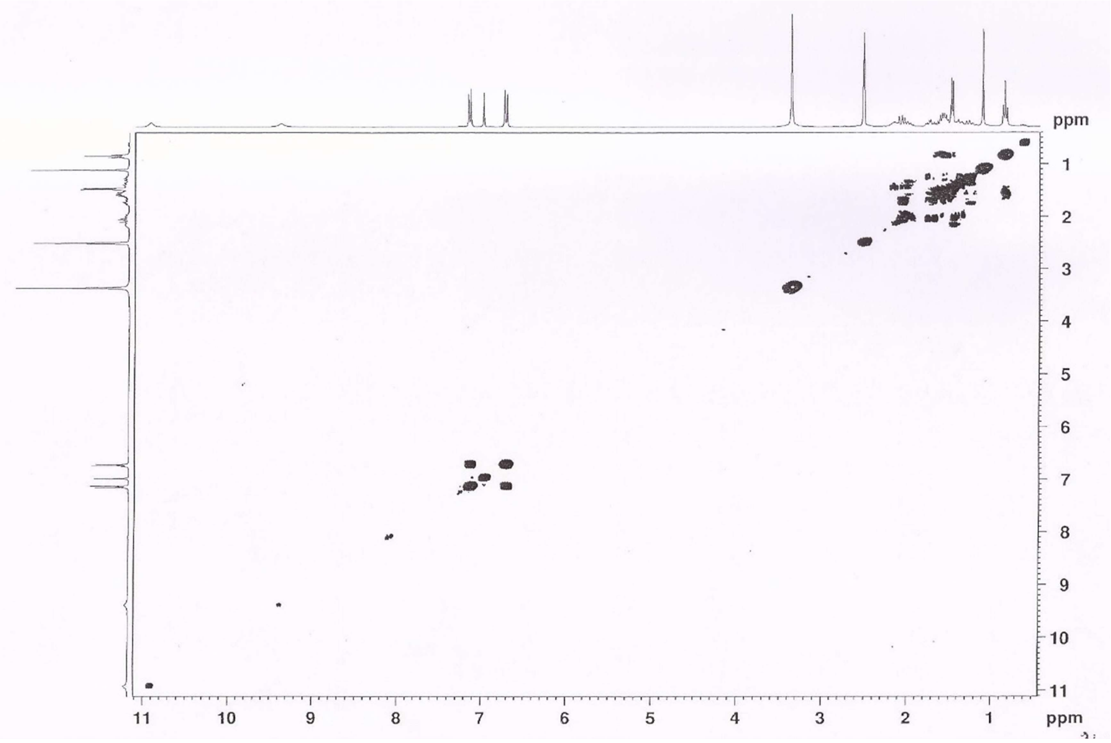

Figure S39. HSQC spectrum of **6** (DMSO$_{d6}$, 300 MHz).

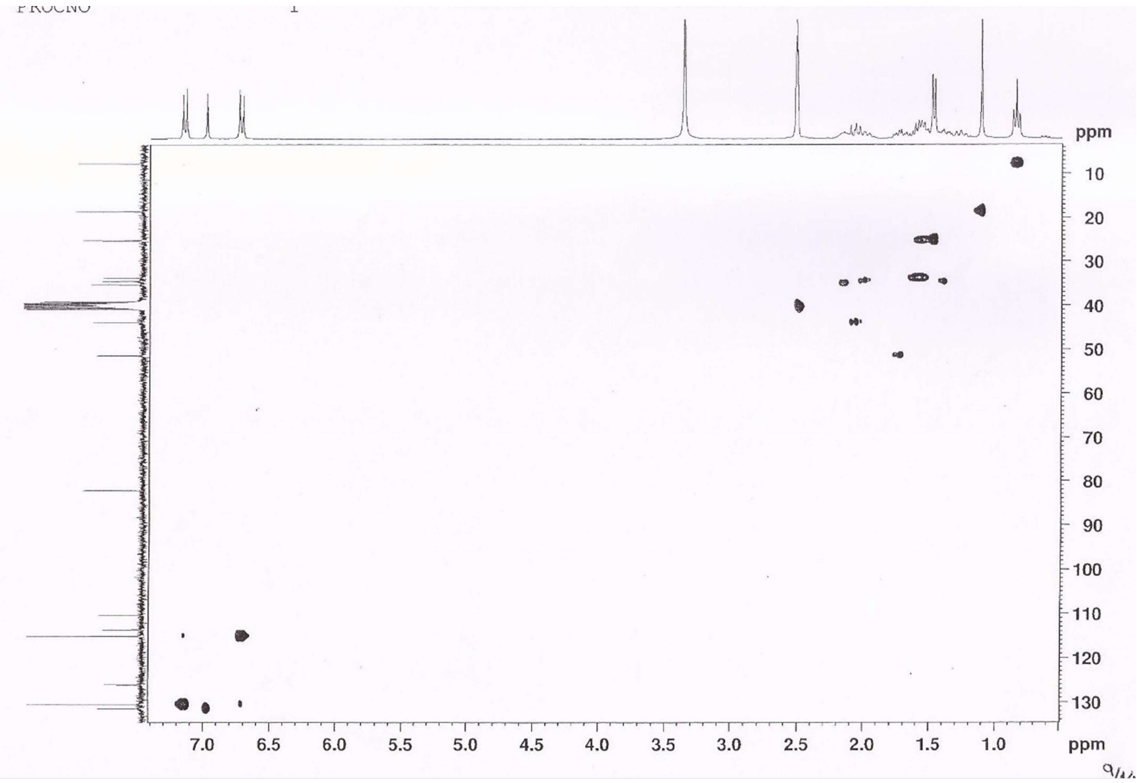

Figure S40. HMBC spectrum of **6** (DMSO$_{d6}$, 300 MHz).

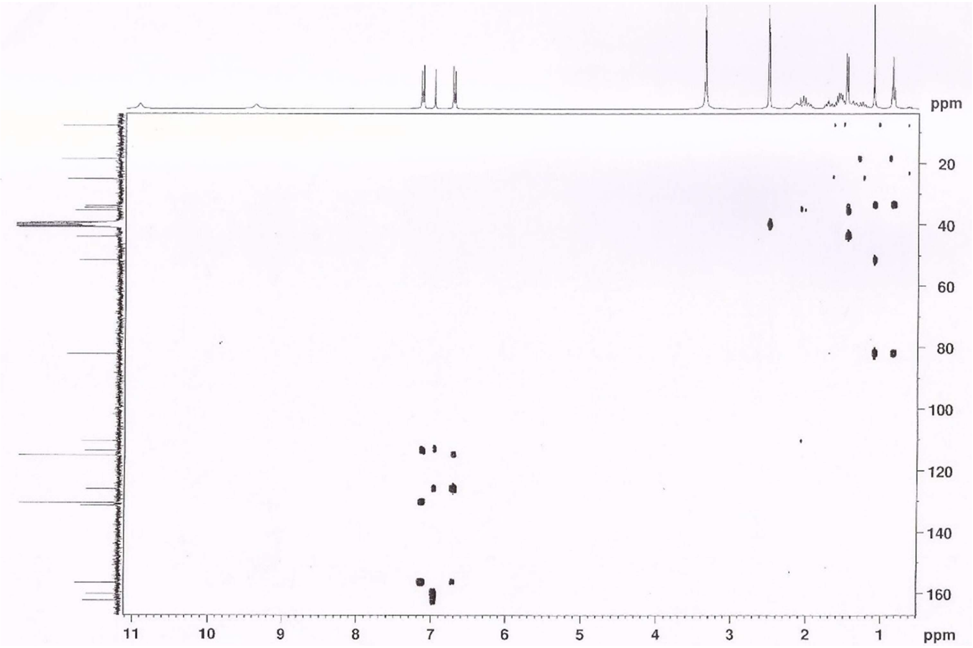

Figure S41. (+)-HRESIMS of **6.**

Match for $C_{21}H_{25}NO_3$ (No match for $C_{21}H_{26}O_3$)

Elemental Composition Report [MH]$^+$
Single Mass Analysis
Tolerance = 5.0 PPM / DBE: min = -1.5, max = 100.0
Element prediction: Off
Number of isotope peaks used for i-FIT = 3
Monoisotopic Mass, Even Electron Ions
372 formula(e) evaluated with 1 results within limits (up to 100 closest results for each mass)
Elements Used:
C: 18-25 H: 0-150 N: 0-30 O: 0-30

Minimum:				-1.5				
Maximum:		5.0	5.0	100.0				
Mass	Calc. Mass	mDa	PPM	DBE	i-FIT	Norm	Conf(%)	Formula
340.1913	340.1913	0.0	0.0	9.5	991.8	n/a	n/a	C21 H26 N O3

Elemental Composition Report [MNa]$^+$
Single Mass Analysis
Tolerance = 5.0 PPM / DBE: min = -1.5, max = 100.0
Element prediction: Off
Number of isotope peaks used for i-FIT = 3
Monoisotopic Mass, Even Electron Ions
592 formula(e) evaluated with 1 results within limits (up to 100 closest results for each mass)
Elements Used:
C: 21-21 H: 0-150 N: 0-30 O: 0-30 Na: 0-1

Minimum:				-1.5				
Maximum:		5.0	5.0	100.0				
Mass	Calc. Mass	mDa	PPM	DBE	i-FIT	Norm	Conf(%)	Formula
362.1734	362.1732	0.2	0.6	9.5	901.3	n/a	n/a	C21 H25 N O3 Na

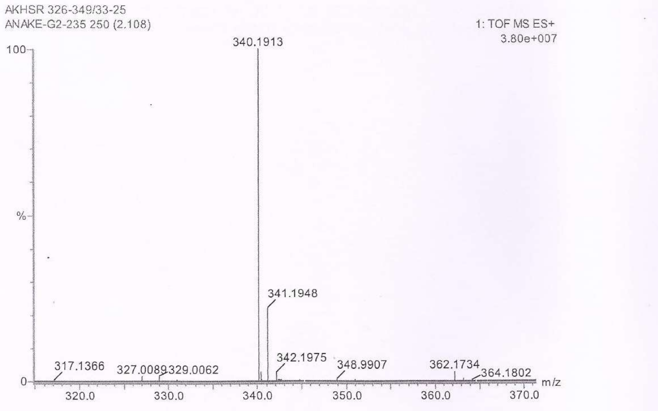

Figure S42. ^{1}H NMR spectrum of **7** ($CDCl_3$, 300 MHz).

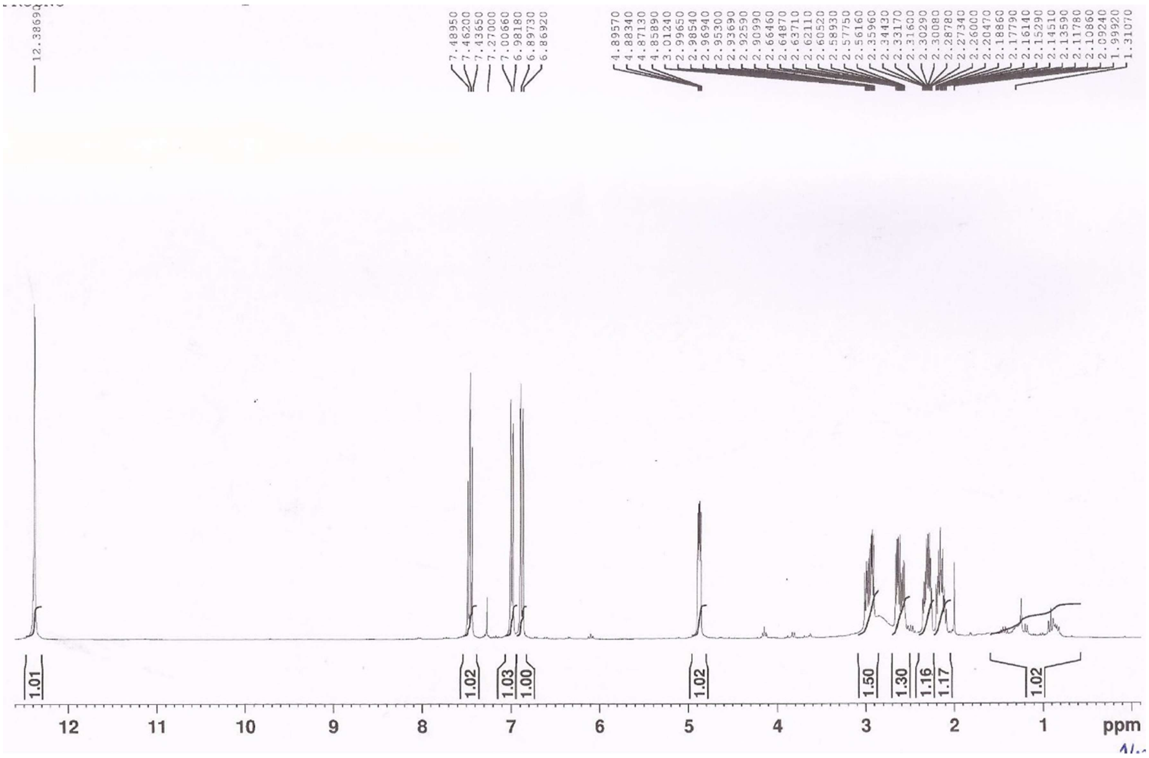

Figure S43. ^{13}C NMR spectrum of **7** ($CDCl_3$, 75 MHz).

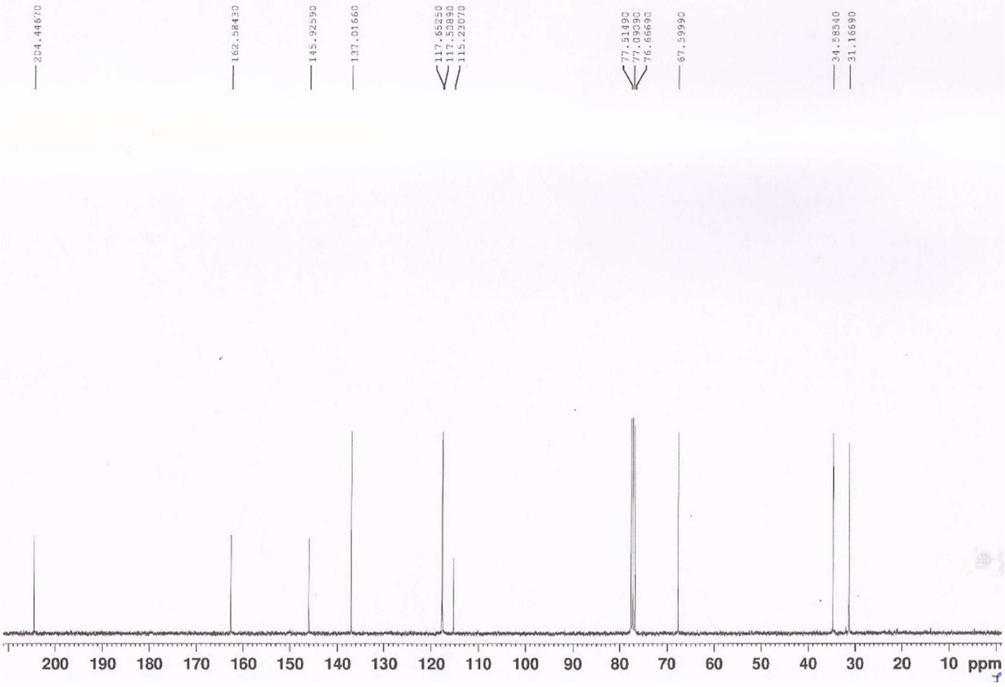

Figure S44. COSY spectrum of **7** ($CDCl_3$, 300 MHz).

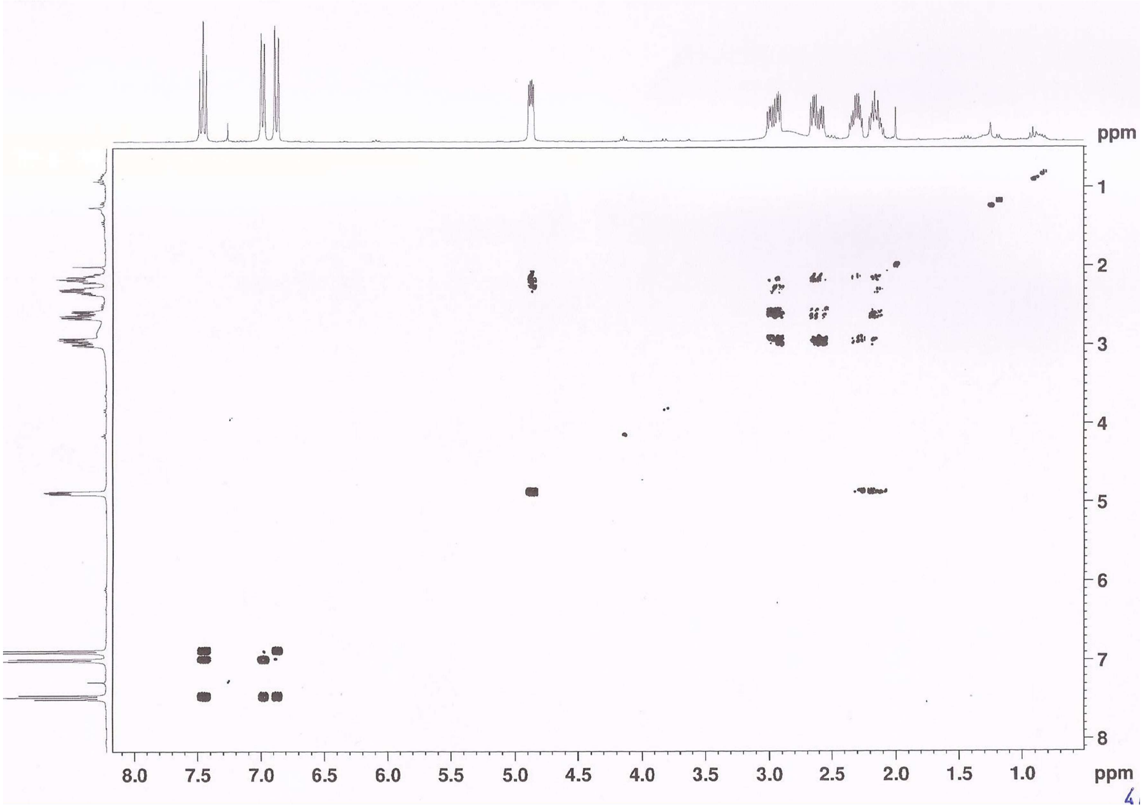

Figure S45. HSQC spectrum of **7** ($CDCl_3$, 300 MHz).

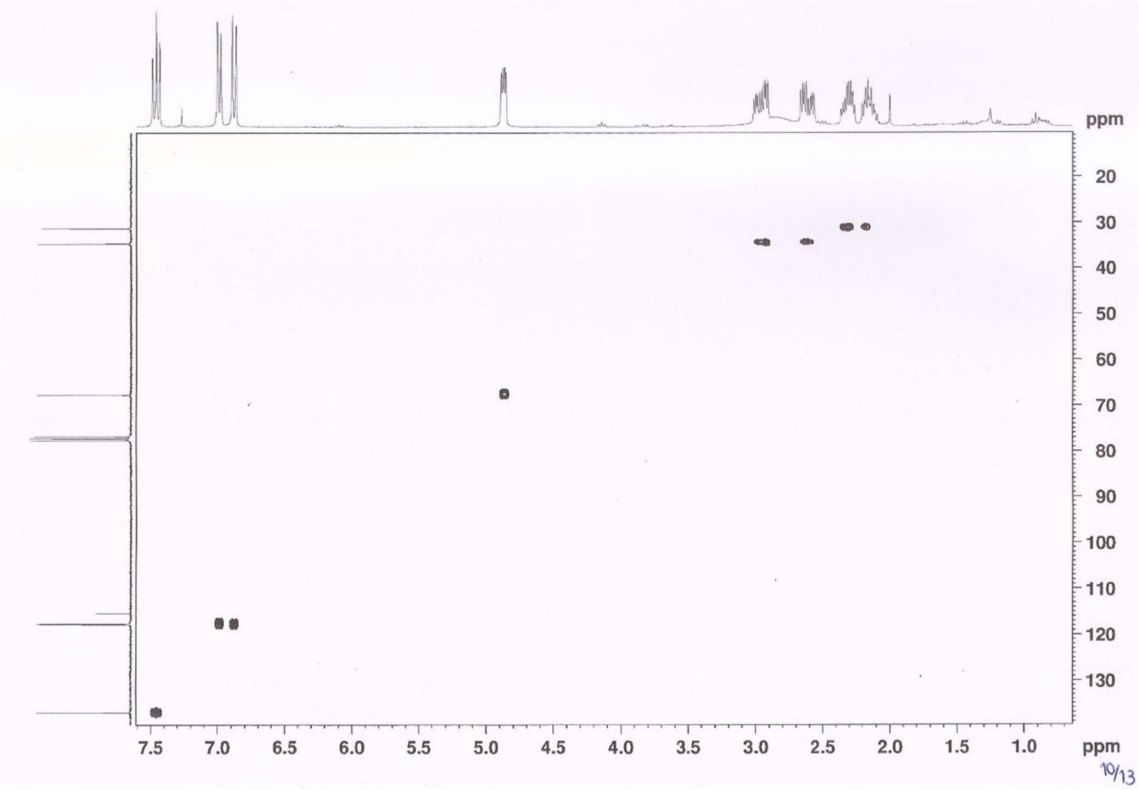

Figure S46. HMBC spectrum of **7** ($CDCl_3$, 300 MHz).

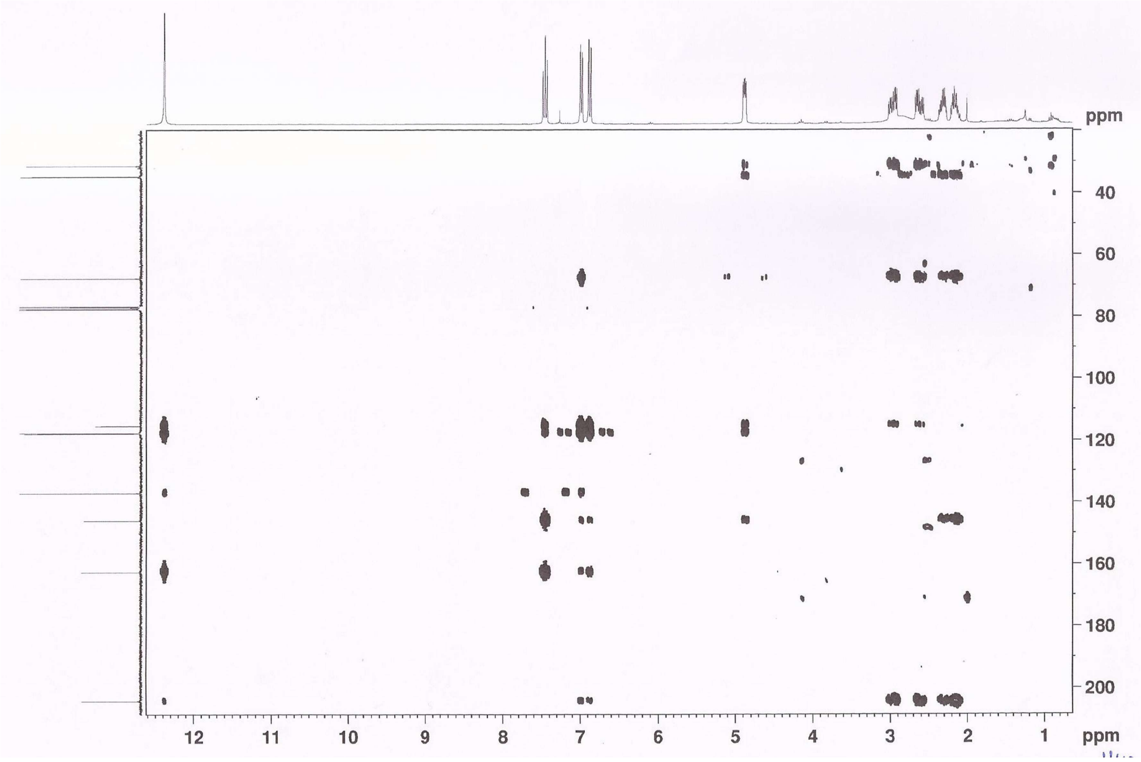

Table S1. ^{1}H and ^{13}C NMR data ($CDCl_3$, 300 and 75 MHz), COSY and HMBC for **3.**

Position	δ_C, type	δ_H, (J in Hz)	COSY	HMBC
1	171.5, CO			
3	91.6, C			
3a	146.2, C			
4	114.5 C			
5	165.8, C			
6	99.0, CH	6.49, s	H_3-11, OMe-5	C-1, 4, 5, 7, 7a
7	156.6, C			
7a	102.2, C			
8	202.8, CO			
9	24.1 CH_3	2.06, s		C-8
10	20.1, CH_3	1.78, s		C-3, 3ª, 8
11	10.0, CH_3	2.04, s	H-6	C-3a, 4, 5
OMe-5	56.4, CH_3	3.89,s	H-6	C-5
OH-7	7.81, brs			

Table S2. ^{1}H and ^{13}C NMR (300 and 75 MHz, $CDCl_3$), COSY and HMBC of **4a**.

Position	δ$_C$, type	δ$_H$, (*J* in Hz)	COSY	HMBC
1	171.6, CO			
3	92.0, C			
3a	150.0, C			
4	112.2, C			
5	165.3, C			
6	98.3, CH	6.40, s		1, 4, 5, 7, 7a
7	156.4, C			
7a	103.0, C			
8	70.7, CH	4.20, m	H_3-9	
9	17.6, CH_3	0.89, d (6.5)	H-8	C-3, 8
10	21.2, CH_3	1.76, s		C-3, 3a, 8
11	11.2, CH_3	2.07,s		C-3a, 4, 5
OMe-5	56. 3 OCH_3,	3.83, s		C-5
OH-7	-	7.93, br		
OH-8		2.73, br		

Table S3. Comparison of ^{1}H and ^{13}C NMR data of **4a** (300 and 75 MHz, $CDCl_3$) with those of (*R*)-7-hydroxy-3-((*R*)-1-hydroxyethyl)-5-methoxy-3,4-dimethylisobenzofuran-1(3*H*)-one (400 and 100 MHz, $CDCl_3$) and (*R*)-7-hydroxy-3-((*S*)-1-hydroxyethyl)-5-methoxy-3,4-dimethylisobenzofuran-1(3*H*)-one (500 and 125 MHz, $CDCl_3$)

	**4a**		(*R*)-7-hydroxy-3-((*R*)-1-hydroxyethyl)-5-methoxy-3,4-dimethylisobenzofuran-1(3*H*)-one		(*R*)-7-hydroxy-3-((*S*)-1-hydroxyethyl)-5-methoxy-3,4-dimethylisobenzofuran-1(3*H*)-one	
Position	δ$_C$, type	δ$_H$, (*J* in Hz)	δ$_C$, type	δ$_H$, (*J* in Hz)	δ$_C$, type	δ$_H$, (*J* in Hz)
1	171.6, CO		171.7, CO		171.5, CO	
3	92.0, C		92.1, C		91.3, C	
3a	150.0, C		150.1, C		149.9	
4	112.2, C		112.5, C		112.2, C	
5	165.3, C		165.6, C		165.4, C	
6	98.3, CH	6.40, s	98.5, CH	6.41, s	98.2, CH	6.43, s
7	156.4, C		156.8, C		156.5,C	
7a	103.0, C		103.3, C		103.0,C	
8	70.7, CH	4.20, m	71.2, C	4.19, q (6.5)	70.9, CH	4.22, m
9	17.6, CH_3	0.89, d (6.5)	18.1, CH_3	0.91d (6.5)	17.5, CH_3	0.93, d (6.8)
10	21.2, CH_3	1.76, s	21.5, CH_3	1.78, s	21.3, CH_3	1.81, s
11	11.2, CH_3	2.07, s	11.4, CH_3	2.09, s	11.2, CH_3	2.11, s
OMe-5	56.3, CH_3	3.83, s	56.5, CH_3	3.85, s	56.3, CH_3	3.88, s

OH-7	-	7.93, br	-	7.84,	-	7.88
OH-8		2.73, br				1.97, d (10.3)

Table S4. ^{1}H and ^{13}C NMR data (DMSO-d_6, 300 and 75 MHz), COSYand HMBC for **7.**

Position	δ_C, type	δ_H, (J in Hz)	COSY	HMBC
1	204.4, CO			
2	34.6, CH_2	2.96, ddd (17.9, 8.1, 4.8)<br>2.61, ddd (17.9, 8.3, 4.8)	H-3	C-1, 3, 4, 8a
3	31.2, CH_2	2.16, m<br>2.32, m	H-2, 4	C-1, 2, 4, 4a
4	67.6, CH	4.88, dd (7.3, 3.7)	H-3	C-2, 3, 4a, 5, 8a
4a	145.9, C			
5	117.5, CH	6.99, d (7.4)	H-6	C-1, 4, 4a, 6, 7, 8, 8a
6	137.0, CH	7.46, dd (8.3, 7.4)	H-5, 7	C-4a, 5, 7, 8, 8a
7	117.7, CH	6.88, d (8.3)	H6-	C-4, 4a, 5, 8, 8a
8	162.6, C			
8a	115.2, C			
OH-8	-	12.39, s		C-6, 7, 8, 8a
